# Integrated metagenomics identifies a crucial role for trimethylamine-producing *Lachnoclostridium* in promoting atherosclerosis

**DOI:** 10.1038/s41522-022-00273-4

**Published:** 2022-03-10

**Authors:** Yuan-Yuan Cai, Feng-Qing Huang, Xingzhen Lao, Yawen Lu, Xuejiao Gao, Raphael N. Alolga, Kunpeng Yin, Xingchen Zhou, Yun Wang, Baolin Liu, Jing Shang, Lian-Wen Qi, Jing Li

**Affiliations:** 1 School of Life Science and Technology, China Pharmaceutical University, Nanjing, 210009 China; 2 State Key Laboratory of Natural Medicines, School of traditional Chinese Pharmacy, China Pharmaceutical University, Nanjing, 210009 China; 3 Affiliated Hospital of Integrated Traditional Chinese and Western Medicine, Nanjing University of Chinese Medicine, Nanjing, 210028 China; 4 Key Laboratory of Drug Quality Control and Pharmacovigilance (China Pharmaceutical University), Ministry of Education, Nanjing, 210009 China

**Keywords:** Applied microbiology, Clinical microbiology

## Abstract

Microbial trimethylamine (TMA)-lyase activity promotes the development of atherosclerosis by generating of TMA, the precursor of TMA N-oxide (TMAO). TMAO is well documented, but same can not be said of TMA-producing bacteria. This work aimed to identify TMA-producing genera in human intestinal microbiota. We retrieved the genomes of human-associated microorganisms from the Human Microbiome Project database comprising 1751 genomes, Unified Human Gastrointestinal Genome collection consisting 4644 gut prokaryotes, recapitulated 4930 species-level genome bins and public gut metagenomic data of 2134 individuals from 11 populations. By sequence searching, 216 TMA-lyase-containing species from 102 genera were found to contain the homologous sequences of *cntA*/*B*, *yeaW*/*X,* and/or *cutC*/*D*. We identified 13 strains from 5 genera with *cntA* sequences, and 30 strains from 14 genera with *cutC* showing detectable relative abundance in healthy individuals. *Lachnoclostridium* (*p* = 2.9e−05) and *Clostridium* (*p* = 5.8e−04), the two most abundant *cutC*-containing genera, were found to be much higher in atherosclerotic patients compared with healthy persons. Upon incubation with choline (substrate), *L. saccharolyticum* effectively transformed it to TMA at a rate higher than 98.7% while that for *C. sporogenes* was 63.8–67.5% as detected by liquid chromatography-triple quadrupole mass spectrometry. In vivo studies further showed that treatment of *L. saccharolyticum* and choline promoted a significant increase in TMAO level in the serum of *ApoE*^−/−^ mice with obvious accumulation of aortic plaque in same. This study discloses the significance and efficiency of the gut bacterium *L. saccharolyticum* in transforming choline to TMA and consequently promoting the development of atherosclerosis.

## Introduction

Numerous studies on intestinal microbiota over the past ten years have confirmed their pivotal roles in human health and diseases^1–3^. Cardiovascular diseases remain the leading cause of mortality and morbidity worldwide, accounting for 17.8 million deaths globally^4^. Accumulation of cholesterol on the arterial wall, immune responses, and chronic inflammation have been reported as major biological events in atherosclerosis^5^. Recently, the gut microbiota as a whole, has recently been found to be an important contributor to the progression of cardiovascular diseases^1,6,7^. The gut microbiota can regulate host cholesterol homeostasis aside from the host genetic contribution^8^. Increasing evidence demonstrates that gut microbiota-derived metabolites, such as short-chain fatty acids^9^, secondary bile acids^10^, and lipopolysaccharide^11^, play crucial roles in the development of atherosclerosis.

Trimethylamine (TMA) is a small molecular weight byproduct of intestinal microbial metabolism of dietary choline, carnitine, and phosphatidylcholines^12–14^. TMA is converted to trimethylamine-N-oxide (TMAO) in the liver via flavin-containing monooxygenase 3^15–17^. A link between TMAO and cardiovascular diseases first emerged in 2011, when investigators found a dose-dependent association between plasma concentrations of TMAO and risk of cardiovascular diseases among persons with heart diseases^15^. A growing number of studies have since confirmed plasma TMAO as an independent risk factor for atherosclerosis, thrombus formation^18^, and myocardial infarction^7,16^.

The probable mechanisms by which TMAO contributes to cardiovascular diseases could involve enhanced foam cell formation^15^, enhanced activation of platelets with increased calcium release^18^, and adverse ventricular remodeling^19^. In addition, TMAO partly accounts for exacerbated atherosclerosis by promoting forward cholesterol transport and by inhibiting the reverse transport of same^13,15,20^.

TMA, a precursor of TMAO, is produced by gut commensals using three different enzyme complexes. Choline TMA-lyase (*cutC/D*) which was discovered from the anaerobic sulfate-reducing *Desulfovibrio desulfuricans*, uses choline to produce TMA^14^. *CutC* is a specific glycyl radical enzyme, while *cutD* is its activator^14,21^. The two-component Rieske-type oxygenase/reductase (*cntA/B*), which uses carnitine to produce TMA^12^, is found in *Acinetobacter calcoaceticus*^22^ and *Serratia marcescens*^23^. *YeaW/X*, the sequence of which is similar to *cntA/B*, is another Rieske-type oxygenase/reductase that uses γ-butyrobetaine as a substrate to generate TMA^12,13^. In spite of available credible information on TMA-producing bacteria^24–26^ and their importance in human health, little is known about their abundance in different populations, their relationship with diseases, and TMA conversion abilities.

In this study, using an integrated metagenomic approach, we analyzed reference genomes from the three reference resources^27,28^, 12 metagenomic datasets from public metagenomic databases and biologically validated our findings with an *Apoe*^−/−^ mice model. We sought to investigate: (1) the bacteria in the human gut microbiota that encode TMA-lyase in their genomes (2) the abundances and activities of these TMA-producing bacteria in the gut and (3) whether or not the TMA-producing bacteria increase the risk of developing atherosclerosis.

## Results

### Taxonomic identification of TMA-producing bacteria

**Fig. 1:**
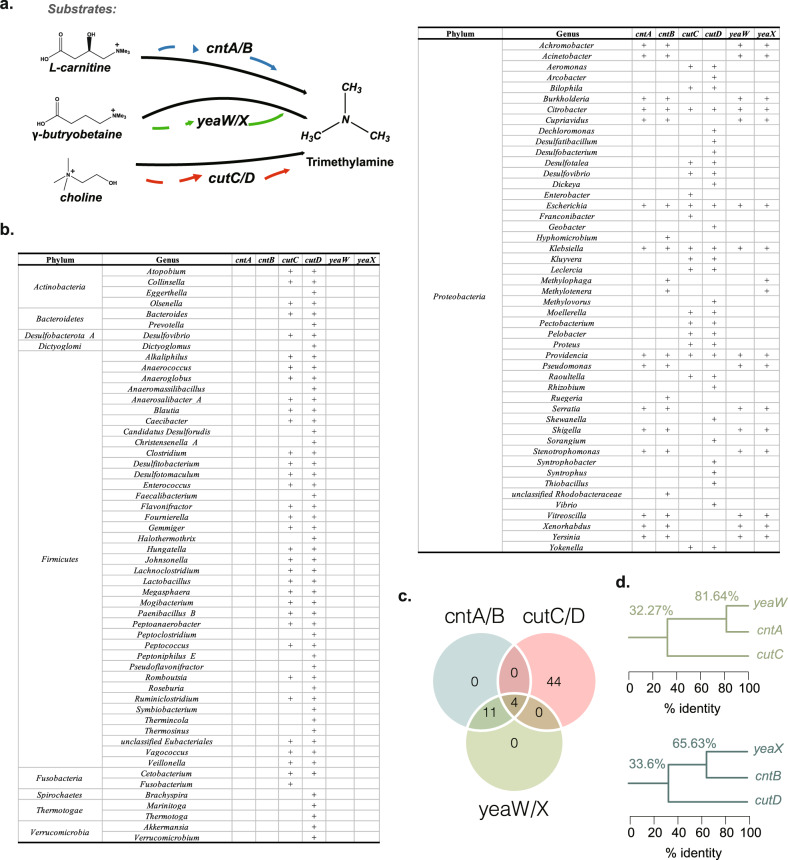
Taxonomic distribution of TMA-producing bacteria. **a** Schematic diagram of three microbial transformation pathways of TMA-lyases, *cntA/B*, *yeaW/X,* and *cutC/D*. **b** The phyla and genera distribution of *cntA/B, cutC/D,* and *yeaW/X*. The plus signs indicate that the genus contains the genes which encoded the corresponding TMA-lyase. **c** Venn diagram analysis of the coexisting genera distribution of *cntA/B, cutC/D,* and *yeaW/X*. **d** Homology trees of three TMA-lyases and their corresponding activators. The numbers on the corner represent the average sequence identities between the TMA-lyases connected by the branch. TMA: trimethylamine.

The gut microbiota is known to convert carnitine, betaine, and choline to TMA by utilizing three TMA-lyase complexes, *cntA/B*, *yeaW/X,* and *cutC/D*, respectively. *CntA*, *yeaW,* and *cutC* are TMA-lyases, while *cntB*, *yeaX,* and *cutD* are their activators (Fig. 1a). We identified the TMA-lyase sequences of the TMA-producing bacteria based on the three reference resources.

The final 885 TMA-lyase reference sequences were identified from three resources, 451 from the Human Microbiome Project database, 216 from the Unified Human Gastrointestinal Genome, and 218 reference sequences from 4930 species-level genome bins by taking the initial 983 sequences as query (Supplementary Table 1). These sequences were distributed in 216 bacteria species from 102 genera as shown in Supplementary Table 2. Specifically, *cntA* existed in 15 genera, *cntB* in 20 genera, *yeaW* in 15 genera, and *yeaX* in 17 genera (Fig. 1b). All these genera belong to the phylum of *Proteobacteria* (Fig. 1b). *CutC* was distributed in 3 genera from *Actinobacteria*, 27 from *Firmicutes*, 18 from *Proteobacteria* and 4 from other phyla like *Bacteroidetes*, *Desulfobacterota_A,* and *Fusobacteria*. *CutD* was distributed in 4 genera from *Actinobacteria*, 39 from *Firmicutes*, 30 from *Proteobacteria* and 10 from other phyla like *Bacteroidetes*, *Desulfobacterota_A, Dictyoglomi*, *Fusobacteria, Spirochaetes, Thermotogae,* and *Verrucomicrobia* (Fig. 1b). Furthermore, *cntA/B* and *yeaW/X* coexisted in 15 genera, while *cutC/D* coexisted in 48 genera (Fig. 1c).

It is worth noting that *cntA*/*B* and *yeaW*/*X* coexisted in 49 species (Supplementary Table 2). For sequence similarity, the average sequence identity was 81.64% for *cntA* and *yeaW*, and 65.63% for *cntB* and *yeaX* (Fig. 1d). Compared to *cntA/B* and *yeaW/X*, the sequence identity of *cutC*/*D* was relatively low; 32.27% for *cutC* and *cntA/yeaW*, and 33.60% for *cutD* and *cntB/yeaX* (Fig. 1d). Since the TMA-lyases *cntA*/*yeaW*/*cutC* usually coexisted with their corresponding activators *cntB*/*yeaX*/*cutD* and there was high homologous similarity between *cntA*/*B* and *yeaW*/*X* rather than *cutC/D*, we focused our attention on *cntA* and *cutC* in subsequent analysis.

### Relative abundance of *cntA* and *cutC* in the human intestine

**Fig. 2:**
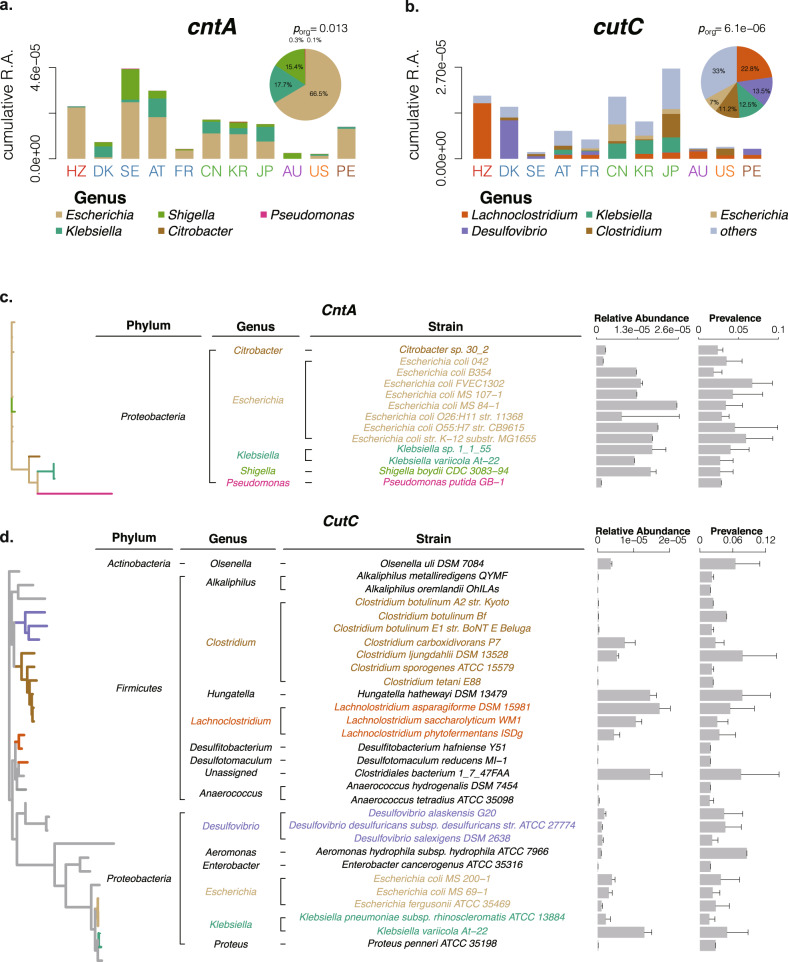
R.A. distribution and taxonomic identification of *cntA* and *cutC*. R.A. distribution of *cntA* (**a**) and *cutC* (**b**) in 11 populations from six continents. Stack columns display the cumulative R.A. of different genera and pie charts in the corner show the distribution of the abundant genera. The detailed 11 populations from six continents include: Africa: HZ (the Hadza ethnic group of Tanzania); Asia: CN (China), JP (Japan), and KR (South Korea); Europe: DK (Denmark), SE (Sweden), AT (Austria), and FR (France); Oceania: AU (Australia); North America US (the United States); South America: PE (Peru). Significant differences in R.A. were conducted by Kruskal–Wallis rank sum test *p*_org_. Taxonomic identification of *cntA* (**c**) and *cutC* (**d**). The left panels show the phylogenetic tree and the highlighted branches correspond to the colors of different genera on the middle panel. The bar charts on the right panel indicate the average R.A. and the prevalence of *cntA* and *cutC* among different intestinal strains. R.A.: relative abundance. Error bars denote the means ± s.e.m.

An integrated reference metagenomic sequence dataset was acquired from several public cohorts (Supplementary Table 3). Multivariable-adjusted analysis showed that population played a major role in the relative abundance (R.A.) of *cntA* (27.15% contribution to total variations) and *cutC* (21.02% contribution to total variations, Supplementary Fig. 1). A total of 645 healthy individuals worldwide were included to figure out the R.A. of TMA-producing bacteria with sequence alignment. Five genera containing *cntA* were found in the human intestine, and *Escherichia* occupied 66.5% followed by *Klebsiella* with 17.7% and then *Shigella* with 15.4% (Fig. 2a). The cumulative R.A. of *cntA* varied widely among populations (*p*_org_ = 0.013). Swedish exhibited the highest cumulative R.A. of *cntA* (4.55 × 10^−5^), while Americans exhibited the lowest cumulative R.A. (2.48 × 10^−6^) (Fig. 2a). For *cutC*, the top five most prevalent *cutC*-carrying genera were *Lachnoclostridium* (22.8%), *Desulfovibrio* (13.5%), *Klebsiella* (12.5%), *Clostridium* (11.2%) and *Escherichia* (7%) (Fig. 2b). The cumulative R.A. of *cutC* also varied largely among populations (*p*_org_ = 6.1e−06). Japanese showed the highest *cutC* cumulative R.A. (2.66 × 10^−5^), while Swedish showed the lowest cumulative R.A. (2.02 × 10^−6^).

Subsequence analysis of the TMA-producing bacterial strains was based on the Human Microbiome Project which has defined strain taxonomic information among the three reference sources. We conducted phylogenetic analysis of *cntA* and *cutC* in candidate TMA-producing bacterial strains. Totally, 13 strains were found to have detectable R.A. containing *cntA*, 8 of which belonged to the dominant genera *Escherichia* (Fig. 2c, left panel). In the phylogenetic classification, the *cntA* from *Shigella* was close to *Escherichia*. Two strains from *Klebsiella* and one strain from *Pseudomonas* were farther on the phylogenetic tree, suggesting that their genetic sequences differ widely. The R.A. of *cntA* in the 13 strains ranged from 1.62 × 10^−6^ to 2.55 × 10^−5^ and the prevalence ranged from 1.90 to 6.72% (Fig. 2c, right panel).

*CutC* existed in 30 strains with detectable R.A. in three phyla, *Actinobacteria*, *Firmicutes,* and *Proteobacteria*. There were three main branches in the phylogenetic tree of *cutC*. The first branch was mainly from *Clostridium* (R.A., 3.71 × 10^−8^–7.54 × 10^−6^ and prevalence, 2.20–7.80%). The next branch was mainly from *Lachnoclostridium* (R.A., 4.54 × 10^−6^–1.72 × 10^−5^ and prevalence, 3.18–5.53%) and *Desulfovibrio* (R.A., 3.80 × 10^−7^–4.61 × 10^−7^ and prevalence, 2.24–4.66%). The third branch was mainly from *Escherichia* (R.A., 1.06 × 10^−6^–3.99 × 10^−6^ and prevalence, 1.30–3.41%) and *Klebsiella* (R.A., 2.25 × 10^−6^–1.30 × 10^−5^ and prevalence, 1.73–4.97%). Among them, the highest genus containing *cutC* was *Lachnoclostridium* (Fig. 2d).

### Correlation of *cntA* and *cutC* with various diseases

**Fig. 3:**
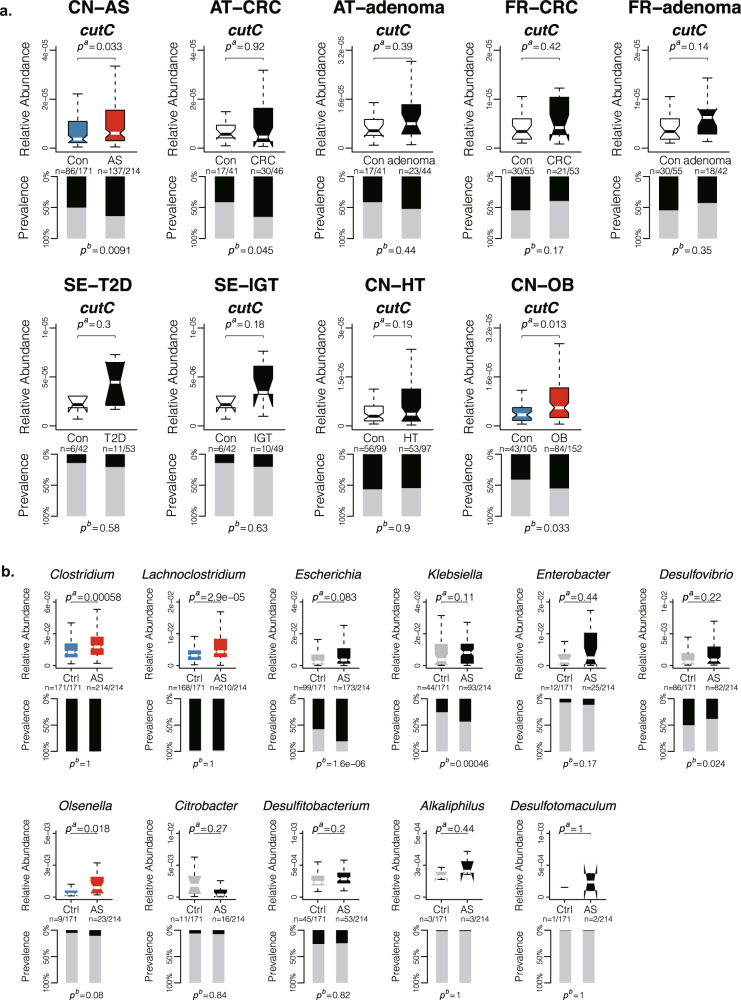
Correlation of *cutC* and *cutC*-containing genera with various disease. **a** The upper panel show the R.A. distribution of *cutC* and the lower panel show their prevalence in the gut microbiome of healthy individuals and patients with various diseases. **b** The upper panel show the R.A. of *cutC*-containing genera and the lower panel shows their prevalence in the gut microbiome of patients with AS compared with healthy controls. The bounds denote the interquartile range between the first and third quartiles and the center line denotes the median. The whiskers denote the lowest and highest values. The black column in down panels represents numbers of individuals whose R.A. can be quantified, while the gray column represents individuals whose R.A. was 0. Significant differences in R.A. were conducted by Mann–Whitney–Wilcoxon test (*p*^a^), color boxplots indicate *p* < 0.05. Significant differences in prevalence were conducted by Chi-squared test (*p*^b^). CN-AS: atherosclerosis from China; AT-CRC: colorectal cancer from Austria; AT-adenoma: adenoma from Austria; FR-CRC: colorectal cancer from France; FR-adenoma: adenoma from France; SE-T2D: type 2 diabetes from Sweden; SE-IGT: impaired glucose tolerance from Sweden; CN-HT: hypertension from China; CN-OB: obesity from China; R.A.: relative abundance; AS: atherosclerosis.

We then explored the correlation of *cntA* and *cutC* with various diseases such as, adenoma, colorectal cancer, impaired glucose tolerance, type 2 diabetes, cardiovascular disease, hypertension, and obesity using several public case-control gut metagenomic datasets (Supplementary Table 3). Interestingly, the R.A. of *cntA* showed no significant differences in the various disease groups compared with healthy individuals. There was an elevated trend but no statistically significant difference between persons with atherosclerosis and the healthy individuals (Supplementary Fig. 2). Surprisingly, *cutC* showed significantly increased R.A. in the patients with atherosclerosis (Mann–Whitney U test *p*^*a*^ = 0.033) and a significant difference in prevalence of same (Chi-squared test *p*^*b*^ = 0.0091) compared with healthy individuals. Moreover, *cutC* showed significantly increased R.A. in the patients with obesity (Mann–Whitney U test *p*^*a*^ = 0.013) and a significant difference in prevalence (Chi-squared test *p*^*b*^ = 0.033) compared with healthy individuals (Fig. 3a). No significant differences in the R.A. of *cutC* were observed in other diseases compared with the healthy controls. The prevalence of *cutC* was significant higher in patients with colorectal cancer compared with healthy individuals (Chi-squared test *p*^*b*^ = 0.045).

In further analysis of the correlation between the risk of atherosclerosis and *cutC*-containing genera, we observed that the R.A. of *Lachnoclostridium* (Mann–Whitney U test *p*^*a*^ = 2.9e−05), *Clostridium* (*p*^*a*^ = 5.8e−04) and *Olsenella* (*p*^*a*^ = 0.018) were significantly increased in patients with atherosclerosis compared with the healthy persons (Fig. 3b). Other genera showed no significant differences in the R.A. between the atherosclerotic patients and healthy individuals (Fig. 3b). *Lachnoclostridium* and *Clostridium* showed highest prevalence in this cohort. The prevalence of *Escherichia* (Chi-squared test *p*^*b*^ = 1.6e−06), *Klebsiella* (*p*^*b*^ = 0.00046) and *Desulfovibrio* (*p*^*b*^ = 0.024) were significant different. The genera *Anaerococcus*, *Hungatella,* and *Proteus* were not detected in this dataset.

### In silico and in vitro comparisons of candidate TMA-producing genera

**Fig. 4:**
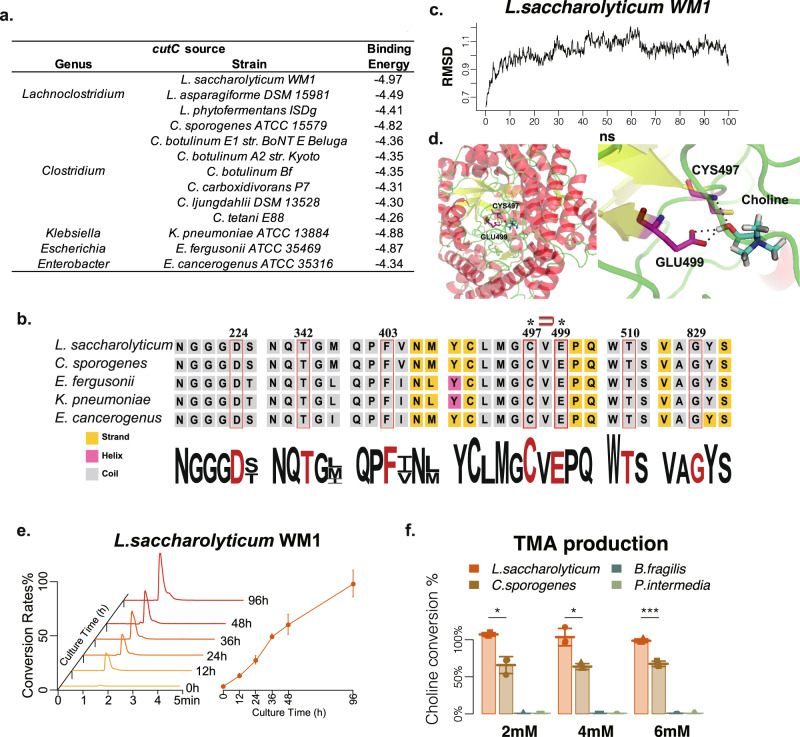
In silico and in vitro comparisons of candidate TMA-producing genera. **a** The binding energies of *cutC*-containing strains and choline based on homologous modeling. **b** The 7 site residues of *cutC* in TMA-producing genera. Conserved amino acid sites are marked in red boxes with amino acid locations indicated above. The secondary structure is represented by background color, yellow represents beta folding, and purple represents alpha spiral. The asterisks indicate hydrogen bond connections with choline, and the paper clip symbol in the middle indicate the presence of a card-issue structure. **c** The binding conformation complex of *cutC* from *L. saccharolyticum* and choline was stable at 100 ns dynamic equilibrium. **d** The AutoDock predicted binding conformation complex (from *L. saccharolyticum*), within which the protein is shown as cartoon and choline as sticks. The residues that have important hydrogen-bond interactions with choline are labeled and these hydrogen bonds are represented as dashed lines. **e** The typical chromatogram of TMA produced by *L. saccharolyticum* incubated with choline at different times are shown in the left panel, and the corresponding conversion rate curve is shown in the right panel. **f** Barplots show the conversion rates of strains from TMA-producing genera based on various choline concentrations, 2, 4, and 6 mM (*n* = 3). TMA production was assessed using LC-MS. Error bars denote the means ± s.e.m. *p < 0.05, **p < 0.01, ***p < 0.001, differences were conducted by Mann–Whitney–Wilcoxon test.

Since *cutC* but not *cntA* was significantly increased in patients with atherosclerosis, we then compared the TMA-producing ability of 5 candidate *cutC*-containing genera with high R.A. We performed homologous modeling, molecular docking, and dynamic simulation analysis between choline and *cutC* protein from different strains (specific sequence is shown in Supplementary Table 4). The homologous modeled structure of *cutC* from *L. saccharolyticum WM1* gave the lowest binding energy (-4.97 kJ/mol) with choline compared with *L. asparagiforme DSM 15981* (−4.49 KJ/mol) and *L. phytofermentans ISDg* (−4.41 KJ/mol). In addition, the binding energy between choline and *cutC* from *L. saccharolyticum WM1* was lower than the other four *cutC*-containing genera (from −4.26 to −4.82 KJ/mol) (Fig. 4a). The seven active site residues of *cutC* were all conserved among these *cutC*-containing genera (Fig. 4b)^29^. However, the site residues close to the seven active sites, such as V225, M344, V404, and M406, appeared to differ among these genera, possibly accounting for the differences in the *cutC* enzyme activity. The binding conformation between choline and *cutC* from *L. saccharolyticum WM1* was relatively stable within 100 ns by the dynamic simulation (Fig. 4c). In this binding conformation, choline exhibited three hydrogen bonds with two key residues, i.e., Cys497 and Glu499 (Fig. 4d). These findings demonstrated that *L. saccharolyticum WM1* exhibited favorable binding affinity to substrate choline. The binding conformation of *cutC* from other candidate *cutC*-containing genera are showed in Supplementary Fig. 3.

*L. saccharolyticum WM1* was chosen for subsequent in vitro- and in vivo- experiments. The TMA-producing activities of *several cutC*-containing strains were then investigated by incubating each strain with the substrate choline. The concentration of TMA was accurately quantified using an isotope-labeled internal standard method by liquid chromatography-triple quadrupole mass spectrometry. *L. saccharolyticum WM1*, effectively converted choline to TMA in a time-dependent manner at 12, 24, 36, 48, and 96 h (Fig. 4e). The conversion rates of *L. saccharolyticum WM1* for various concentrations of choline (2 mM, 4 mM and 6 mM) were higher than 98.68% (Fig. 4f). *C. sporogenes* BNCC 104015 showed moderate conversion rates for choline (63.79–67.51%). *B. fragilis* BNCC 352061 and *P. intermedia* BNCC336948, two negative control strains without *cutC* enzymes, showed no capability in transforming choline to TMA (less than 0.1%) (Fig. 4f).

### *L. saccharolyticum* promotes the development of atherosclerosis in *ApoE*^−/−^ mice

**Fig. 5:**
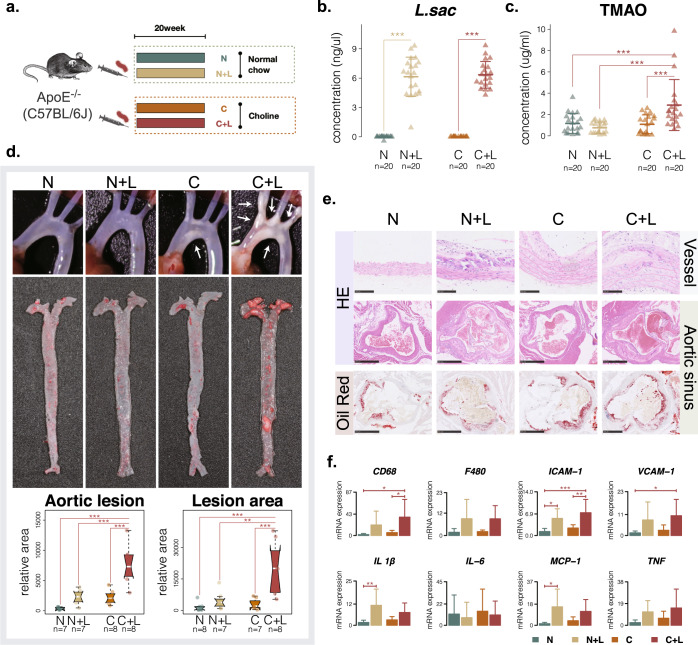
*L. saccharolyticum* increased TMAO level and accelerated atherosclerosis in *ApoE*^−/−^ mice. **a** Schematic diagram of the animal experiments. **b** The concentration of *L. saccharolyticum* in fecal samples of the four groups (*n* = 20). **c** The serum levels of TMAO in the four groups (*n* = 20). **d** Atherosclerotic plaques of mice are shown in pictures, and the quantitative results of the plaque area are shown in the box plot (*n* = 7 or 8). The bounds denote the interquartile range between the first and third quartiles and the center line denotes the median. The whiskers denote the lowest and highest values. **e** Staining results of tissue sections. The upper panel shows the HE results of vessel tissue, the middle panel shows the HE results of aortic sinus, and the lower panel shows the Oil Red results of aortic sinus. The scale bars of vessels denote the 100 μm, and the scale bars of aortic sinus denote the 500 μm. **f** The mRNA expression of related cytokines in vascular endothelial cell tissue (*n* = 8). Error bars denote the means ± s.e.m. *p < 0.05, **p < 0.01, ***p < 0.001, the differences among groups were analyzed by single-factor ANOVA corrected with Tukey HSD test. N: normal group, mice fed with standard chow control diet and gavaged with the sterile medium; N+L: normal + *L. saccharolyticum* group, mice fed standard chow and gavaged live *L. saccharolyticum* at a dose of 5 × 10^8^ CFUs/100 μl; C: choline group, mice fed with standard chow supplemented with 1.0% choline (Sigma-Aldrich, USA) and gavaged with the sterile medium; C+L: choline + *L. saccharolyticum* group, mice fed with standard chow supplemented with 1.0% choline and gavaged with live *L. saccharolyticum* at a dose of 5 × 10^8^ CFUs/100 μl.

After showing that *L. saccharolyticum* effectively converted choline to TMA by in vitro incubation, we then investigated the possible implication of *L. saccharolyticum* in atherosclerosis in *ApoE*^−/−^ mice. We compared the atherosclerotic phenotypes of mice in the following four groups: normal group, normal + *L. saccharolyticum* group, choline group, and choline + *L. saccharolyticum* group (Fig. 5a). *L. saccharolyticum* administration notably elevated the level of *L. saccharolyticum* in fecal samples (Fig. 5b). The serum levels of TMAO, the downstream metabolite of TMA, significantly increased in the choline + *L. saccharolyticum* group compared with the normal group (*p* = 9.9e−04), normal + *L. saccharolyticum* group (*p* = 3.4e−05) and choline group (*p* = 5.3e−04) (Fig. 5c). Compared with the normal control, the plaques and the stenosis of the blood vessels showed an increased trend in the *L. saccharolyticum* group and the choline group. The plaques and the stenosis of aortic arch were greatly pronounced in the choline + *L. saccharolyticum* group (Fig. 5d). More importantly, the level of *L. saccharolyticum* in the fecal samples was positively correlated with TMAO level in serum (*R* = 0.31, *p* = 0.0052), aortic lesion area (*R* = 0.54, *p* = 0.00024) and vessel lesion area (*R* = 0.48, *p* = 0.0085, Supplementary Fig. 4a). Administration of choline increased the thickness of the vessel wall and inflammatory cells infiltration and lipid deposition (Fig. 5e). These changes were more obvious in the choline + *L. saccharolyticum-*treated mice (Fig. 5e). *L. saccharolyticum* and choline administration triggered inflammation in the vessel, as indicated by gene inductions of *Il-1β*, *Tnf-α*, *Icam-1* and *Vcam-1*, *Mcp-1*, *Cd68,* and *F480* (Fig. 5f). Similarly, *L. saccharolyticum* administration increased circulating contents of FFAs, while other metabolic parameters remained unaffected (Supplementary Fig. 4b). These results indicated that *L. saccharolyticum* abundance impaired the aorta and favored the formation of atherosclerosis, probably due to the increased production of TMAO.

## Discussion

In this work, we implemented an integrated metagenomics approach combined with bioinformatics and in vitro/in vivo validation to characterize a key role for TMA-lyase bacteria in the promotion of atherosclerotic lesion formation. The major findings of this study include the following: (1) We conducted a taxonomic analysis based on three reference sources and identified 216 TMA-producing species from 102 genera. (2) By analyzing 2134 individuals from 11 populations using public metagenomics data, we identified 13 *cntA*-containing and 30 *cutC*-containing bacterial strains. Correlation analysis of TMA-lyase bacteria and various diseases showed that *Lachnoclostridium*, the most abundant *cutC*-containing genus in healthy individuals, was significantly increased in atherosclerotic patients. (3) *L. saccharolyticum* WM1, a representative strain of *Lachnoclostridium*, effectively converted choline to TMA at a transformation rate near 100%, much higher than 63% for *C. sporogenes*. This strain was found to elevate serum TMAO and promote the formation of atherosclerosis in *ApoE*^−/−^ mice when co-administered with choline.

Uncovering the TMA-producing bacteria and the conversion capabilities of the human gut microbiota may predict risk of developing atherosclerosis^3^. Falony et al. identified 102 genomes showing TMA-producing potential by mining public genomic databases^25^. With emerging updated data on human metagenomics, we performed a more comprehensive taxonomic analysis and identified 216 TMA-producing species from 102 genera. Romano and colleagues identified 8 strains from 79 human intestinal isolates showing significant transformation of choline to TMA^26^. In line with this observation, 7 of the 8 strains were found in the 30 *cutC*-containing strains identified in the present study. The remaining one isolate, thus *E. tarda* ATCC 23685 that was neglected in this study, as it does not appear to contain *cutC* genes.

It is well established that the risk of developing cardiovascular diseases is associated with genetic variations^30^, dietary factors^31^, and TMAO concentration in blood^15,16^. Factors that determine the TMA-lyase abundance in the human gut metagenome are still not fully characterized. We observed that population type played a major role in the R.A. of *cntA* and *cutC*, age had a slight impact, and BMI and gender had no observable effect. Population structure is also associated with several confounding factors such as environment, dietary factors, and host genetic structures. Because of the significance of population structure in the R.A. of TMA-lyase, it was crucial to employ the worldwide open public datasets of the 11 populations captured in this work in order to avoid biases.

In the correlation analysis, we found that the R.A. of *cntA* showed no significant differences between atherosclerotic patients and healthy individuals, an observation that is consistent with a previous study^6^. This finding may be attributed to the limited depth of sequencing and query set. In the prevalence analysis of *cutC*, we found a significant increase in the colorectal cancer patients which is consistent with the findings of Thomas et al. who found *cutC* to be overabundant in colorectal cancer^32^. It is worth noting that the R.A. of *cutC* was markedly elevated in atherosclerotic patients but not in other diseases. Our result is contrary to that of Jie et al. who reported no significant difference in the R.A. of *cutC* between atherosclerotic patients and healthy persons using the same cohort^6^. This divergence in final outcome could be attributed to the 575 query sequences of the *cutC* enzyme retrieved in this work juxtaposed with the 13 sequences employed by Jie et al. Microbial transplantation of *cutC*-containing commensals could promote atherosclerosis development^33^ and thrombosis^18^. Skye et al. showed that transplantation of *Clostridium sporogenes*, a *cutC*-expressing human commensal, was sufficient to transmit thrombosis potential in a host^34^. Consistently, we confirmed that *Clostridium sporogenes* transformed choline to TMA in vitro at a moderate conversation rate of 63.8–67.5%. More importantly, this work is the first to identify *Lachnoclostridium* as the most abundant *cutC*-containing genus, and show that *L. saccharolyticum* converts choline to TMA at a rate that is almost 100%.

Investigations in animal models are key to confirming the functions of bacteria. Backhed et al. observed that the impact of the gut microbiota on atherosclerosis was dietary dependent, and single choline supplementation did not affect the plaque size and aortic lesions^35^. This finding is in agreement with our results in the sense that, single treatment of the *ApoE*^−/−^ mice with choline or *L. saccharolyticum* showed minor effects on the plaque size, but co-administration of the two greatly promoted the atherosclerotic lesion formation. Lipids in general constitute an important risk factor in the development of atherosclerosis. Our avoidance of high-fat diet in our animal model may have been responsible for the observed non-significant changes in the serum cholesterol levels.

This study has some limitations. (1) The integrated metagenomic analysis based on public metagenome datasets constitutes the primary limitation. All datasets were subjected to the same pipeline in conducting the raw sequencing data, but there might be some biases due to the different quality of each dataset, such as sequencing depth and sample sizes. (2) The effect of *Lachnoclostridium* on the progress of atherosclerosis was verified using a mouse model, thus a prospective cohort study is necessary in the further studies.

In conclusion, our work provides an integrated metagenomic analysis of the TMA-producing bacteria in the human gut and reports for the first time a TMA-producing genus, *Lachnoclostridium*, which was significantly abundant in atherosclerotic patients. Specifically, we verified that *L. saccharolyticum* WM1 could produce TMA in vitro, increase the TMAO level in serum, and accelerate plaque formation in vivo. Hence, targeting *Lachnoclostridium* might serve as a potential therapeutic target for the treatment of atherosclerosis.

## Methods

### Bioinformatic identification of the TMA-lyase sequences

The first reference genomes resource was from the Human Microbiome Project in September 2014 which contained 1751 bacterial strains covering 1253 species. The second reference genomes resource, thus, the unified Human Gastrointestinal Genome, comprised of 204,938 nonredundant genomes from 4644 gut prokaryotes^36^, and the third reference genomes resource contained recapitulated 4930 species-level genome bins^37^ (Supplementary Fig. 5). The genes and related proteins of these bacterial genomes were predicted by MetaGeneMark (v2.8)^38^, and the taxonomic information of these genes/proteins were directly extracted from their respective strains.

The query sequences of choline-TMA-lyase (*cutC/D*) were collected from the RefSeq database of the National Center for Biotechnology Information database using the keywords “choline trimethylamine-lyase” (575 sequences) and “choline TMA-lyase-activating enzyme” (347 sequences). Carnitine-TMA-lyase (6 sequences for *cntA*; 6 for *cntB*)^12^ were collected from the original CaiT protein sequence (CAA52110)^39^. γ-butyrobetaine-TMA-lyase (30 sequences for *yeaW*; 19 for *yeaX*) were collected with the original sequences (*yeaW*: dioxygenase, GeneID 6060925; *yeaX*: oxidoreductase, GeneID 6060982)^13^. An initial total of 983 TMA-lyase sequences were taken as query and BLASTP was used to search for candidates with parameters of e-value of 1e−5 and sequence identity of 45% (Supplementary Fig. 6) as cutoff in the reference genomes resources.

### Public metagenomic sequence datasets

The public metagenomic sequence data of individuals were collected from 11 populations from six continents, including Hadza^40,41^ (PRJNA278393, pre-agricultural communities in Tanzania), Chinese^42^ (PRJNA422434), South Korean^43^ (PRJEB1690), Japanese^44^ (PRJDB3601), Australian (PRJEB6092), Austrian^45^ (PRJEB7774), Dane^46^ (PRJEB2054), French^47^ (PRJEB6070), Swedish^48^ (PRJEB1786), Peruvian^49^ (PRJNA268964), and American^27^ (PRJNA275349 and PRJNA48479). A restriction criterion was used to construct metagenomic datasets of healthy individuals from each country. The individuals with age >3 and BMI < 30 were included and subjects were excluded if they had definite diseases like inflammatory bowel disease, liver cirrhosis, and colorectal cancer.

Public metagenomic datasets of persons with different diseases were included to study the differences in the relative abundance (R.A.) of the TMA-lyase between healthy persons and the persons with diseases, such as colorectal cancer, adenoma, type 2 diabetes, impaired glucose tolerance, hypertension, obesity and atherosclerosis (ERP023788 from the European Bioinformatics Institute database)^6^.

A total of 2134 individuals were downloaded with their accession codes and the R.A. at different taxonomic levels in each cohort calculated (Supplementary Table 3).

### Metagenomic analysis of public metagenome cohorts

All raw reads were assessed and filtered using the FASTX-Toolkit (v 0.0.13) with parameters: “-Q33 -q 20 -p 80”, and high-quality microbiome sequencing reads were assembled with SOAPdenovo2 (v 2.04)^50^ using the parameters: “-avg ins 250 -K 63 -k 45 -R Y -M 3”. After assembling process, contigs with at least 500 bp were further used to predict the genes using MetaGeneMark (v2.8)^38^ with the parameters: “gmhmmp -a -d -f G -m MetaGeneMark_v1.mod”. A non-redundant protein set was then constructed by pair-wise comparison of all protein sequences within populations using BLAT (v 35×1)^51^ at 95% identity and 90% overlapping thresholds.

Protein sequences were aligned to the NCBI-NR database using BLASTP (v 2.2.29) with the parameters: “-e-value 1e−5”. The taxonomic assignments and functional annotations were constructed in MEGAN (v 5.2.3) with lowest common ancestor algorithms. The high-quality reads from each individual were aligned against the gene catalog using SOAPdenovo2 (v 2.04) with the parameters: “-r 2 -m 150 × 350 -v 5”. The quantification of each protein sequence of each individual was based on two steps: (i) Calculation of the entire copies of each gene with correct insert-size; (ii) Calculation of the R.A. of each gene in each individual, only if the other reads mapped outside the genic region, as previously described in detail^42^. The cumulative R.A. was calculated by the sum of R.A. of each genus in each population. TMA-lyase was identified in the above population datasets using BLASTP with the parameters: “-evalue 1e-5 -outfmt 0 -max_target_seqs 3” and 885 query sequences of e-value at 1e−5 and 80% sequence identity as the cutoff values. Persons without zero R.A. were employed for the comparisons within the various cohorts.

### Phylogenetic tree

The protein sequences of *cntA* and *cutC* were aligned using mafft v7.455^52^, and the resultant multiple sequences were trimmed for poorly aligned positions with Gblock 0.91b^53^. RAxML v8.2.12^54^ was used to build the most likely phylogenetic tree of genomes with the parameters “-m GTRCAT” and protein with the parameters “-m PROTGAMMAILGX”. R package “ggtree”^55^ was used to construct the phylogenetic tree.

### Homology modeling, molecular docking, and molecular dynamics

Protein Homology/analogY Recognition Engine V 2.04^56^ based on intensive mode was used to predict the homologous structures of *cutC* in strains from *Lachnoclostridium, Clostridium,* and other abundant *cutC*-containing genera. The details of *cutC* sequence used for modeling are provided in Supplementary Table 4. Choline ligand was downloaded from ZINC database^57^. The AutoDock (v.4.2.6)^58^ was employed to generate an ensemble of docked conformations for each ligand bound to its target. We used the genetic algorithm for conformational search and performed 100 individual GA runs to generate docked conformations for each ligand.

For each docked conformation, the top-ranked docking pose was optimized in the binding pocket and used as the initial geometry in molecular dynamics. We performed 100 ns molecular dynamics simulation for choline-bound states. All of the molecular dynamics simulations were performed with Amber16^59^.

### Bacterial strains and growth conditions

To verify the in vitro activity of TMA-producing bacteria, *Lachnoclostridium saccharolyticum* WM1 (ATCC 35040) was cultured anaerobically on ATCC medium 1118 at 37 °C. *Clostridium sporogenes* (BNCC 104015) was cultured anaerobically in Trypticase soy agar/broth with defibrinated sheep blood at 37 °C. Two representative intestinal strains, *Prevotella intermedia* (BNCC 352061) and *Bacteroides fragilis* (BNCC336948), were selected as negative controls and were cultured in anaerobically sterilized Trypticase soy agar/broth with defibrinated sheep blood at 37 °C.

For TMA assessment, bacterial cultures were inoculated with choline chloride (Sigma-Aldrich, USA) in sterile tubes and incubated anaerobically (80% N_2_ and 20% CO_2_) at 37 °C (typically OD_600nm_ ~ 1.0). The cultures were centrifuged at 16,200 × *g* for 10 min at 4 °C.

### Quantitative analysis of TMA in bacterial culture medium

Quantification of TMA was performed on a liquid chromatography-20A system coupled to a triple quadrupole mass spectrometer (Shimadzu 8050, Japan) using an internal standard method^15^. Briefly, 10 μL of bacterial culture medium was first mixed with 10 μL of isotope-labeled d9-TMA internal standard (100 μg/mL). Then, 960 μL of n-hexane/n-butyl alcohol (2:1, v/v) and 20 μL of 1 M NaOH were added and vortexed for 3 min to fully extract TMA. After centrifugation (16200 × *g*, 4 °C, 1 min), 500 μL of the organic layer was transferred to a sealed container and acidified with 200 μL of 0.2 N formic acid. Finally, the mixture was vortexed for 3 min and centrifuged at 4 °C for 1 min. An aliquot of 1 μL of the aqueous layer was injected for LC-MS/MS analysis.

LC separation was carried out on a HILIC column (100 × 2.1 mm, 1.7 μm; WATERS). Pure water and acetonitrile both containing 0.1% formic acid were used as mobile phases A and B, respectively, delivered at a flow rate of 0.4 mL/min. An isocratic elution with phase B/phase A (80:20, v/v) was employed and the detection was operated in the positive ion mode. Multiple reaction monitoring (MRM) transitions were performed at *m/z* 60.08 → 44.04 for quantitative detection of TMA, and *m/z* 69 → 49 for d9-TMA. The ESI source parameters were as follows: nebulizing gas flow, 3 L/min; heating gas flow, 10 L/min; drying gas flow, 10 L/min; interface temperature, 300 °C; DL temperature, 250 °C; and heat block temperature, 400 °C. Multiple reaction monitoring transitions were performed at *m/z* 60.08 → 44.04 for quantitative detection of TMA, and *m/z* 69 → 49 for d9-TMA.

### Animal study

The care and treatment of mice were performed in accordance with the Provisions and General Recommendation of Chinese Experimental Animals Administration Legislation and the study was approved by the Animal Ethics Committee of China Pharmaceutical University (No. 2021-04-002). All procedures conformed to the European Parliament directive on the protection of animals used for scientific purposes (Directive 2010/63/EU) or the NIH Guide for the Care and Use of Laboratory Animals.

Apolipoprotein E knockout mice (C57BL/6J *ApoE*^−/−^) were housed in a pathogen-free environment at a temperature of 22–24 °C, humidity 40–60%, and a strict 12 h light cycle. After acclimatization for a week, both female and male *ApoE*^−/−^ mice were randomized into 4 groups: normal group, mice fed standard chow and gavaged with the sterile medium; normal + *L. saccharolyticum* group, mice fed standard chow and gavaged live *L. saccharolyticum* at a dose of 5 × 10^8^ CFUs/100 μl; choline group, mice fed chow supplemented with 1.0% choline (Sigma-Aldrich, USA) and gavaged with the sterile medium; and choline + *L. saccharolyticum* group, mice fed standard chow supplemented with 1.0% choline and gavaged with live *L. saccharolyticum* at a dose of 5 × 10^8^ CFUs/100 μl. The mice number was 20 (half male and half female) for each group. The group sizes were selected according to the minimum experimental requirements and natural factors such as fight-related injury.

After 20 weeks, overnight-fasted mice were anesthetized with 4% isoflurane and their blood was collected from the ventriculus dexter for the assay of glucose (Solarbio, BC2505), non-esterified free fatty acids (Solarbio, BC0595), triglycerides, total cholesterol, low-density lipoprotein, and high-density lipoprotein using commercial Kits (Jiancheng Biotechnology Co., Ltd., Nanjing, China). Their stool samples were collected and immediately stored at −80 °C until further analysis. After that, the mice were euthanized by exsanguination following the guidelines of the Institutional Animal Care, the cardiac and aortic tissues were immediately removed, rinsed with ice-cold physiological saline, and stored at −80 °C or fixed in 4% paraformaldehyde until further analysis.

### Histological analysis

Cardiac and aortic tissues were fixed with 4% (*w*/*v*) paraformaldehyde overnight. Then the tissues were embedded in paraffin, cut into 5 μM slices, and stained with hematoxylin and eosin or oil red O. Images were captured using NanoZoomer 2.0 (Hamamatsu, Japan).

### Assessment of atherosclerotic plaque size

Perivascular adipose tissue and adventitious blood vessels were carefully removed and the prepared aorta was stained with Sudan IV solution (0.1% Sudan in 50% aceton) for 6 min, followed by de-staining in 80% ethanol for 5 min. The images were captured with a Canon EOS 80D Digital Camera. Areas stained red were considered atherosclerotic lesions and were quantified using ImageJ software.

### Quantitation of *L. saccharolyticum* abundance in feces

Fecal samples of each mouse were used for metagenomic DNA extraction using TIANamp Stool DNA Kit according to the manufacturer’s instructions. DNA concentration was measured using NanoDrop 2000 spectrophotometer (Biotek, Germany). Quantitative PCR was carried out using the LC480 detection system (Roche Diagnostics, Basel, Switzerland) and HiScript Q RT SuperMix (Vazyme biotech, Nanjing, China). Primers used in this work are listed in Supplementary Table 5.

### Quantification of TMAO in serum

An isotope-labeled internal standard method was employed for the quantitative analysis of TMAO in serum. Targeted detection of TMAO was performed on an Agilent 1290 infinity liquid chromatography system coupled to a triple quadrupole mass spectrometer (Agilent 6470, USA) operated in the positive ion mode. An aliquot of 10 μL serum was precipitated by adding 980 μL of acetonitrile/ water/formic acid (94:5:1, v/v/v) solution and 10 μL of internal standard working solution (2 μg/ml of d9-TMAO), followed by vortex-mixing for 30 s. Precipitated protein was subsequently removed by centrifugation at a speed of 16200 × *g* at 4 °C for 10 min. Then, 5 μL of supernatant was analyzed by ultra-performance liquid chromatography-tandem mass spectrometry (UPLC-MS/MS).

Chromatographic evaluation was achieved on an ACQUITY UPLC^®^ BEH HILIC column (2.1 × 100 mm, 1.7 μm) maintained at 40 °C. The mobile phase consisted of (A) 10 mM ammonium acetate aqueous solution and (B) 10 mM ammonium acetate water/acetonitrile (1:9) solution delivered at a flow rate of 0.4 mL/min. The gradient elution program was 5–80% B at 0–7 min, 80–100% B at 7–12 min, 100% B at 12–13 min, and then back to initial conditions, with 2 min for equilibration. Targeted detection of TMAO was performed on an Agilent 1290 infinity LC system coupled to a triple quadrupole mass spectrometer (Agilent, LC-MS/MS 6470) operating in the positive ion mode. Multiple reaction monitoring (MRM) mode was performed and the ion transitions monitored were *m/z* 76 → 58 for TMAO and 85 → 66 for d9-TMAO. The detailed MS parameters were set as follows: fragmental voltage, 100 V; capillary voltage, 3500 V; nebulizer gas, 35 psig; drying gas flow rate, 10 L/min; drying gas temperature, 300 °C.

### Statistical analysis

Statistical differences between the two groups were calculated using the Wilcoxon rank-sum test. The multivariable-adjusted analysis using linear regression model was performed to evaluate the R.A. of TMA-lyase against other confounding factors. The Kruskal–Wallis test was for multi-group comparison followed by Holm–Bonferroni correction. The differences among groups in animal experiments were analyzed by single-factor ANOVA with Tukey HSD test. All analyses were performed using R 3.6.1, and *p* < 0.05 were considered statistically significant.

## Supplementary information

Supplementary Information

reporting-summary

## Data Availability

All public metagenomic data used in this manuscript were provided their web links or the accession codes in “Methods” section.

## Code availability

All code used for statistical analysis is available from the corresponding author on reasonable request.
