# Author Correction: Integrated metagenomics identifies a crucial role for trimethylamine-producing *Lachnoclostridium* in promoting atherosclerosis

**DOI:** 10.1038/s41522-022-00303-1

**Published:** 2022-05-09

**Authors:** Yuan-Yuan Cai, Feng-Qing Huang, Xingzhen Lao, Yawen Lu, Xuejiao Gao, Raphael N. Alolga, Kunpeng Yin, Xingchen Zhou, Yun Wang, Baolin Liu, Jing Shang, Lian-Wen Qi, Jing Li

**Affiliations:** 1 School of Life Science and Technology, China Pharmaceutical University, Nanjing, 210009 China; 2 State Key Laboratory of Natural Medicines, School of traditional Chinese Pharmacy, China Pharmaceutical University, Nanjing, 210009 China; 3 Affiliated Hospital of Integrated Traditional Chinese and Western Medicine, Nanjing University of Chinese Medicine, Nanjing, 210028 China; 4 Key Laboratory of Drug Quality Control and Pharmacovigilance (China Pharmaceutical University), Ministry of Education, Nanjing, 210009 China

**Keywords:** Applied microbiology, Clinical microbiology

Correction to: *npj Biofilms and Microbiomes* 10.1038/s41522-022-00273-4, published online 10 March 2022

In the original version of this Article, there were errors in Figures 4 and 5. In Fig. 4 the panel labels were ordered incorrectly. In Fig. 5e the same image was used for both the C and C+L groups. This has now been corrected in the PDF and HTML versions of the Article.

