# Supplementary material for: Integrated metagenomics identifies a crucial role for trimethylamine-producing Lachnoclostridium in promoting atherosclerosis
Source: NPJ Biofilms Microbiomes. 2022 Mar 10;8:11. doi: 10.1038/s41522-022-00273-4 (PMC8913745; doi:10.1038/s41522-022-00273-4)
Supplement: Supplementary file 1 — Supplementary Information [file 41522_2022_273_MOESM1_ESM.pdf]

Supplementary Material

Integrated metagenomics identifies a crucial role for trimethylamine-producing *Lachnoclostridium* in promoting atherosclerosis

Yuan-Yuan Cai [ab#], Feng-Qing Huang [b#], Xingzhen Lao [a], Yawen Lu [b], Xuejiao Gao [c], Raphael N. Alolga [b], Kunpeng Yin [b], Xingchen Zhou[a], Yun Wang [a], Baolin Liu [b], Jing Shang [b*], Lian-Wen Qi [b*], Jing Li [abd*]

[a]*School of Life Science and Technology, China Pharmaceutical University, Nanjing 210009, China;* [b] *State Key Laboratory of Natural Medicines, School of traditional Chinese Pharmacy, China Pharmaceutical University, Nanjing 210009, China;* [c] *Affiliated Hospital of Integrated Traditional Chinese and Western Medicine, Nanjing University of Chinese Medicine, Nanjing 210028, China;* [d] *Key Laboratory of Drug Quality Control and Pharmacovigilance (China Pharmaceutical University)，Ministry of Education，Nanjing 210009, China.*

*corresponding author: Jing Li, e-mail: lijing@cpu.edu.cn; Lian-Wen Qi, e-mail: Qilw@cpu.edu.cn; or Jing Shang, e-mail: shangjing21cn@163.com.

[#]These authors contributed equally to this work.

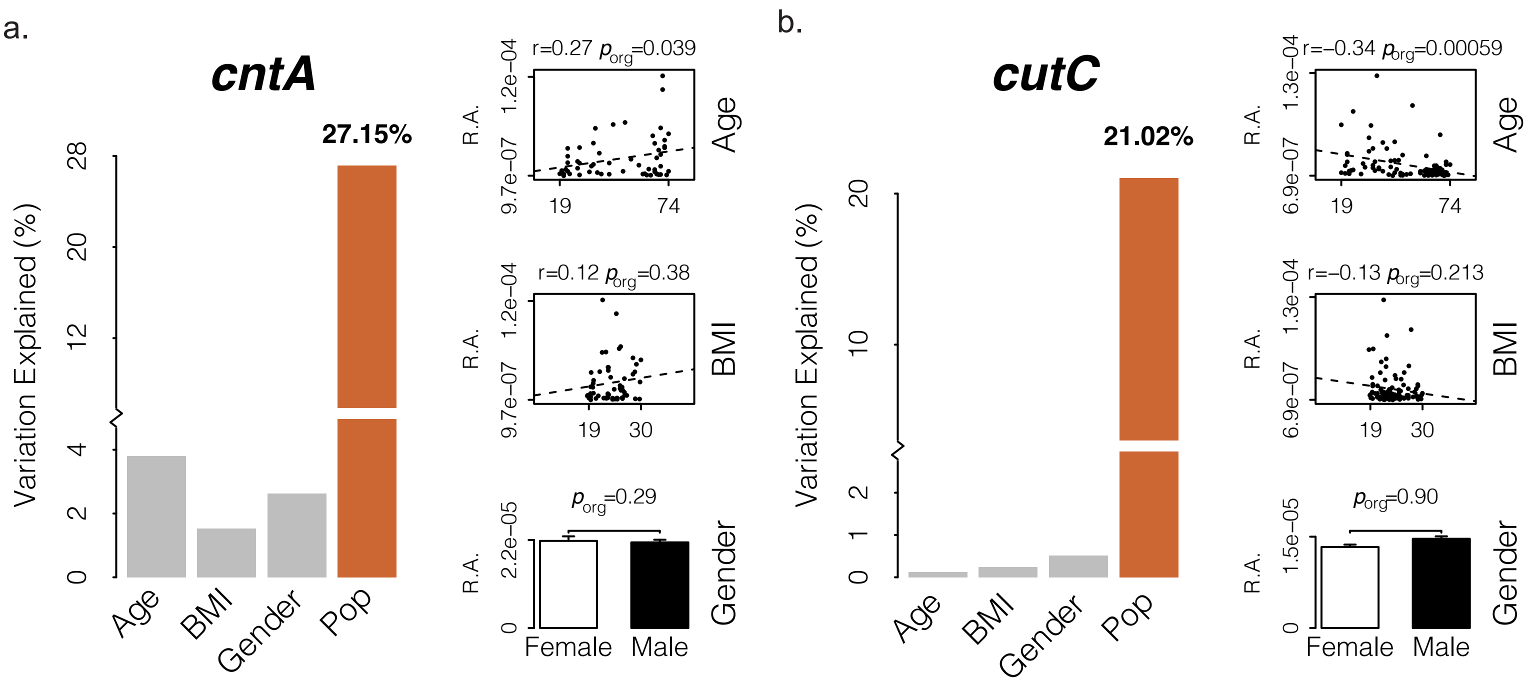
a.
cntA
27.15%
Variation Explained (%)
0
2
4
12
20
28
Age
BMI
Gender
Pop
r=0.27 p_{org}=0.039
R.A.
9.7e−07
1.2e−04
19
74
Age
r=0.12 p_{org}=0.38
R.A.
9.7e−07
1.2e−04
19
30
BMI
p_{org}=0.29
R.A.
0
2.2e−05
Female
Male
Gender
b.
cutC
21.02%
Variation Explained (%)
0
1
2
10
20
Age
BMI
Gender
Pop
r=−0.34 p_{org}=0.00059
R.A.
6.9e−07
1.3e−04
19
74
Age
r=−0.13 p_{org}=0.213
R.A.
6.9e−07
1.3e−04
19
30
BMI
p_{org}=0.90
R.A.
0
1.5e−05
Female
Male
Gender

Figure 1. Multivariate analysis of covariance of *cntA* (**a**) and *cutC* (**b**) based on public metagenomes. Barplots show the sequence relative abundances against binary variables (i.e. gender), Error bars denote the means ± s.e.m. p_{org} indicates the significance conducted by Mann-Whitney-Wilcoxon test; scatterplots indicate the pearson correlation between sequence relative abundances and continuous variables (i.e. age and BMI), p_{org} indicates the correlation significance.

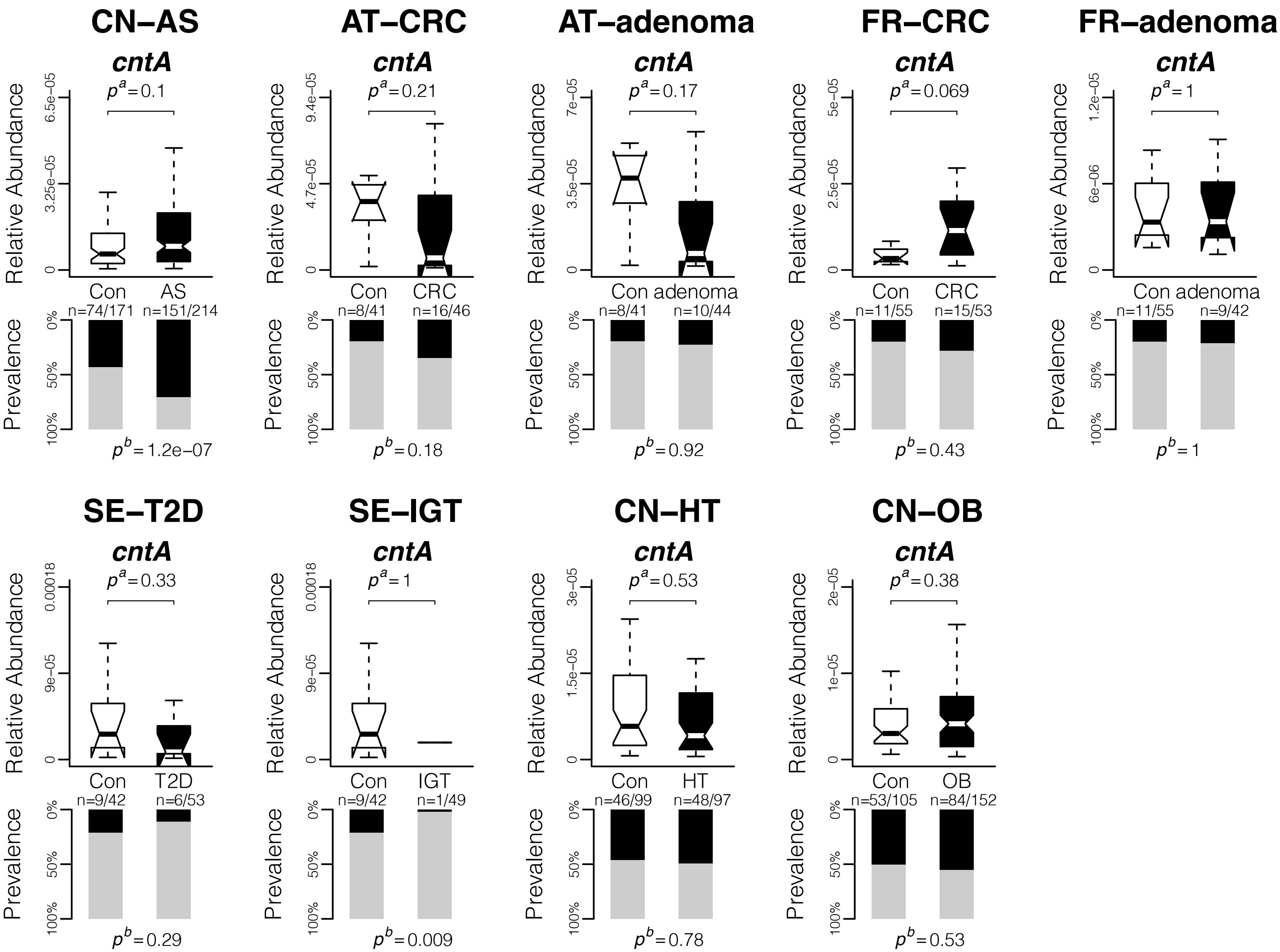

CN–AS
cntA
pa=0.1
Con AS
n=74/171 n=151/214
pb=1.2e–07
AT–CRC
cntA
pa=0.21
Con CRC
n=8/41 n=16/46
pb=0.18
AT–adenoma
cntA
pa=0.17
Con adenoma
n=8/41 n=10/44
pb=0.92
FR–CRC
cntA
pa=0.069
Con CRC
n=11/55 n=15/53
pb=0.43
FR–adenoma
cntA
pa=1
Con adenoma
n=11/55 n=9/42
pb=1
SE–T2D
cntA
pa=0.33
Con T2D
n=9/42 n=6/53
pb=0.29
SE–IGT
cntA
pa=1
Con IGT
n=9/42 n=1/49
pb=0.009
CN–HT
cntA
pa=0.53
Con HT
n=46/99 n=48/97
pb=0.78
CN–OB
cntA
pa=0.38
Con OB
n=53/105 n=84/152
pb=0.53
Relative Abundance
Prevalence
0% 50% 100%

Figure 2. Relative abundance distribution of *cntA* in multiple case- control cohorts in the upper panel. Significant differences were conducted by Mann-Whitney-Wilcoxon test (p^a). The bounds denote the interquartile range between the first and third quartiles and the center line denotes the median. The whiskers denote the lowest and highest value. Bar plots in the lower panel show the prevalence difference in multiple case-control cohorts. Significant differences were conducted by Chi-squared test (p^b). CN-AS: atherosclerosis from China; AT-CRC: colorectal cancer from Austria; AT-adenoma: adenoma from Austria; FR-CRC: colorectal cancer from France; FR-adenoma: adenoma from France; SE-T2D: type 2 diabetes from Sweden; SE-IGT: impaired glucose tolerance from Sweden; CN-HT: hypertension from China; CN-OB: obesity from China.

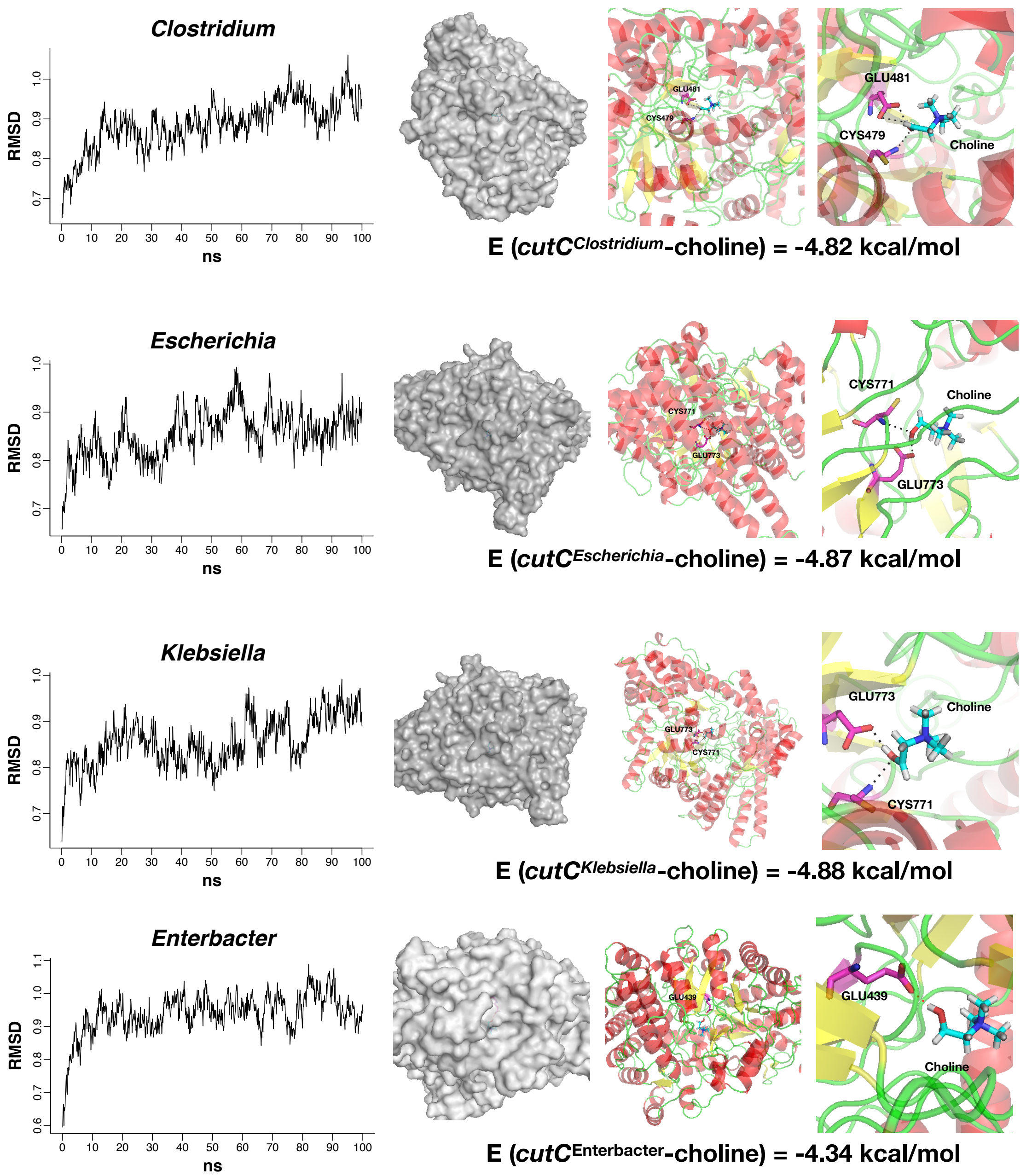
Clostridium
RMSD
ns
GLU481
CYS479
Choline
E (cutC^Clostridium-choline) = -4.82 kcal/mol
Escherichia
RMSD
ns
CYS771
GLU773
Choline
E (cutC^Escherichia-choline) = -4.87 kcal/mol
Klebsiella
RMSD
ns
GLU773
CYS771
Choline
E (cutC^Klebsiella-choline) = -4.88 kcal/mol
Enterbacter
RMSD
ns
GLU439
Choline
E (cutC^Enterbacter-choline) = -4.34 kcal/mol

Figure 3. Molecular docking of the other 4 most abundant candidate TMA-producing genera and choline. The binding pockets are shown in the left panel of the figure, the active binding site on the macromolecular protein has been marked in bold, and the intermolecular hydrogen bond is shown by the black dotted line. Amber16 was used to calculate the dynamic changes within 100 ns of the combined compound, and its RMSD is shown in the right column.

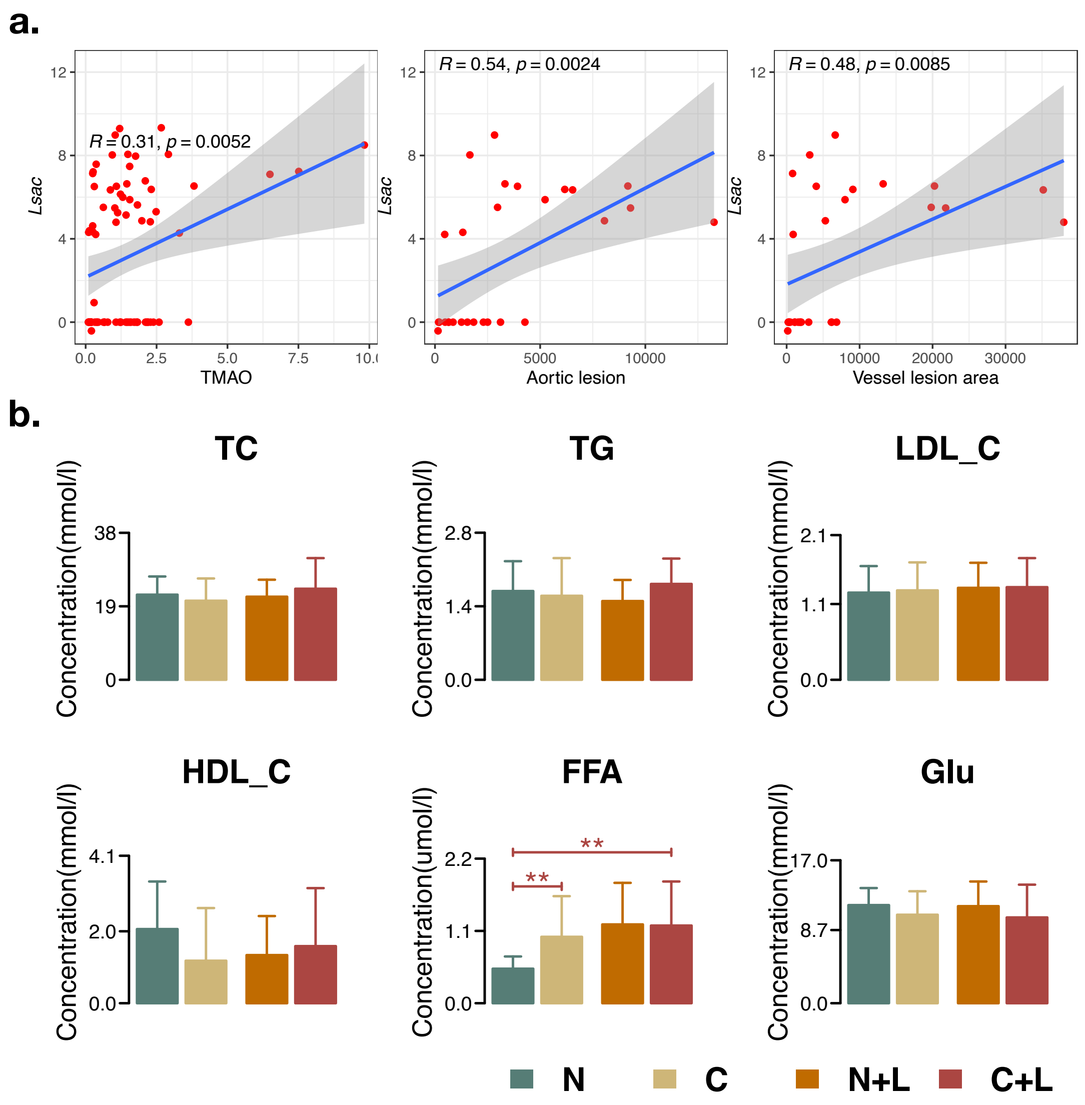
a.
R = 0.31, p = 0.0052
R = 0.54, p = 0.0024
R = 0.48, p = 0.0085
Lsac
TMAO
Aortic lesion
Vessel lesion area
b.
TC
TG
LDL_C
HDL_C
FFA
Glu
Concentration(mmol/l)
Concentration(umol/l)
N
C
N+L
C+L

Figure 4. The effect of *L. saccharolyticum* on atherosclerosis in *ApoE*$^{-/-}$ mice. (**a**) Pearson correlation analysis between the level of *L. saccharolyticum* in feces and TMAO level in serum, aortic lesion and vessel lesion area. (**b**) Barplots show the concentration of glucose (Glu), non-esterified free fatty acids (FFA), triglycerides (TG), total cholesterol (TC), low-density lipoprotein (LDL_C), and high-density lipoprotein (HDL_C). Error bars denote the means ± SEM. * $p < 0.05$, ** $p < 0.01$, *** $p < 0.001$, the differences among groups were analyzed by single-factor ANOVA corrected with Tukey HSD test. N: normal group, mice fed with standard chow control diet and gavaged with the sterile medium; N+L: normal + *L.saccharolyticum* group, mice fed standard chow and gavaged live *L.saccharolyticum* at a dose of 5 × 108 CFUs/100 µl; C: choline group, mice fed with standard chow supplemented with 1.0% choline (Sigma-Aldrich, USA) and gavaged with the sterile medium; C+L: choline + *L.saccharolyticum* group, mice fed with standard chow supplemented with 1.0% choline and gavaged with live *L.saccharolyticum* at a dose of 5 × 108 CFUs/100 µl.

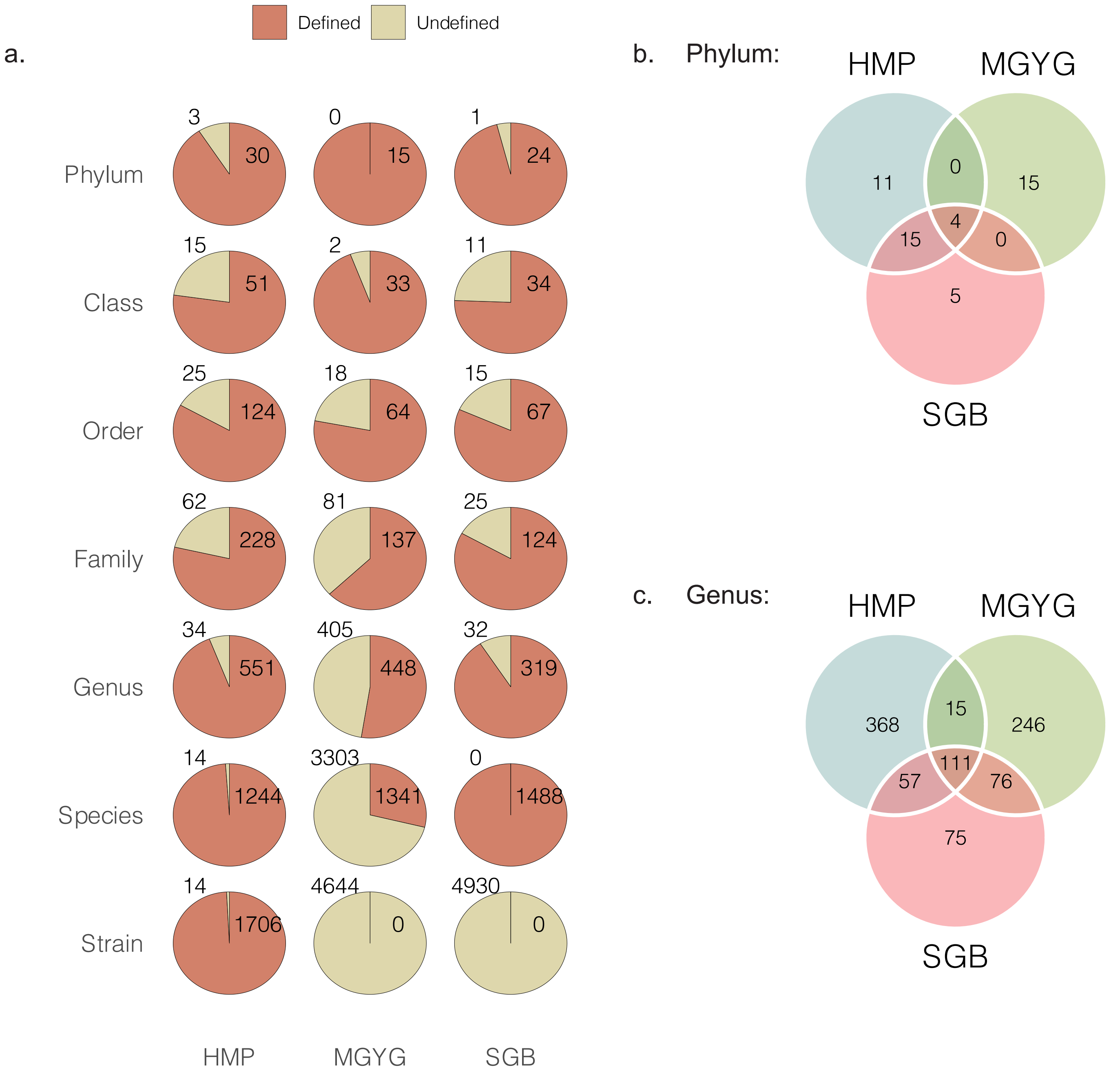
Defined
Undefined
a.
Phylum
Class
Order
Family
Genus
Species
Strain
HMP
MGYG
SGB
3
30
0
15
1
24
15
51
2
33
11
34
25
124
18
64
15
67
62
228
81
137
25
124
34
551
405
448
32
319
14
1244
3303
1341
0
1488
14
1706
4644
0
4930
0
b. Phylum:
HMP
MGYG
SGB
11
0
15
4
15
0
5
c. Genus:
HMP
MGYG
SGB
368
15
246
111
57
76
75

Figure 5. Taxonomic information of three reference genome resources. (**a**) Pie charts of the defined and undefined numbers of biological taxonomy. (**b**) Venn diagram analysis of phyla in the three resources. (**c**) Venn diagram analysis of the genera in the three resources.

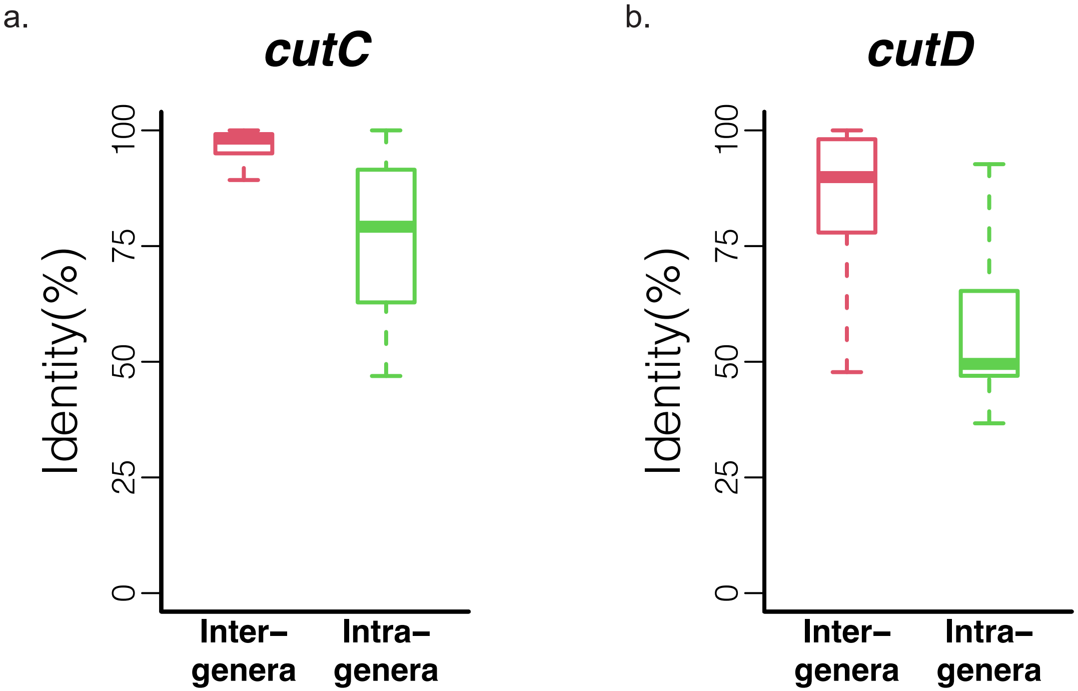
a.
cutC
b.
cutD
Identity(%)
100
75
50
25
0
Inter–
genera
Intra–
genera

Figure 6. Inter- and intra-genera sequence identities of *cntA* (**a**) and *cutC* (**b**). The bounds denote the interquartile range between the first and third quartiles and the center line denotes the median. The whiskers denote the lowest and highest value.

Supplementary Table 1. Taxaonmic information of reference TMA-lyase.

Resource	Phylum	Class	Order	Family	Genus	Species	Strain	Type
HMP	Proteobacteria	Gammaproteobacteria	Enterobacteriales	Enterobacteriaceae	Shigella	Shigella flexneri	Shigella flexneri 2a str. 301	cntA
HMP	Proteobacteria	Gammaproteobacteria	Pseudomonadales	Moraxellaceae	Acinetobacter	Acinetobacter baumannii	Acinetobacter baumannii AB307-0294	cntA
HMP	Proteobacteria	Gammaproteobacteria	Pseudomonadales	Moraxellaceae	Acinetobacter	Acinetobacter baumannii	Acinetobacter baumannii AB0057	cntA
HMP	Proteobacteria	Gammaproteobacteria	Pseudomonadales	Moraxellaceae	Acinetobacter	Acinetobacter baumannii	Acinetobacter baumannii AYE	cntA
HMP	Proteobacteria	Betaproteobacteria	Burkholderiales	Burkholderiaceae	Cupriavidus	Cupriavidus necator	Ralstonia eutropha H16	cntA
HMP	Proteobacteria	Gammaproteobacteria	Pseudomonadales	Pseudomonadaceae	Pseudomonas	Pseudomonas entomophila	Pseudomonas entomophila L48	cntA
HMP	Proteobacteria	Betaproteobacteria	Burkholderiales	Burkholderiaceae	Cupriavidus	Cupriavidus pinatubonensis	Ralstonia eutropha JMP134	cntA
HMP	Proteobacteria	Gammaproteobacteria	Enterobacteriales	Enterobacteriaceae	Citrobacter	Citrobacter sp. 30_2	Citrobacter sp. 30_2	cntA
HMP	Proteobacteria	Gammaproteobacteria	Pseudomonadales	Moraxellaceae	Acinetobacter	Acinetobacter baumannii	Acinetobacter baumannii ATCC 19606 = CIP 70.34 = JCM 6841	cntA
HMP	Proteobacteria	Gammaproteobacteria	Pseudomonadales	Moraxellaceae	Acinetobacter	Acinetobacter nosocomialis	Acinetobacter sp. RUH2624	cntA
HMP	Proteobacteria	Gammaproteobacteria	Enterobacteriales	Enterobacteriaceae	Yersinia	Yersinia pestis	Yersinia pestis biovar Orientalis str. IP275	cntA
HMP	Proteobacteria	Gammaproteobacteria	Enterobacteriales	Enterobacteriaceae	Yersinia	Yersinia pestis	Yersinia pestis biovar Antiqua str. E1979001	cntA
HMP	Proteobacteria	Gammaproteobacteria	Enterobacteriales	Enterobacteriaceae	Escherichia	Escherichia coli	Escherichia coli SE11	cntA
HMP	Proteobacteria	Gammaproteobacteria	Enterobacteriales	Enterobacteriaceae	Providencia	Providencia stuartii	Providencia stuartii ATCC 25827	cntA
HMP	Proteobacteria	Gammaproteobacteria	Enterobacteriales	Enterobacteriaceae	Escherichia	Escherichia coli	Escherichia coli O157:H7 str. EC4024	cntA
HMP	Proteobacteria	Gammaproteobacteria	Enterobacteriales	Enterobacteriaceae	Escherichia	Escherichia albertii	Escherichia albertii TW07627	cntA
HMP	Proteobacteria	Gammaproteobacteria	Enterobacteriales	Enterobacteriaceae	Escherichia	Escherichia coli	Escherichia coli D9	cntA
HMP	Proteobacteria	Gammaproteobacteria	Enterobacteriales	Enterobacteriaceae	Escherichia	Escherichia sp. 4_1_40B	Escherichia sp. 4_1_40B	cntA
HMP	Proteobacteria	Gammaproteobacteria	Enterobacteriales	Enterobacteriaceae	Yersinia	Yersinia pestis	Yersinia pestis Pestoides A	cntA
HMP	Proteobacteria	Gammaproteobacteria	Enterobacteriales	Enterobacteriaceae	Klebsiella	Klebsiella pneumoniae	Klebsiella pneumoniae subsp. rhinoscleromatis ATCC 13884	cntA
HMP	Proteobacteria	Betaproteobacteria	Burkholderiales	Alcaligenaceae	Achromobacter	Achromobacter piechaudii	Achromobacter piechaudii ATCC 43553	cntA
HMP	Proteobacteria	Gammaproteobacteria	Enterobacteriales	Enterobacteriaceae	Yersinia	Yersinia pestis	Yersinia pestis biovar Mediaevalis str. K1973002	cntA
HMP	Proteobacteria	Gammaproteobacteria	Enterobacteriales	Enterobacteriaceae	Escherichia	Escherichia coli	Escherichia coli OP50	cntA
HMP	Proteobacteria	Gammaproteobacteria	Enterobacteriales	Enterobacteriaceae	Yersinia	Yersinia pestis	Yersinia pestis KIM D27	cntA
HMP	Proteobacteria	Gammaproteobacteria	Pseudomonadales	Moraxellaceae	Acinetobacter	Acinetobacter baumannii	Acinetobacter baumannii AB056	cntA
HMP	Proteobacteria	Gammaproteobacteria	Pseudomonadales	Moraxellaceae	Acinetobacter	Acinetobacter baumannii	Acinetobacter baumannii AB058	cntA
HMP	Proteobacteria	Gammaproteobacteria	Pseudomonadales	Moraxellaceae	Acinetobacter	Acinetobacter baumannii	Acinetobacter baumannii AB059	cntA
HMP	Proteobacteria	Gammaproteobacteria	Enterobacteriales	Enterobacteriaceae	Escherichia	Escherichia coli	Escherichia coli MS 124-1	cntA
HMP	Proteobacteria	Gammaproteobacteria	Enterobacteriales	Enterobacteriaceae	Escherichia	Escherichia coli	Escherichia coli MS 119-7	cntA
HMP	Proteobacteria	Gammaproteobacteria	Enterobacteriales	Enterobacteriaceae	Escherichia	Escherichia coli	Escherichia coli MS 107-1	cntA
HMP	Proteobacteria	Gammaproteobacteria	Pseudomonadales	Moraxellaceae	Acinetobacter	Acinetobacter baumannii	Acinetobacter baumannii 6013150	cntA
HMP	Proteobacteria	Gammaproteobacteria	Pseudomonadales	Moraxellaceae	Acinetobacter	Acinetobacter baumannii	Acinetobacter baumannii 6013113	cntA
HMP	Proteobacteria	Gammaproteobacteria	Pseudomonadales	Moraxellaceae	Acinetobacter	Acinetobacter baumannii	Acinetobacter baumannii 6014059	cntA
HMP	Proteobacteria	Gammaproteobacteria	Enterobacteriales	Enterobacteriaceae	Providencia	Providencia rettgeri	Providencia rettgeri DSM 1131	cntA
HMP	Proteobacteria	Gammaproteobacteria	Enterobacteriales	Enterobacteriaceae	Citrobacter	Citrobacter youngae	Citrobacter youngae ATCC 29220	cntA
HMP	Proteobacteria	Gammaproteobacteria	Enterobacteriales	Enterobacteriaceae	Klebsiella	Klebsiella sp. 1_1_55	Klebsiella sp. 1_1_55	cntA
HMP	Proteobacteria	Gammaproteobacteria	Enterobacteriales	Enterobacteriaceae	Escherichia	Escherichia coli	Escherichia coli B088	cntA
HMP	Proteobacteria	Gammaproteobacteria	Enterobacteriales	Enterobacteriaceae	Escherichia	Escherichia coli	Escherichia coli B185	cntA
HMP	Proteobacteria	Gammaproteobacteria	Enterobacteriales	Enterobacteriaceae	Escherichia	Escherichia coli	Escherichia coli FVEC1412	cntA
HMP	Proteobacteria	Gammaproteobacteria	Enterobacteriales	Enterobacteriaceae	Escherichia	Escherichia coli	Escherichia coli B354	cntA
HMP	Proteobacteria	Gammaproteobacteria	Pseudomonadales	Moraxellaceae	Acinetobacter	Acinetobacter pittii	Acinetobacter sp. SH024	cntA
HMP	Proteobacteria	Gammaproteobacteria	Enterobacteriales	Enterobacteriaceae	Escherichia	Escherichia coli	Escherichia coli MS 198-1	cntA
HMP	Proteobacteria	Gammaproteobacteria	Enterobacteriales	Enterobacteriaceae	Escherichia	Escherichia coli	Escherichia coli MS 84-1	cntA
HMP	Proteobacteria	Gammaproteobacteria	Enterobacteriales	Enterobacteriaceae	Escherichia	Escherichia coli	Escherichia coli MS 115-1	cntA
HMP	Proteobacteria	Gammaproteobacteria	Enterobacteriales	Enterobacteriaceae	Escherichia	Escherichia coli	Escherichia coli MS 182-1	cntA
HMP	Proteobacteria	Gammaproteobacteria	Enterobacteriales	Enterobacteriaceae	Escherichia	Escherichia coli	Escherichia coli MS 146-1	cntA
HMP	Proteobacteria	Gammaproteobacteria	Enterobacteriales	Enterobacteriaceae	Escherichia	Escherichia coli	Escherichia coli MS 69-1	cntA
HMP	Proteobacteria	Gammaproteobacteria	Enterobacteriales	Enterobacteriaceae	Escherichia	Escherichia coli	Escherichia coli MS 187-1	cntA
HMP	Proteobacteria	Gammaproteobacteria	Enterobacteriales	Enterobacteriaceae	Escherichia	Escherichia coli	Escherichia coli MS 21-1	cntA
HMP	Proteobacteria	Gammaproteobacteria	Enterobacteriales	Enterobacteriaceae	Escherichia	Escherichia coli	Escherichia coli MS 78-1	cntA
HMP	Proteobacteria	Gammaproteobacteria	Enterobacteriales	Enterobacteriaceae	Escherichia	Escherichia coli	Escherichia coli MS 116-1	cntA
HMP	Proteobacteria	Gammaproteobacteria	Enterobacteriales	Enterobacteriaceae	Escherichia	Escherichia coli	Escherichia coli MS 175-1	cntA
HMP	Proteobacteria	Gammaproteobacteria	Enterobacteriales	Enterobacteriaceae	Escherichia	Escherichia coli	Escherichia coli MS 196-1	cntA
HMP	Proteobacteria	Gammaproteobacteria	Enterobacteriales	Enterobacteriaceae	Escherichia	Escherichia coli	Escherichia coli FVEC1302	cntA
HMP	Proteobacteria	Gammaproteobacteria	Enterobacteriales	Enterobacteriaceae	Escherichia	Escherichia coli	Escherichia coli str. K-12 substr. MG1655	cntA
HMP	Proteobacteria	Gammaproteobacteria	Enterobacteriales	Enterobacteriaceae	Xenorhabdus	Xenorhabdus nematophila	Xenorhabdus nematophila ATCC 19061	cntA
HMP	Proteobacteria	Gammaproteobacteria	Enterobacteriales	Enterobacteriaceae	Yersinia	Yersinia pestis	Yersinia pestis Z176003	cntA
HMP	Proteobacteria	Betaproteobacteria	Burkholderiales	Burkholderiaceae	Burkholderia	Burkholderia multivorans	Burkholderia multivorans ATCC 17616	cntA
HMP	Proteobacteria	Gammaproteobacteria	Pseudomonadales	Moraxellaceae	Acinetobacter	Acinetobacter oleivorans	Acinetobacter oleivorans DR1	cntA
HMP	Proteobacteria	Gammaproteobacteria	Enterobacteriales	Enterobacteriaceae	Escherichia	Escherichia coli	Escherichia coli O55:H7 str. CB9615	cntA
HMP	Proteobacteria	Gammaproteobacteria	Enterobacteriales	Enterobacteriaceae	Klebsiella	Klebsiella variicola	Klebsiella variicola At-22	cntA
HMP	Proteobacteria	Gammaproteobacteria	Enterobacteriales	Enterobacteriaceae	Escherichia	Escherichia coli	Escherichia coli O26:H11 str. 11368	cntA
HMP	Proteobacteria	Gammaproteobacteria	Enterobacteriales	Enterobacteriaceae	Shigella	Shigella flexneri	Shigella flexneri 2002017	cntA
HMP	Proteobacteria	Gammaproteobacteria	Enterobacteriales	Enterobacteriaceae	Escherichia	Escherichia coli	Escherichia coli 042	cntA
HMP	Proteobacteria	Gammaproteobacteria	Pseudomonadales	Moraxellaceae	Acinetobacter	Acinetobacter baumannii	Acinetobacter baumannii ATCC 17978	cntA
HMP	Proteobacteria	Gammaproteobacteria	Enterobacteriales	Enterobacteriaceae	Shigella	Shigella boydii	Shigella boydii CDC 3083-94	cntA
HMP	Proteobacteria	Gammaproteobacteria	Enterobacteriales	Enterobacteriaceae	Yersinia	Yersinia pseudotuberculosis	Yersinia pseudotuberculosis IP 32953	cntA
HMP	Proteobacteria	Gammaproteobacteria	Pseudomonadales	Pseudomonadaceae	Pseudomonas	Pseudomonas putida	Pseudomonas putida GB-1	cntA
HMP	Proteobacteria	Gammaproteobacteria	Enterobacteriales	Enterobacteriaceae	Shigella	Shigella sonnei	Shigella sonnei Ss046	cntA
HMP	Proteobacteria	Gammaproteobacteria	Enterobacteriales	Enterobacteriaceae	Yersinia	Yersinia pestis	Yersinia pestis biovar Microtus str. 91001	cntA
HMP	Proteobacteria	Gammaproteobacteria	Enterobacteriales	Enterobacteriaceae	Shigella	Shigella flexneri	Shigella flexneri 2a str. 301	cntB
HMP	Proteobacteria	Gammaproteobacteria	Pseudomonadales	Moraxellaceae	Acinetobacter	Acinetobacter baumannii	Acinetobacter baumannii AB307-0294	cntB
HMP	Proteobacteria	Gammaproteobacteria	Pseudomonadales	Moraxellaceae	Acinetobacter	Acinetobacter baumannii	Acinetobacter baumannii AB0057	cntB
HMP	Proteobacteria	Gammaproteobacteria	Pseudomonadales	Moraxellaceae	Acinetobacter	Acinetobacter baumannii	Acinetobacter baumannii AYE	cntB
HMP	Proteobacteria	Betaproteobacteria	Burkholderiales	Burkholderiaceae	Cupriavidus	Cupriavidus necator	Ralstonia eutropha H16	cntB
HMP	Proteobacteria	Gammaproteobacteria	Pseudomonadales	Pseudomonadaceae	Pseudomonas	Pseudomonas entomophila	Pseudomonas entomophila L48	cntB
HMP	Proteobacteria	Betaproteobacteria	Burkholderiales	Burkholderiaceae	Cupriavidus	Cupriavidus pinatubonensis	Ralstonia eutropha JMP134	cntB
HMP	Proteobacteria	Gammaproteobacteria	Thiotrichales	Piscirickettsiaceae	Methylophaga	Methylophaga thiooxydans	Methylophaga thiooxydans DMS010	cntB
HMP	Proteobacteria	Alphaproteobacteria	Rhodobacterales	Rhodobacteraceae	unclassified Rhodobacteraceae	Rhodobacteraceae bacterium KLH11	Rhodobacteraceae bacterium KLH11	cntB
HMP	Proteobacteria	Gammaproteobacteria	Enterobacteriales	Enterobacteriaceae	Citrobacter	Citrobacter sp. 30_2	Citrobacter sp. 30_2	cntB
HMP	Proteobacteria	Gammaproteobacteria	Pseudomonadales	Moraxellaceae	Acinetobacter	Acinetobacter baumannii	Acinetobacter baumannii ATCC 19606 = CIP 70.34 = JCM 6841	cntB
HMP	Proteobacteria	Gammaproteobacteria	Pseudomonadales	Moraxellaceae	Acinetobacter	Acinetobacter nosocomialis	Acinetobacter sp. RUH2624	cntB
HMP	Proteobacteria	Gammaproteobacteria	Enterobacteriales	Enterobacteriaceae	Yersinia	Yersinia pestis	Yersinia pestis biovar Orientalis str. IP275	cntB
HMP	Proteobacteria	Gammaproteobacteria	Enterobacteriales	Enterobacteriaceae	Yersinia	Yersinia pestis	Yersinia pestis biovar Antiqua str. E1979001	cntB
HMP	Proteobacteria	Gammaproteobacteria	Enterobacteriales	Enterobacteriaceae	Escherichia	Escherichia coli	Escherichia coli SE11	cntB
HMP	Proteobacteria	Gammaproteobacteria	Enterobacteriales	Enterobacteriaceae	Providencia	Providencia stuartii	Providencia stuartii ATCC 25827	cntB
HMP	Proteobacteria	Gammaproteobacteria	Enterobacteriales	Enterobacteriaceae	Escherichia	Escherichia coli	Escherichia coli O157:H7 str. EC4024	cntB
HMP	Proteobacteria	Gammaproteobacteria	Enterobacteriales	Enterobacteriaceae	Escherichia	Escherichia albertii	Escherichia albertii TW07627	cntB
HMP	Proteobacteria	Gammaproteobacteria	Enterobacteriales	Enterobacteriaceae	Escherichia	Escherichia coli	Escherichia coli D9	cntB
HMP	Proteobacteria	Gammaproteobacteria	Enterobacteriales	Enterobacteriaceae	Escherichia	Escherichia sp. 4_1_40B	Escherichia sp. 4_1_40B	cntB
HMP	Proteobacteria	Gammaproteobacteria	Enterobacteriales	Enterobacteriaceae	Yersinia	Yersinia pestis	Yersinia pestis Pestoides A	cntB
HMP	Proteobacteria	Gammaproteobacteria	Enterobacteriales	Enterobacteriaceae	Klebsiella	Klebsiella pneumoniae	Klebsiella pneumoniae subsp. rhinoscleromatis ATCC 13884	cntB
HMP	Proteobacteria	Betaproteobacteria	Burkholderiales	Alcaligenaceae	Achromobacter	Achromobacter piechaudii	Achromobacter piechaudii ATCC 43553	cntB
HMP	Proteobacteria	Gammaproteobacteria	Enterobacteriales	Enterobacteriaceae	Yersinia	Yersinia pestis	Yersinia pestis biovar Mediaevalis str. K1973002	cntB
HMP	Proteobacteria	Gammaproteobacteria	Enterobacteriales	Enterobacteriaceae	Escherichia	Escherichia coli	Escherichia coli OP50	cntB
HMP	Proteobacteria	Gammaproteobacteria	Enterobacteriales	Enterobacteriaceae	Yersinia	Yersinia pestis	Yersinia pestis KIM D27	cntB
HMP	Proteobacteria	Gammaproteobacteria	Pseudomonadales	Moraxellaceae	Acinetobacter	Acinetobacter baumannii	Acinetobacter baumannii AB056	cntB
HMP	Proteobacteria	Gammaproteobacteria	Pseudomonadales	Moraxellaceae	Acinetobacter	Acinetobacter baumannii	Acinetobacter baumannii AB058	cntB
HMP	Proteobacteria	Gammaproteobacteria	Pseudomonadales	Moraxellaceae	Acinetobacter	Acinetobacter baumannii	Acinetobacter baumannii AB059	cntB
HMP	Proteobacteria	Gammaproteobacteria	Enterobacteriales	Enterobacteriaceae	Escherichia	Escherichia coli	Escherichia coli MS 124-1	cntB
HMP	Proteobacteria	Gammaproteobacteria	Enterobacteriales	Enterobacteriaceae	Escherichia	Escherichia coli	Escherichia coli MS 119-7	cntB
HMP	Proteobacteria	Gammaproteobacteria	Enterobacteriales	Enterobacteriaceae	Escherichia	Escherichia coli	Escherichia coli MS 107-1	cntB
HMP	Proteobacteria	Gammaproteobacteria	Pseudomonadales	Moraxellaceae	Acinetobacter	Acinetobacter baumannii	Acinetobacter baumannii 6013150	cntB
HMP	Proteobacteria	Gammaproteobacteria	Pseudomonadales	Moraxellaceae	Acinetobacter	Acinetobacter baumannii	Acinetobacter baumannii 6013113	cntB
HMP	Proteobacteria	Gammaproteobacteria	Pseudomonadales	Moraxellaceae	Acinetobacter	Acinetobacter baumannii	Acinetobacter baumannii 6014059	cntB
HMP	Proteobacteria	Gammaproteobacteria	Enterobacteriales	Enterobacteriaceae	Providencia	Providencia rettgeri	Providencia rettgeri DSM 1131	cntB
HMP	Proteobacteria	Gammaproteobacteria	Enterobacteriales	Enterobacteriaceae	Citrobacter	Citrobacter youngae	Citrobacter youngae ATCC 29220	cntB
HMP	Proteobacteria	Gammaproteobacteria	Enterobacteriales	Enterobacteriaceae	Klebsiella	Klebsiella sp. 1_1_55	Klebsiella sp. 1_1_55	cntB
HMP	Proteobacteria	Gammaproteobacteria	Enterobacteriales	Enterobacteriaceae	Escherichia	Escherichia coli	Escherichia coli B088	cntB
HMP	Proteobacteria	Gammaproteobacteria	Enterobacteriales	Enterobacteriaceae	Escherichia	Escherichia coli	Escherichia coli B185	cntB
HMP	Proteobacteria	Gammaproteobacteria	Enterobacteriales	Enterobacteriaceae	Escherichia	Escherichia coli	Escherichia coli FVEC1412	cntB
HMP	Proteobacteria	Gammaproteobacteria	Enterobacteriales	Enterobacteriaceae	Escherichia	Escherichia coli	Escherichia coli B354	cntB
HMP	Proteobacteria	Gammaproteobacteria	Pseudomonadales	Moraxellaceae	Acinetobacter	Acinetobacter pittii	Acinetobacter sp. SH024	cntB
HMP	Proteobacteria	Gammaproteobacteria	Enterobacteriales	Enterobacteriaceae	Escherichia	Escherichia coli	Escherichia coli MS 198-1	cntB
HMP	Proteobacteria	Gammaproteobacteria	Enterobacteriales	Enterobacteriaceae	Escherichia	Escherichia coli	Escherichia coli MS 84-1	cntB
HMP	Proteobacteria	Gammaproteobacteria	Enterobacteriales	Enterobacteriaceae	Escherichia	Escherichia coli	Escherichia coli MS 115-1	cntB
HMP	Proteobacteria	Gammaproteobacteria	Enterobacteriales	Enterobacteriaceae	Escherichia	Escherichia coli	Escherichia coli MS 182-1	cntB
HMP	Proteobacteria	Gammaproteobacteria	Enterobacteriales	Enterobacteriaceae	Escherichia	Escherichia coli	Escherichia coli MS 146-1	cntB
HMP	Proteobacteria	Gammaproteobacteria	Enterobacteriales	Enterobacteriaceae	Escherichia	Escherichia coli	Escherichia coli MS 69-1	cntB
HMP	Proteobacteria	Gammaproteobacteria	Enterobacteriales	Enterobacteriaceae	Escherichia	Escherichia coli	Escherichia coli MS 187-1	cntB
HMP	Proteobacteria	Gammaproteobacteria	Enterobacteriales	Enterobacteriaceae	Escherichia	Escherichia coli	Escherichia coli MS 21-1	cntB
HMP	Proteobacteria	Gammaproteobacteria	Enterobacteriales	Enterobacteriaceae	Escherichia	Escherichia coli	Escherichia coli MS 78-1	cntB
HMP	Proteobacteria	Gammaproteobacteria	Enterobacteriales	Enterobacteriaceae	Escherichia	Escherichia coli	Escherichia coli MS 116-1	cntB
HMP	Proteobacteria	Gammaproteobacteria	Enterobacteriales	Enterobacteriaceae	Escherichia	Escherichia coli	Escherichia coli MS 175-1	cntB
HMP	Proteobacteria	Gammaproteobacteria	Enterobacteriales	Enterobacteriaceae	Escherichia	Escherichia coli	Escherichia coli MS 196-1	cntB
HMP	Proteobacteria	Gammaproteobacteria	Enterobacteriales	Enterobacteriaceae	Escherichia	Escherichia coli	Escherichia coli FVEC1302	cntB
HMP	Proteobacteria	Gammaproteobacteria	Enterobacteriales	Enterobacteriaceae	Escherichia	Escherichia coli	Escherichia coli str. K-12 substr. MG1655	cntB
HMP	Proteobacteria	Alphaproteobacteria	Rhodobacterales	Rhodobacteraceae	Ruegeria	Ruegeria pomeroyi	Ruegeria pomeroyi DSS-3	cntB
HMP	Proteobacteria	Gammaproteobacteria	Enterobacteriales	Enterobacteriaceae	Xenorhabdus	Xenorhabdus nematophila	Xenorhabdus nematophila ATCC 19061	cntB
HMP	Proteobacteria	Gammaproteobacteria	Enterobacteriales	Enterobacteriaceae	Yersinia	Yersinia pestis	Yersinia pestis Z176003	cntB
HMP	Proteobacteria	Betaproteobacteria	Burkholderiales	Burkholderiaceae	Burkholderia	Burkholderia multivorans	Burkholderia multivorans ATCC 17616	cntB
HMP	Proteobacteria	Alphaproteobacteria	Rhizobiales	Hyphomicrobiaceae	Hyphomicrobium	Hyphomicrobium denitrificans	Hyphomicrobium denitrificans ATCC 51888	cntB
HMP	Proteobacteria	Betaproteobacteria	Methylophilales	Methylophilaceae	Methylotenera	Methylotenera versatilis	Methylotenera versatilis 301	cntB
HMP	Proteobacteria	Gammaproteobacteria	Pseudomonadales	Moraxellaceae	Acinetobacter	Acinetobacter oleivorans	Acinetobacter oleivorans DR1	cntB
HMP	Proteobacteria	Gammaproteobacteria	Enterobacteriales	Enterobacteriaceae	Escherichia	Escherichia coli	Escherichia coli O55:H7 str. CB9615	cntB
HMP	Proteobacteria	Gammaproteobacteria	Pseudomonadales	Moraxellaceae	Acinetobacter	Acinetobacter baumannii	Acinetobacter baumannii SDF	cntB
HMP	Proteobacteria	Gammaproteobacteria	Enterobacteriales	Enterobacteriaceae	Klebsiella	Klebsiella variicola	Klebsiella variicola At-22	cntB
HMP	Proteobacteria	Gammaproteobacteria	Enterobacteriales	Enterobacteriaceae	Escherichia	Escherichia coli	Escherichia coli O26:H11 str. 11368	cntB
HMP	Proteobacteria	Gammaproteobacteria	Enterobacteriales	Enterobacteriaceae	Shigella	Shigella flexneri	Shigella flexneri 2002017	cntB
HMP	Proteobacteria	Gammaproteobacteria	Enterobacteriales	Enterobacteriaceae	Escherichia	Escherichia coli	Escherichia coli 042	cntB
HMP	Proteobacteria	Gammaproteobacteria	Pseudomonadales	Moraxellaceae	Acinetobacter	Acinetobacter baumannii	Acinetobacter baumannii ATCC 17978	cntB
HMP	Proteobacteria	Gammaproteobacteria	Enterobacteriales	Enterobacteriaceae	Shigella	Shigella boydii	Shigella boydii CDC 3083-94	cntB
HMP	Proteobacteria	Gammaproteobacteria	Enterobacteriales	Enterobacteriaceae	Yersinia	Yersinia pseudotuberculosis	Yersinia pseudotuberculosis IP 32953	cntB
HMP	Proteobacteria	Gammaproteobacteria	Pseudomonadales	Pseudomonadaceae	Pseudomonas	Pseudomonas putida	Pseudomonas putida GB-1	cntB
HMP	Proteobacteria	Gammaproteobacteria	Enterobacteriales	Enterobacteriaceae	Shigella	Shigella sonnei	Shigella sonnei Ss046	cntB
HMP	Proteobacteria	Gammaproteobacteria	Enterobacteriales	Enterobacteriaceae	Yersinia	Yersinia pestis	Yersinia pestis biovar Microtus str. 91001	cntB
HMP	Firmicutes	Clostridia	Clostridiales	Clostridiaceae	Clostridium	Clostridium tetani	Clostridium tetani E88	cutC
HMP	Firmicutes	Clostridia	Clostridiales	Lachnospiraceae	Lachnoclostridium	Lachnoclostridium phytofermentans	Lachnoclostridium phytofermentans ISDg	cutC
HMP	Firmicutes	Clostridia	Clostridiales	Clostridiaceae	Alkaliphilus	Alkaliphilus oremlandii	Alkaliphilus oremlandii OhILAs	cutC
HMP	Firmicutes	Clostridia	Clostridiales	Clostridiaceae	Alkaliphilus	Alkaliphilus metalliredigens	Alkaliphilus metalliredigens QYMF	cutC
HMP	Proteobacteria	Deltaproteobacteria	Desulfuromonadales	Desulfuromonadaceae	Pelobacter	Pelobacter carbinolicus	Pelobacter carbinolicus DSM 2380	cutC
HMP	Firmicutes	Clostridia	Clostridiales	Peptococcaceae	Desulfitobacterium	Desulfitobacterium hafniense	Desulfitobacterium hafniense Y51	cutC
HMP	Proteobacteria	Deltaproteobacteria	Desulfovibrionales	Desulfovibrionaceae	Desulfovibrio	Desulfovibrio alaskensis	Desulfovibrio alaskensis G20	cutC
HMP	Proteobacteria	Deltaproteobacteria	Desulfovibrionales	Desulfovibrionaceae	Desulfovibrio	Desulfovibrio alaskensis	Desulfovibrio alaskensis G20	cutC
HMP	Proteobacteria	Deltaproteobacteria	Desulfobacterales	Desulfobulbaceae	Desulfotalea	Desulfotalea psychrophila	Desulfotalea psychrophila LSv54	cutC
HMP	Firmicutes	Clostridia	Clostridiales	Clostridiaceae	Clostridium	Clostridium botulinum	Clostridium botulinum A2 str. Kyoto	cutC
HMP	Firmicutes	Clostridia	Clostridiales	Eubacteriales	unclassified Eubacteriales	Clostridiales bacterium 1_7_47FAA	Clostridiales bacterium 1_7_47FAA	cutC
HMP	Firmicutes	Clostridia	Clostridiales	Clostridiaceae	Clostridium	Clostridium botulinum	Clostridium botulinum NCTC 2916	cutC
HMP	Firmicutes	Clostridia	Clostridiales	Clostridiaceae	Clostridium	Clostridium botulinum	Clostridium botulinum Bf	cutC
HMP	Firmicutes	Clostridia	Clostridiales	Clostridiaceae	Clostridium	Clostridium sporogenes	Clostridium sporogenes ATCC 15579	cutC
HMP	Firmicutes	Clostridia	Clostridiales	Clostridiaceae	Clostridium	Clostridium botulinum	Clostridium botulinum Ba4 str. 657	cutC
HMP	Proteobacteria	Gammaproteobacteria	Enterobacteriales	Enterobacteriaceae	Proteus	Proteus penneri	Proteus penneri ATCC 35198	cutC
HMP	Proteobacteria	Gammaproteobacteria	Enterobacteriales	Enterobacteriaceae	Enterobacter	Enterobacter cancerogenus	Enterobacter cancerogenus ATCC 35316	cutC

HMP	*Firmicutes*	*Tissierellia*	*Tissierellales*	*Peptoniphilaceae*	*Anaerococcus*	*Anaerococcus hydrogenalis*	*Anaerococcus hydrogenalis DSM 7454*	*cutC*
HMP	*Proteobacteria*	*Gammaproteobacteria*	*Enterobacteriales*	*Enterobacteriaceae*	*Providencia*	*Providencia rustigianii*	*Providencia rustigianii DSM 4541*	*cutC*
HMP	*Proteobacteria*	*Gammaproteobacteria*	*Enterobacteriales*	*Enterobacteriaceae*	*Providencia*	*Providencia alcalifaciens*	*Providencia alcalifaciens DSM 30120*	*cutC*
HMP	*Proteobacteria*	*Gammaproteobacteria*	*Enterobacteriales*	*Enterobacteriaceae*	*Pectobacterium*	*Pectobacterium carotovorum*	*Pectobacterium carotovorum subsp. carotovorum PC1*	*cutC*
HMP	*Firmicutes*	*Clostridia*	*Clostridiales*	*Clostridiaceae*	*Clostridium*	*Clostridium botulinum*	*Clostridium botulinum E3 str. Alaska E43*	*cutC*
HMP	*Proteobacteria*	*Deltaproteobacteria*	*Desulfovibrionales*	*Desulfovibrionaceae*	*Desulfovibrio*	*Desulfovibrio salexigens*	*Desulfovibrio salexigens DSM 2638*	*cutC*
HMP	*Firmicutes*	*Clostridia*	*Clostridiales*	*Lachnospiraceae*	*Lachnoclostridium*	*[Clostridium] asparagiforme*	*[Clostridium asparagiforme] DSM 15981*	*cutC*
HMP	*Proteobacteria*	*Deltaproteobacteria*	*Desulfovibrionales*	*Desulfovibrionaceae*	*Desulfovibrio*	*Desulfovibrio salexigens*	*Desulfovibrio salexigens DSM 2638*	*cutC*
HMP	*Firmicutes*	*Tissierellia*	*Tissierellales*	*Peptoniphilaceae*	*Anaerococcus*	*Anaerococcus tetradius*	*Anaerococcus tetradius ATCC 35098*	*cutC*
HMP	*Firmicutes*	*Clostridia*	*Clostridiales*	*Clostridiaceae*	*Hungatella*	*Hungatella hathewayi*	*Hungatella hathewayi DSM 13479*	*cutC*
HMP	*Firmicutes*	*Clostridia*	*Clostridiales*	*Clostridiaceae*	*Clostridium*	*Clostridium botulinum*	*Clostridium botulinum B str. Eklund 17B (NRP)*	*cutC*
HMP	*Firmicutes*	*Clostridia*	*Clostridiales*	*Clostridiaceae*	*Clostridium*	*Clostridium botulinum*	*Clostridium botulinum E1 str. 'BoNT E Beluga'*	*cutC*
HMP	*Firmicutes*	*Clostridia*	*Clostridiales*	*Clostridiaceae*	*Clostridium*	*Clostridium botulinum*	*Clostridium botulinum D str. 1873*	*cutC*
HMP	*Firmicutes*	*Clostridia*	*Clostridiales*	*Clostridiaceae*	*Clostridium*	*Clostridium carboxidivorans*	*Clostridium carboxidivorans P7*	*cutC*
HMP	*Firmicutes*	*Tissierellia*	*Tissierellales*	*Peptoniphilaceae*	*Anaerococcus*	*Anaerococcus vaginalis*	*Anaerococcus vaginalis ATCC 51170*	*cutC*
HMP	*Proteobacteria*	*Gammaproteobacteria*	*Enterobacteriales*	*Enterobacteriaceae*	*Klebsiella*	*Klebsiella pneumoniae*	*Klebsiella pneumoniae subsp. rhinoscleromatis ATCC 13884*	*cutC*
HMP	*Firmicutes*	*Clostridia*	*Clostridiales*	*Clostridiaceae*	*Clostridium*	*Clostridium botulinum*	*Clostridium botulinum B1 str. Okra*	*cutC*
HMP	*Proteobacteria*	*Deltaproteobacteria*	*Desulfovibrionales*	*Desulfovibrionaceae*	*Desulfovibrio*	*Desulfovibrio desulfuricans*	*Desulfovibrio desulfuricans subsp. desulfuricans str. ATCC 27774*	*cutC*
HMP	*Proteobacteria*	*Gammaproteobacteria*	*Enterobacteriales*	*Enterobacteriaceae*	*Providencia*	*Providencia rettgeri*	*Providencia rettgeri DSM 1131*	*cutC*
HMP	*Firmicutes*	*Clostridia*	*Clostridiales*	*Clostridiaceae*	*Clostridium*	*Clostridium carboxidivorans*	*Clostridium carboxidivorans P7*	*cutC*
HMP	*Proteobacteria*	*Gammaproteobacteria*	*Enterobacteriales*	*Enterobacteriaceae*	*Escherichia*	*Escherichia fergusonii*	*Escherichia fergusonii ATCC 35469*	*cutC*
HMP	*Proteobacteria*	*Gammaproteobacteria*	*Enterobacteriales*	*Enterobacteriaceae*	*Escherichia*	*Escherichia coli*	*Escherichia coli MS 69-1*	*cutC*
HMP	*Proteobacteria*	*Gammaproteobacteria*	*Enterobacteriales*	*Enterobacteriaceae*	*Escherichia*	*Escherichia coli*	*Escherichia coli MS 200-1*	*cutC*
HMP	*Firmicutes*	*Clostridia*	*Clostridiales*	*Lachnospiraceae*	*Lachnoclostridium*	*[Clostridium] saccharolyticum*	*[Clostridium] saccharolyticum WM1*	*cutC*
HMP	*Actinobacteria*	*Coriobacteriia*	*Coriobacteriales*	*Atopobiaceae*	*Olsenella*	*Olsenella uli*	*Olsenella uli DSM 7084*	*cutC*
HMP	*Firmicutes*	*Clostridia*	*Clostridiales*	*Clostridiaceae*	*Clostridium*	*Clostridium ljungdahlii*	*Clostridium ljungdahlii DSM 13528*	*cutC*
HMP	*Firmicutes*	*Clostridia*	*Clostridiales*	*Clostridiaceae*	*Clostridium*	*Clostridium botulinum*	*Clostridium botulinum F str. Langeland*	*cutC*
HMP	*Proteobacteria*	*Gammaproteobacteria*	*Enterobacteriales*	*Enterobacteriaceae*	*Klebsiella*	*Klebsiella variicola*	*Klebsiella variicola At-22*	*cutC*
HMP	*Proteobacteria*	*Gammaproteobacteria*	*Enterobacteriales*	*Enterobacteriaceae*	*Pectobacterium*	*Pectobacterium wasabiae*	*Pectobacterium wasabiae WPP163*	*cutC*
HMP	*Firmicutes*	*Clostridia*	*Clostridiales*	*Peptococcaceae*	*Desulfotomaculum*	*Desulfotomaculum reducens*	*Desulfotomaculum reducens MI-1*	*cutC*
HMP	*Proteobacteria*	*Gammaproteobacteria*	*Enterobacteriales*	*Enterobacteriaceae*	*Proteus*	*Proteus mirabilis*	*Proteus mirabilis ATCC 29906*	*cutC*
HMP	*Proteobacteria*	*Gammaproteobacteria*	*Aeromonadales*	*Aeromonadaceae*	*Aeromonas*	*Aeromonas hydrophila*	*Aeromonas hydrophila subsp. hydrophila ATCC 7966*	*cutC*
HMP	*Proteobacteria*	*Gammaproteobacteria*	*Enterobacteriales*	*Enterobacteriaceae*	*Pectobacterium*	*Pectobacterium atrosepticum*	*Pectobacterium atrosepticum SCRI1043*	*cutC*
HMP	*Firmicutes*	*Clostridia*	*Clostridiales*	*Clostridiaceae*	*Clostridium*	*Clostridium tetani*	*Clostridium tetani E88*	*cutD*
HMP	*Firmicutes*	*Clostridia*	*Clostridiales*	*Lachnospiraceae*	*Lachnoclostridium*	*Lachnoclostridium phytofermentans*	*Lachnoclostridium phytofermentans ISDg*	*cutD*
HMP	*Firmicutes*	*Clostridia*	*Clostridiales*	*Clostridiaceae*	*Alkaliphilus*	*Alkaliphilus oremlandii*	*Alkaliphilus oremlandii OhILAs*	*cutD*
HMP	*Firmicutes*	*Clostridia*	*Clostridiales*	*Clostridiaceae*	*Alkaliphilus*	*Alkaliphilus metalliredigens*	*Alkaliphilus metalliredigens QYMF*	*cutD*
HMP	*Proteobacteria*	*Deltaproteobacteria*	*Desulfuromonadales*	*Desulfuromonadaceae*	*Pelobacter*	*Pelobacter carbinolicus*	*Pelobacter carbinolicus DSM 2380*	*cutD*
HMP	*Firmicutes*	*Clostridia*	*Clostridiales*	*Peptococcaceae*	*Desulfitobacterium*	*Desulfitobacterium hafniense*	*Desulfitobacterium hafniense Y51*	*cutD*
HMP	*Proteobacteria*	*Deltaproteobacteria*	*Desulfovibrionales*	*Desulfovibrionaceae*	*Desulfovibrio*	*Desulfovibrio alaskensis*	*Desulfovibrio alaskensis G20*	*cutD*
HMP	*Proteobacteria*	*Deltaproteobacteria*	*Desulfobacterales*	*Desulfobulbaceae*	*Desulfotalea*	*Desulfotalea psychrophila*	*Desulfotalea psychrophila LSv54*	*cutD*
HMP	*Firmicutes*	*Clostridia*	*Clostridiales*	*Clostridiaceae*	*Clostridium*	*Clostridium botulinum*	*Clostridium botulinum A2 str. Kyoto*	*cutD*
HMP	*Firmicutes*	*Clostridia*	*Clostridiales*	*Eubacteriales*	*unclassified Eubacteriales*	*Clostridiales bacterium 1_7_47FAA*	*Clostridiales bacterium 1_7_47FAA*	*cutD*
HMP	*Firmicutes*	*Clostridia*	*Clostridiales*	*Clostridiaceae*	*Clostridium*	*Clostridium botulinum*	*Clostridium botulinum NCTC 2916*	*cutD*
HMP	*Firmicutes*	*Clostridia*	*Clostridiales*	*Clostridiaceae*	*Clostridium*	*Clostridium botulinum*	*Clostridium botulinum Bf*	*cutD*
HMP	*Firmicutes*	*Clostridia*	*Clostridiales*	*Clostridiaceae*	*Clostridium*	*Clostridium sporogenes*	*Clostridium sporogenes ATCC 15579*	*cutD*
HMP	*Firmicutes*	*Clostridia*	*Clostridiales*	*Clostridiaceae*	*Clostridium*	*Clostridium botulinum*	*Clostridium botulinum Ba4 str. 657*	*cutD*
HMP	*Proteobacteria*	*Gammaproteobacteria*	*Enterobacteriales*	*Enterobacteriaceae*	*Proteus*	*Proteus penneri*	*Proteus penneri ATCC 35198*	*cutD*
HMP	*Firmicutes*	*Tissierellia*	*Tissierellales*	*Peptoniphilaceae*	*Anaerococcus*	*Anaerococcus hydrogenalis*	*Anaerococcus hydrogenalis DSM 7454*	*cutD*
HMP	*Proteobacteria*	*Gammaproteobacteria*	*Enterobacteriales*	*Enterobacteriaceae*	*Providencia*	*Providencia rustigianii*	*Providencia rustigianii DSM 4541*	*cutD*
HMP	*Proteobacteria*	*Gammaproteobacteria*	*Enterobacteriales*	*Enterobacteriaceae*	*Providencia*	*Providencia alcalifaciens*	*Providencia alcalifaciens DSM 30120*	*cutD*
HMP	*Proteobacteria*	*Gammaproteobacteria*	*Enterobacteriales*	*Enterobacteriaceae*	*Pectobacterium*	*Pectobacterium carotovorum*	*Pectobacterium carotovorum subsp. carotovorum PC1*	*cutD*
HMP	*Firmicutes*	*Clostridia*	*Clostridiales*	*Clostridiaceae*	*Clostridium*	*Clostridium botulinum*	*Clostridium botulinum E3 str. Alaska E43*	*cutD*
HMP	*Proteobacteria*	*Deltaproteobacteria*	*Desulfovibrionales*	*Desulfovibrionaceae*	*Desulfovibrio*	*Desulfovibrio salexigens*	*Desulfovibrio salexigens DSM 2638*	*cutD*
HMP	*Firmicutes*	*Clostridia*	*Clostridiales*	*Lachnospiraceae*	*Lachnoclostridium*	*[Clostridium] asparagiforme*	*[Clostridium asparagiforme] DSM 15981*	*cutD*
HMP	*Firmicutes*	*Tissierellia*	*Tissierellales*	*Peptoniphilaceae*	*Anaerococcus*	*Anaerococcus tetradius*	*Anaerococcus tetradius ATCC 35098*	*cutD*
HMP	*Firmicutes*	*Clostridia*	*Clostridiales*	*Clostridiaceae*	*Hungatella*	*Hungatella hathewayi*	*Hungatella hathewayi DSM 13479*	*cutD*
HMP	*Firmicutes*	*Clostridia*	*Clostridiales*	*Clostridiaceae*	*Clostridium*	*Clostridium botulinum*	*Clostridium botulinum B str. Eklund 17B (NRP)*	*cutD*
HMP	*Firmicutes*	*Clostridia*	*Clostridiales*	*Clostridiaceae*	*Clostridium*	*Clostridium botulinum*	*Clostridium botulinum E1 str. 'BoNT E Beluga'*	*cutD*
HMP	*Firmicutes*	*Clostridia*	*Clostridiales*	*Clostridiaceae*	*Clostridium*	*Clostridium botulinum*	*Clostridium botulinum D str. 1873*	*cutD*
HMP	*Firmicutes*	*Clostridia*	*Clostridiales*	*Clostridiaceae*	*Clostridium*	*Clostridium carboxidivorans*	*Clostridium carboxidivorans P7*	*cutD*
HMP	*Firmicutes*	*Tissierellia*	*Tissierellales*	*Peptoniphilaceae*	*Anaerococcus*	*Anaerococcus vaginalis*	*Anaerococcus vaginalis ATCC 51170*	*cutD*
HMP	*Proteobacteria*	*Gammaproteobacteria*	*Enterobacteriales*	*Enterobacteriaceae*	*Klebsiella*	*Klebsiella pneumoniae*	*Klebsiella pneumoniae subsp. rhinoscleromatis ATCC 13884*	*cutD*
HMP	*Firmicutes*	*Clostridia*	*Clostridiales*	*Clostridiaceae*	*Clostridium*	*Clostridium botulinum*	*Clostridium botulinum B1 str. Okra*	*cutD*
HMP	*Proteobacteria*	*Deltaproteobacteria*	*Desulfovibrionales*	*Desulfovibrionaceae*	*Desulfovibrio*	*Desulfovibrio desulfuricans*	*Desulfovibrio desulfuricans subsp. desulfuricans str. ATCC 27774*	*cutD*
HMP	*Proteobacteria*	*Gammaproteobacteria*	*Enterobacteriales*	*Enterobacteriaceae*	*Providencia*	*Providencia rettgeri*	*Providencia rettgeri DSM 1131*	*cutD*
HMP	*Firmicutes*	*Clostridia*	*Clostridiales*	*Clostridiaceae*	*Clostridium*	*Clostridium carboxidivorans*	*Clostridium carboxidivorans P7*	*cutD*
HMP	*Proteobacteria*	*Gammaproteobacteria*	*Enterobacteriales*	*Enterobacteriaceae*	*Escherichia*	*Escherichia fergusonii*	*Escherichia fergusonii ATCC 35469*	*cutD*
HMP	*Proteobacteria*	*Gammaproteobacteria*	*Enterobacteriales*	*Enterobacteriaceae*	*Escherichia*	*Escherichia coli*	*Escherichia coli MS 69-1*	*cutD*
HMP	*Proteobacteria*	*Gammaproteobacteria*	*Enterobacteriales*	*Enterobacteriaceae*	*Escherichia*	*Escherichia coli*	*Escherichia coli MS 200-1*	*cutD*
HMP	*Firmicutes*	*Clostridia*	*Clostridiales*	*Lachnospiraceae*	*Lachnoclostridium*	*[Clostridium] saccharolyticum*	*[Clostridium] saccharolyticum WM1*	*cutD*
HMP	*Actinobacteria*	*Coriobacteriia*	*Coriobacteriales*	*Atopobiaceae*	*Olsenella*	*Olsenella uli*	*Olsenella uli DSM 7084*	*cutD*
HMP	*Firmicutes*	*Clostridia*	*Clostridiales*	*Clostridiaceae*	*Clostridium*	*Clostridium ljungdahlii*	*Clostridium ljungdahlii DSM 13528*	*cutD*
HMP	*Firmicutes*	*Clostridia*	*Clostridiales*	*Clostridiaceae*	*Clostridium*	*Clostridium botulinum*	*Clostridium botulinum F str. Langeland*	*cutD*
HMP	*Proteobacteria*	*Gammaproteobacteria*	*Enterobacteriales*	*Enterobacteriaceae*	*Klebsiella*	*Klebsiella variicola*	*Klebsiella variicola At-22*	*cutD*
HMP	*Proteobacteria*	*Gammaproteobacteria*	*Enterobacteriales*	*Enterobacteriaceae*	*Pectobacterium*	*Pectobacterium wasabiae*	*Pectobacterium wasabiae WPP163*	*cutD*
HMP	*Firmicutes*	*Clostridia*	*Clostridiales*	*Peptococcaceae*	*Desulfotomaculum*	*Desulfotomaculum reducens*	*Desulfotomaculum reducens MI-1*	*cutD*
HMP	*Proteobacteria*	*Gammaproteobacteria*	*Enterobacteriales*	*Enterobacteriaceae*	*Proteus*	*Proteus mirabilis*	*Proteus mirabilis ATCC 29906*	*cutD*
HMP	*Proteobacteria*	*Gammaproteobacteria*	*Aeromonadales*	*Aeromonadaceae*	*Aeromonas*	*Aeromonas hydrophila*	*Aeromonas hydrophila subsp. hydrophila ATCC 7966*	*cutD*
HMP	*Proteobacteria*	*Gammaproteobacteria*	*Enterobacteriales*	*Enterobacteriaceae*	*Pectobacterium*	*Pectobacterium atrosepticum*	*Pectobacterium atrosepticum SCRI1043*	*cutD*
HMP	*Proteobacteria*	*Gammaproteobacteria*	*Enterobacteriales*	*Enterobacteriaceae*	*Shigella*	*Shigella flexneri*	*Shigella flexneri 2a str. 301*	*yeaW*
HMP	*Proteobacteria*	*Gammaproteobacteria*	*Pseudomonadales*	*Moraxellaceae*	*Acinetobacter*	*Acinetobacter baumannii*	*Acinetobacter baumannii AB307-0294*	*yeaW*
HMP	*Proteobacteria*	*Gammaproteobacteria*	*Pseudomonadales*	*Moraxellaceae*	*Acinetobacter*	*Acinetobacter baumannii*	*Acinetobacter baumannii AB0057*	*yeaW*
HMP	*Proteobacteria*	*Gammaproteobacteria*	*Pseudomonadales*	*Moraxellaceae*	*Acinetobacter*	*Acinetobacter baumannii*	*Acinetobacter baumannii AYE*	*yeaW*
HMP	*Proteobacteria*	*Betaproteobacteria*	*Burkholderiales*	*Burkholderiaceae*	*Cupriavidus*	*Cupriavidus necator*	*Ralstonia eutropha H16*	*yeaW*
HMP	*Proteobacteria*	*Gammaproteobacteria*	*Pseudomonadales*	*Pseudomonadaceae*	*Pseudomonas*	*Pseudomonas entomophila*	*Pseudomonas entomophila L48*	*yeaW*
HMP	*Proteobacteria*	*Betaproteobacteria*	*Burkholderiales*	*Burkholderiaceae*	*Cupriavidus*	*Cupriavidus pinatubonensis*	*Ralstonia eutropha JMP134*	*yeaW*
HMP	*Proteobacteria*	*Gammaproteobacteria*	*Enterobacteriales*	*Enterobacteriaceae*	*Citrobacter*	*Citrobacter sp. 30_2*	*Citrobacter sp. 30_2*	*yeaW*
HMP	*Proteobacteria*	*Gammaproteobacteria*	*Pseudomonadales*	*Moraxellaceae*	*Acinetobacter*	*Acinetobacter baumannii*	*Acinetobacter baumannii ATCC 19606 = CIP 70.34 = JCM 6841*	*yeaW*
HMP	*Proteobacteria*	*Gammaproteobacteria*	*Pseudomonadales*	*Moraxellaceae*	*Acinetobacter*	*Acinetobacter nosocomialis*	*Acinetobacter sp. RUH2624*	*yeaW*
HMP	*Proteobacteria*	*Gammaproteobacteria*	*Enterobacteriales*	*Enterobacteriaceae*	*Yersinia*	*Yersinia pestis*	*Yersinia pestis biovar Orientalis str. IP275*	*yeaW*
HMP	*Proteobacteria*	*Gammaproteobacteria*	*Enterobacteriales*	*Enterobacteriaceae*	*Yersinia*	*Yersinia pestis*	*Yersinia pestis biovar Antiqua str. E1979001*	*yeaW*
HMP	*Proteobacteria*	*Gammaproteobacteria*	*Enterobacteriales*	*Enterobacteriaceae*	*Escherichia*	*Escherichia coli*	*Escherichia coli SE11*	*yeaW*
HMP	*Proteobacteria*	*Gammaproteobacteria*	*Enterobacteriales*	*Enterobacteriaceae*	*Providencia*	*Providencia stuartii*	*Providencia stuartii ATCC 25827*	*yeaW*
HMP	*Proteobacteria*	*Gammaproteobacteria*	*Enterobacteriales*	*Enterobacteriaceae*	*Escherichia*	*Escherichia coli*	*Escherichia coli O157:H7 str. EC4024*	*yeaW*
HMP	*Proteobacteria*	*Gammaproteobacteria*	*Enterobacteriales*	*Enterobacteriaceae*	*Escherichia*	*Escherichia albertii*	*Escherichia albertii TW07627*	*yeaW*
HMP	*Proteobacteria*	*Gammaproteobacteria*	*Enterobacteriales*	*Enterobacteriaceae*	*Escherichia*	*Escherichia coli*	*Escherichia coli D9*	*yeaW*
HMP	*Proteobacteria*	*Gammaproteobacteria*	*Enterobacteriales*	*Enterobacteriaceae*	*Escherichia*	*Escherichia sp. 4_1_40B*	*Escherichia sp. 4_1_40B*	*yeaW*
HMP	*Proteobacteria*	*Gammaproteobacteria*	*Enterobacteriales*	*Enterobacteriaceae*	*Yersinia*	*Yersinia pestis*	*Yersinia pestis Pestoides A*	*yeaW*
HMP	*Proteobacteria*	*Gammaproteobacteria*	*Enterobacteriales*	*Enterobacteriaceae*	*Klebsiella*	*Klebsiella pneumoniae*	*Klebsiella pneumoniae subsp. rhinoscleromatis ATCC 13884*	*yeaW*
HMP	*Proteobacteria*	*Betaproteobacteria*	*Burkholderiales*	*Alcaligenaceae*	*Achromobacter*	*Achromobacter piechaudii*	*Achromobacter piechaudii ATCC 43553*	*yeaW*
HMP	*Proteobacteria*	*Gammaproteobacteria*	*Enterobacteriales*	*Enterobacteriaceae*	*Yersinia*	*Yersinia pestis*	*Yersinia pestis biovar Mediaevalis str. K1973002*	*yeaW*
HMP	*Proteobacteria*	*Gammaproteobacteria*	*Enterobacteriales*	*Enterobacteriaceae*	*Escherichia*	*Escherichia coli*	*Escherichia coli OP50*	*yeaW*
HMP	*Proteobacteria*	*Gammaproteobacteria*	*Enterobacteriales*	*Enterobacteriaceae*	*Yersinia*	*Yersinia pestis*	*Yersinia pestis KIM D27*	*yeaW*
HMP	*Proteobacteria*	*Gammaproteobacteria*	*Pseudomonadales*	*Moraxellaceae*	*Acinetobacter*	*Acinetobacter baumannii*	*Acinetobacter baumannii AB056*	*yeaW*
HMP	*Proteobacteria*	*Gammaproteobacteria*	*Pseudomonadales*	*Moraxellaceae*	*Acinetobacter*	*Acinetobacter baumannii*	*Acinetobacter baumannii AB058*	*yeaW*
HMP	*Proteobacteria*	*Gammaproteobacteria*	*Pseudomonadales*	*Moraxellaceae*	*Acinetobacter*	*Acinetobacter baumannii*	*Acinetobacter baumannii AB059*	*yeaW*
HMP	*Proteobacteria*	*Gammaproteobacteria*	*Enterobacteriales*	*Enterobacteriaceae*	*Escherichia*	*Escherichia coli*	*Escherichia coli MS 124-1*	*yeaW*
HMP	*Proteobacteria*	*Gammaproteobacteria*	*Enterobacteriales*	*Enterobacteriaceae*	*Escherichia*	*Escherichia coli*	*Escherichia coli MS 119-7*	*yeaW*
HMP	*Proteobacteria*	*Gammaproteobacteria*	*Enterobacteriales*	*Enterobacteriaceae*	*Escherichia*	*Escherichia coli*	*Escherichia coli MS 107-1*	*yeaW*
HMP	*Proteobacteria*	*Gammaproteobacteria*	*Pseudomonadales*	*Moraxellaceae*	*Acinetobacter*	*Acinetobacter baumannii*	*Acinetobacter baumannii 6013150*	*yeaW*
HMP	*Proteobacteria*	*Gammaproteobacteria*	*Pseudomonadales*	*Moraxellaceae*	*Acinetobacter*	*Acinetobacter baumannii*	*Acinetobacter baumannii 6013113*	*yeaW*
HMP	*Proteobacteria*	*Gammaproteobacteria*	*Pseudomonadales*	*Moraxellaceae*	*Acinetobacter*	*Acinetobacter baumannii*	*Acinetobacter baumannii 6014059*	*yeaW*
HMP	*Proteobacteria*	*Gammaproteobacteria*	*Enterobacteriales*	*Enterobacteriaceae*	*Providencia*	*Providencia rettgeri*	*Providencia rettgeri DSM 1131*	*yeaW*
HMP	*Proteobacteria*	*Gammaproteobacteria*	*Enterobacteriales*	*Enterobacteriaceae*	*Citrobacter*	*Citrobacter youngae*	*Citrobacter youngae ATCC 29220*	*yeaW*
HMP	*Proteobacteria*	*Gammaproteobacteria*	*Enterobacteriales*	*Enterobacteriaceae*	*Klebsiella*	*Klebsiella sp. 1_1_55*	*Klebsiella sp. 1_1_55*	*yeaW*
HMP	*Proteobacteria*	*Gammaproteobacteria*	*Enterobacteriales*	*Enterobacteriaceae*	*Escherichia*	*Escherichia coli*	*Escherichia coli B088*	*yeaW*
HMP	*Proteobacteria*	*Gammaproteobacteria*	*Enterobacteriales*	*Enterobacteriaceae*	*Escherichia*	*Escherichia coli*	*Escherichia coli B185*	*yeaW*
HMP	*Proteobacteria*	*Gammaproteobacteria*	*Enterobacteriales*	*Enterobacteriaceae*	*Escherichia*	*Escherichia coli*	*Escherichia coli FVEC1412*	*yeaW*
HMP	*Proteobacteria*	*Gammaproteobacteria*	*Enterobacteriales*	*Enterobacteriaceae*	*Escherichia*	*Escherichia coli*	*Escherichia coli B354*	*yeaW*
HMP	*Proteobacteria*	*Gammaproteobacteria*	*Pseudomonadales*	*Moraxellaceae*	*Acinetobacter*	*Acinetobacter pittii*	*Acinetobacter sp. SH024*	*yeaW*
HMP	*Proteobacteria*	*Gammaproteobacteria*	*Enterobacteriales*	*Enterobacteriaceae*	*Escherichia*	*Escherichia coli*	*Escherichia coli MS 198-1*	*yeaW*
HMP	*Proteobacteria*	*Gammaproteobacteria*	*Enterobacteriales*	*Enterobacteriaceae*	*Escherichia*	*Escherichia coli*	*Escherichia coli MS 84-1*	*yeaW*
HMP	*Proteobacteria*	*Gammaproteobacteria*	*Enterobacteriales*	*Enterobacteriaceae*	*Escherichia*	*Escherichia coli*	*Escherichia coli MS 115-1*	*yeaW*
HMP	*Proteobacteria*	*Gammaproteobacteria*	*Enterobacteriales*	*Enterobacteriaceae*	*Escherichia*	*Escherichia coli*	*Escherichia coli MS 182-1*	*yeaW*
HMP	*Proteobacteria*	*Gammaproteobacteria*	*Enterobacteriales*	*Enterobacteriaceae*	*Escherichia*	*Escherichia coli*	*Escherichia coli MS 146-1*	*yeaW*
HMP	*Proteobacteria*	*Gammaproteobacteria*	*Enterobacteriales*	*Enterobacteriaceae*	*Escherichia*	*Escherichia coli*	*Escherichia coli MS 69-1*	*yeaW*
HMP	*Proteobacteria*	*Gammaproteobacteria*	*Enterobacteriales*	*Enterobacteriaceae*	*Escherichia*	*Escherichia coli*	*Escherichia coli MS 187-1*	*yeaW*
HMP	*Proteobacteria*	*Gammaproteobacteria*	*Enterobacteriales*	*Enterobacteriaceae*	*Escherichia*	*Escherichia coli*	*Escherichia coli MS 21-1*	*yeaW*
HMP	*Proteobacteria*	*Gammaproteobacteria*	*Enterobacteriales*	*Enterobacteriaceae*	*Escherichia*	*Escherichia coli*	*Escherichia coli MS 78-1*	*yeaW*
HMP	*Proteobacteria*	*Gammaproteobacteria*	*Enterobacteriales*	*Enterobacteriaceae*	*Escherichia*	*Escherichia coli*	*Escherichia coli MS 116-1*	*yeaW*
HMP	*Proteobacteria*	*Gammaproteobacteria*	*Enterobacteriales*	*Enterobacteriaceae*	*Escherichia*	*Escherichia coli*	*Escherichia coli MS 175-1*	*yeaW*
HMP	*Proteobacteria*	*Gammaproteobacteria*	*Enterobacteriales*	*Enterobacteriaceae*	*Escherichia*	*Escherichia coli*	*Escherichia coli MS 196-1*	*yeaW*
HMP	*Proteobacteria*	*Gammaproteobacteria*	*Enterobacteriales*	*Enterobacteriaceae*	*Escherichia*	*Escherichia coli*	*Escherichia coli FVEC1302*	*yeaW*
HMP	*Proteobacteria*	*Gammaproteobacteria*	*Enterobacteriales*	*Enterobacteriaceae*	*Escherichia*	*Escherichia coli*	*Escherichia coli str. K-12 substr. MG1655*	*yeaW*
HMP	*Proteobacteria*	*Gammaproteobacteria*	*Enterobacteriales*	*Enterobacteriaceae*	*Xenorhabdus*	*Xenorhabdus nematophila*	*Xenorhabdus nematophila ATCC 19061*	*yeaW*
HMP	*Proteobacteria*	*Gammaproteobacteria*	*Enterobacteriales*	*Enterobacteriaceae*	*Yersinia*	*Yersinia pestis*	*Yersinia pestis Z176003*	*yeaW*
HMP	*Proteobacteria*	*Betaproteobacteria*	*Burkholderiales*	*Burkholderiaceae*	*Burkholderia*	*Burkholderia multivorans*	*Burkholderia multivorans ATCC 17616*	*yeaW*
HMP	*Proteobacteria*	*Gammaproteobacteria*	*Pseudomonadales*	*Moraxellaceae*	*Acinetobacter*	*Acinetobacter oleivorans*	*Acinetobacter oleivorans DR1*	*yeaW*
HMP	*Proteobacteria*	*Gammaproteobacteria*	*Enterobacteriales*	*Enterobacteriaceae*	*Escherichia*	*Escherichia coli*	*Escherichia coli O55:H7 str. CB9615*	*yeaW*
HMP	*Proteobacteria*	*Gammaproteobacteria*	*Enterobacteriales*	*Enterobacteriaceae*	*Klebsiella*	*Klebsiella variicola*	*Klebsiella variicola At-22*	*yeaW*
HMP	*Proteobacteria*	*Gammaproteobacteria*	*Enterobacteriales*	*Enterobacteriaceae*	*Escherichia*	*Escherichia coli*	*Escherichia coli O26:H11 str. 11368*	*yeaW*
HMP	*Proteobacteria*	*Gammaproteobacteria*	*Enterobacteriales*	*Enterobacteriaceae*	*Shigella*	*Shigella flexneri*	*Shigella flexneri 2002017*	*yeaW*
HMP	*Proteobacteria*	*Gammaproteobacteria*	*Enterobacteriales*	*Enterobacteriaceae*	*Escherichia*	*Escherichia coli*	*Escherichia coli 042*	*yeaW*
HMP	*Proteobacteria*	*Gammaproteobacteria*	*Pseudomonadales*	*Moraxellaceae*	*Acinetobacter*	*Acinetobacter baumannii*	*Acinetobacter baumannii ATCC 17978*	*yeaW*
HMP	*Proteobacteria*	*Gammaproteobacteria*	*Enterobacteriales*	*Enterobacteriaceae*	*Shigella*	*Shigella boydii*	*Shigella boydii CDC 3083-94*	*yeaW*
HMP	*Proteobacteria*	*Gammaproteobacteria*	*Enterobacteriales*	*Enterobacteriaceae*	*Yersinia*	*Yersinia pseudotuberculosis*	*Yersinia pseudotuberculosis IP 32953*	*yeaW*
HMP	*Proteobacteria*	*Gammaproteobacteria*	*Pseudomonadales*	*Pseudomonadaceae*	*Pseudomonas*	*Pseudomonas putida*	*Pseudomonas putida GB-1*	*yeaW*
HMP	*Proteobacteria*	*Gammaproteobacteria*	*Enterobacteriales*	*Enterobacteriaceae*	*Shigella*	*Shigella sonnei*	*Shigella sonnei Ss046*	*yeaW*
HMP	*Proteobacteria*	*Gammaproteobacteria*	*Enterobacteriales*	*Enterobacteriaceae*	*Yersinia*	*Yersinia pestis*	*Yersinia pestis biovar Microtus str. 91001*	*yeaW*
HMP	*Proteobacteria*	*Gammaproteobacteria*	*Enterobacteriales*	*Enterobacteriaceae*	*Shigella*	*Shigella flexneri*	*Shigella flexneri 2a str. 301*	*yeaX*
HMP	*Proteobacteria*	*Gammaproteobacteria*	*Pseudomonadales*	*Moraxellaceae*	*Acinetobacter*	*Acinetobacter baumannii*	*Acinetobacter baumannii AB307-0294*	*yeaX*
HMP	*Proteobacteria*	*Gammaproteobacteria*	*Pseudomonadales*	*Moraxellaceae*	*Acinetobacter*	*Acinetobacter baumannii*	*Acinetobacter baumannii AB0057*	*yeaX*
HMP	*Proteobacteria*	*Gammaproteobacteria*	*Pseudomonadales*	*Moraxellaceae*	*Acinetobacter*	*Acinetobacter baumannii*	*Acinetobacter baumannii AYE*	*yeaX*
HMP	*Proteobacteria*	*Betaproteobacteria*	*Burkholderiales*	*Burkholderiaceae*	*Cupriavidus*	*Cupriavidus necator*	*Ralstonia eutropha H16*	*yeaX*
HMP	*Proteobacteria*	*Gammaproteobacteria*	*Pseudomonadales*	*Pseudomonadaceae*	*Pseudomonas*	*Pseudomonas entomophila*	*Pseudomonas entomophila L48*	*yeaX*
HMP	*Proteobacteria*	*Betaproteobacteria*	*Burkholderiales*	*Burkholderiaceae*	*Cupriavidus*	*Cupriavidus pinatubonensis*	*Ralstonia eutropha JMP134*	*yeaX*
HMP	*Proteobacteria*	*Gammaproteobacteria*	*Thiotrichales*	*Piscirickettsiaceae*	*Methylophaga*	*Methylophaga thiooxydans*	*Methylophaga thiooxydans DMS010*	*yeaX*
HMP	*Proteobacteria*	*Gammaproteobacteria*	*Enterobacteriales*	*Enterobacteriaceae*	*Citrobacter*	*Citrobacter sp. 30_2*	*Citrobacter sp. 30_2*	*yeaX*
HMP	*Proteobacteria*	*Gammaproteobacteria*	*Pseudomonadales*	*Moraxellaceae*	*Acinetobacter*	*Acinetobacter baumannii*	*Acinetobacter baumannii ATCC 19606 = CIP 70.34 = JCM 6841*	*yeaX*
HMP	*Proteobacteria*	*Gammaproteobacteria*	*Pseudomonadales*	*Moraxellaceae*	*Acinetobacter*	*Acinetobacter nosocomialis*	*Acinetobacter sp. RUH2624*	*yeaX*
HMP	*Proteobacteria*	*Gammaproteobacteria*	*Enterobacteriales*	*Enterobacteriaceae*	*Yersinia*	*Yersinia pestis*	*Yersinia pestis biovar Orientalis str. IP275*	*yeaX*
HMP	*Proteobacteria*	*Gammaproteobacteria*	*Enterobacteriales*	*Enterobacteriaceae*	*Yersinia*	*Yersinia pestis*	*Yersinia pestis biovar Antiqua str. E1979001*	*yeaX*
HMP	*Proteobacteria*	*Gammaproteobacteria*	*Enterobacteriales*	*Enterobacteriaceae*	*Escherichia*	*Escherichia coli*	*Escherichia coli SE11*	*yeaX*
HMP	*Proteobacteria*	*Gammaproteobacteria*	*Enterobacteriales*	*Enterobacteriaceae*	*Providencia*	*Providencia stuartii*	*Providencia stuartii ATCC 25827*	*yeaX*

HMP	Proteobacteria	Gammaproteobacteria	Enterobacteriales	Enterobacteriaceae	Escherichia	Escherichia coli	Escherichia coli O157:H7 str. EC4024	yeaX
HMP	Proteobacteria	Gammaproteobacteria	Enterobacteriales	Enterobacteriaceae	Escherichia	Escherichia albertii	Escherichia albertii TW07627	yeaX
HMP	Proteobacteria	Gammaproteobacteria	Enterobacteriales	Enterobacteriaceae	Escherichia	Escherichia coli	Escherichia coli D9	yeaX
HMP	Proteobacteria	Gammaproteobacteria	Enterobacteriales	Enterobacteriaceae	Escherichia	Escherichia sp. 4_1_40B	Escherichia sp. 4_1_40B	yeaX
HMP	Proteobacteria	Gammaproteobacteria	Enterobacteriales	Enterobacteriaceae	Yersinia	Yersinia pestis	Yersinia pestis Pestoides A	yeaX
HMP	Proteobacteria	Gammaproteobacteria	Enterobacteriales	Enterobacteriaceae	Klebsiella	Klebsiella pneumoniae	Klebsiella pneumoniae subsp. rhinoscleromatis ATCC 13884	yeaX
HMP	Proteobacteria	Betaproteobacteria	Burkholderiales	Alcaligenaceae	Achromobacter	Achromobacter piechaudii	Achromobacter piechaudii ATCC 43553	yeaX
HMP	Proteobacteria	Gammaproteobacteria	Enterobacteriales	Enterobacteriaceae	Yersinia	Yersinia pestis	Yersinia pestis biovar Mediaevalis str. K1973002	yeaX
HMP	Proteobacteria	Gammaproteobacteria	Enterobacteriales	Enterobacteriaceae	Escherichia	Escherichia coli	Escherichia coli OP50	yeaX
HMP	Proteobacteria	Gammaproteobacteria	Enterobacteriales	Enterobacteriaceae	Yersinia	Yersinia pestis	Yersinia pestis KIM D27	yeaX
HMP	Proteobacteria	Gammaproteobacteria	Pseudomonadales	Moraxellaceae	Acinetobacter	Acinetobacter baumannii	Acinetobacter baumannii AB056	yeaX
HMP	Proteobacteria	Gammaproteobacteria	Pseudomonadales	Moraxellaceae	Acinetobacter	Acinetobacter baumannii	Acinetobacter baumannii AB058	yeaX
HMP	Proteobacteria	Gammaproteobacteria	Pseudomonadales	Moraxellaceae	Acinetobacter	Acinetobacter baumannii	Acinetobacter baumannii AB059	yeaX
HMP	Proteobacteria	Gammaproteobacteria	Enterobacteriales	Enterobacteriaceae	Escherichia	Escherichia coli	Escherichia coli MS 124-1	yeaX
HMP	Proteobacteria	Gammaproteobacteria	Enterobacteriales	Enterobacteriaceae	Escherichia	Escherichia coli	Escherichia coli MS 119-7	yeaX
HMP	Proteobacteria	Gammaproteobacteria	Enterobacteriales	Enterobacteriaceae	Escherichia	Escherichia coli	Escherichia coli MS 107-1	yeaX
HMP	Proteobacteria	Gammaproteobacteria	Pseudomonadales	Moraxellaceae	Acinetobacter	Acinetobacter baumannii	Acinetobacter baumannii 6013150	yeaX
HMP	Proteobacteria	Gammaproteobacteria	Pseudomonadales	Moraxellaceae	Acinetobacter	Acinetobacter baumannii	Acinetobacter baumannii 6013113	yeaX
HMP	Proteobacteria	Gammaproteobacteria	Pseudomonadales	Moraxellaceae	Acinetobacter	Acinetobacter baumannii	Acinetobacter baumannii 6014059	yeaX
HMP	Proteobacteria	Gammaproteobacteria	Enterobacteriales	Enterobacteriaceae	Providencia	Providencia rettgeri	Providencia rettgeri DSM 1131	yeaX
HMP	Proteobacteria	Gammaproteobacteria	Enterobacteriales	Enterobacteriaceae	Citrobacter	Citrobacter youngae	Citrobacter youngae ATCC 29220	yeaX
HMP	Proteobacteria	Gammaproteobacteria	Enterobacteriales	Enterobacteriaceae	Klebsiella	Klebsiella sp. 1_1_55	Klebsiella sp. 1_1_55	yeaX
HMP	Proteobacteria	Gammaproteobacteria	Enterobacteriales	Enterobacteriaceae	Escherichia	Escherichia coli	Escherichia coli B088	yeaX
HMP	Proteobacteria	Gammaproteobacteria	Enterobacteriales	Enterobacteriaceae	Escherichia	Escherichia coli	Escherichia coli B185	yeaX
HMP	Proteobacteria	Gammaproteobacteria	Enterobacteriales	Enterobacteriaceae	Escherichia	Escherichia coli	Escherichia coli FVEC1412	yeaX
HMP	Proteobacteria	Gammaproteobacteria	Enterobacteriales	Enterobacteriaceae	Escherichia	Escherichia coli	Escherichia coli B354	yeaX
HMP	Proteobacteria	Gammaproteobacteria	Pseudomonadales	Moraxellaceae	Acinetobacter	Acinetobacter pittii	Acinetobacter sp. SH024	yeaX
HMP	Proteobacteria	Gammaproteobacteria	Enterobacteriales	Enterobacteriaceae	Escherichia	Escherichia coli	Escherichia coli MS 198-1	yeaX
HMP	Proteobacteria	Gammaproteobacteria	Enterobacteriales	Enterobacteriaceae	Escherichia	Escherichia coli	Escherichia coli MS 84-1	yeaX
HMP	Proteobacteria	Gammaproteobacteria	Enterobacteriales	Enterobacteriaceae	Escherichia	Escherichia coli	Escherichia coli MS 115-1	yeaX
HMP	Proteobacteria	Gammaproteobacteria	Enterobacteriales	Enterobacteriaceae	Escherichia	Escherichia coli	Escherichia coli MS 182-1	yeaX
HMP	Proteobacteria	Gammaproteobacteria	Enterobacteriales	Enterobacteriaceae	Escherichia	Escherichia coli	Escherichia coli MS 146-1	yeaX
HMP	Proteobacteria	Gammaproteobacteria	Enterobacteriales	Enterobacteriaceae	Escherichia	Escherichia coli	Escherichia coli MS 69-1	yeaX
HMP	Proteobacteria	Gammaproteobacteria	Enterobacteriales	Enterobacteriaceae	Escherichia	Escherichia coli	Escherichia coli MS 187-1	yeaX
HMP	Proteobacteria	Gammaproteobacteria	Enterobacteriales	Enterobacteriaceae	Escherichia	Escherichia coli	Escherichia coli MS 21-1	yeaX
HMP	Proteobacteria	Gammaproteobacteria	Enterobacteriales	Enterobacteriaceae	Escherichia	Escherichia coli	Escherichia coli MS 78-1	yeaX
HMP	Proteobacteria	Gammaproteobacteria	Enterobacteriales	Enterobacteriaceae	Escherichia	Escherichia coli	Escherichia coli MS 116-1	yeaX
HMP	Proteobacteria	Gammaproteobacteria	Enterobacteriales	Enterobacteriaceae	Escherichia	Escherichia coli	Escherichia coli MS 175-1	yeaX
HMP	Proteobacteria	Gammaproteobacteria	Enterobacteriales	Enterobacteriaceae	Escherichia	Escherichia coli	Escherichia coli MS 196-1	yeaX
HMP	Proteobacteria	Gammaproteobacteria	Enterobacteriales	Enterobacteriaceae	Escherichia	Escherichia coli	Escherichia coli FVEC1302	yeaX
HMP	Proteobacteria	Gammaproteobacteria	Enterobacteriales	Enterobacteriaceae	Escherichia	Escherichia coli	Escherichia coli str. K-12 substr. MG1655	yeaX
HMP	Proteobacteria	Gammaproteobacteria	Enterobacteriales	Enterobacteriaceae	Xenorhabdus	Xenorhabdus nematophila	Xenorhabdus nematophila ATCC 19061	yeaX
HMP	Proteobacteria	Gammaproteobacteria	Enterobacteriales	Enterobacteriaceae	Yersinia	Yersinia pestis	Yersinia pestis Z176003	yeaX
HMP	Proteobacteria	Betaproteobacteria	Burkholderiales	Burkholderiaceae	Burkholderia	Burkholderia multivorans	Burkholderia multivorans ATCC 17616	yeaX
HMP	Proteobacteria	Betaproteobacteria	Methylophilales	Methylophilaceae	Methylotenera	Methylotenera versatilis	Methylotenera versatilis 301	yeaX
HMP	Proteobacteria	Gammaproteobacteria	Pseudomonadales	Moraxellaceae	Acinetobacter	Acinetobacter oleivorans	Acinetobacter oleivorans DR1	yeaX
HMP	Proteobacteria	Gammaproteobacteria	Enterobacteriales	Enterobacteriaceae	Escherichia	Escherichia coli	Escherichia coli O55:H7 str. CB9615	yeaX
HMP	Proteobacteria	Gammaproteobacteria	Pseudomonadales	Moraxellaceae	Acinetobacter	Acinetobacter baumannii	Acinetobacter baumannii SDF	yeaX
HMP	Proteobacteria	Gammaproteobacteria	Enterobacteriales	Enterobacteriaceae	Klebsiella	Klebsiella variicola	Klebsiella variicola At-22	yeaX
HMP	Proteobacteria	Gammaproteobacteria	Enterobacteriales	Enterobacteriaceae	Escherichia	Escherichia coli	Escherichia coli O26:H11 str. 11368	yeaX
HMP	Proteobacteria	Gammaproteobacteria	Enterobacteriales	Enterobacteriaceae	Shigella	Shigella flexneri	Shigella flexneri 2002017	yeaX
HMP	Proteobacteria	Gammaproteobacteria	Enterobacteriales	Enterobacteriaceae	Escherichia	Escherichia coli	Escherichia coli 042	yeaX
HMP	Proteobacteria	Gammaproteobacteria	Pseudomonadales	Moraxellaceae	Acinetobacter	Acinetobacter baumannii	Acinetobacter baumannii ATCC 17978	yeaX
HMP	Proteobacteria	Gammaproteobacteria	Enterobacteriales	Enterobacteriaceae	Shigella	Shigella boydii	Shigella boydii CDC 3083-94	yeaX
HMP	Proteobacteria	Gammaproteobacteria	Enterobacteriales	Enterobacteriaceae	Yersinia	Yersinia pseudotuberculosis	Yersinia pseudotuberculosis IP 32953	yeaX
HMP	Proteobacteria	Gammaproteobacteria	Pseudomonadales	Pseudomonadaceae	Pseudomonas	Pseudomonas putida	Pseudomonas putida GB-1	yeaX
HMP	Proteobacteria	Gammaproteobacteria	Enterobacteriales	Enterobacteriaceae	Shigella	Shigella sonnei	Shigella sonnei Ss046	yeaX
HMP	Proteobacteria	Gammaproteobacteria	Enterobacteriales	Enterobacteriaceae	Yersinia	Yersinia pestis	Yersinia pestis biovar Microtus str. 91001	yeaX
HMP	Firmicutes	Clostridia	Clostridiales	Clostridiaceae	Clostridium	Clostridium tetani	Clostridium tetani E88	cutD
HMP	Proteobacteria	Gammaproteobacteria	Alteromonadales	Shewanellaceae	Shewanella	Shewanella oneidensis	Shewanella oneidensis MR-1	cutD
HMP	Firmicutes	Clostridia	Halanaerobiales	Halanaerobiaceae	Halothermothrix	Halothermothrix orenii	Halothermothrix orenii H 168	cutD
HMP	Actinobacteria	Coriobacteriia	Eggerthellales	Eggerthellaceae	Eggerthella	Eggerthella lenta	Eggerthella lenta DSM 2243	cutD
HMP	Firmicutes	Clostridia	Clostridiales	Peptococcaceae	Candidatus Desulforudis	Candidatus Desulforudis audaxviator	Candidatus Desulforudis audaxviator MP104C	cutD
HMP	Proteobacteria	Deltaproteobacteria	Desulfuromonadales	Geobacteraceae	Geobacter	Geobacter uraniireducens	Geobacter uraniireducens Rf4	cutD
HMP	Proteobacteria	Deltaproteobacteria	Desulfuromonadales	Desulfuromonadaceae	Pelobacter	Pelobacter propionicus	Pelobacter propionicus DSM 2379	cutD
HMP	Dictyoglomi	Dictyoglomia	Dictyoglomales	Dictyoglomaceae	Dictyoglomus	Dictyoglomus thermophilum	Dictyoglomus thermophilum H-6-12	cutD
HMP	Proteobacteria	Deltaproteobacteria	Syntrophobacterales	Syntrophobacteraceae	Syntrophobacter	Syntrophobacter fumaroxidans	Syntrophobacter fumaroxidans MPOB	cutD
HMP	Proteobacteria	Deltaproteobacteria	Desulfuromonadales	Geobacteraceae	Geobacter	Geobacter metallireducens	Geobacter metallireducens GS-15	cutD
HMP	Proteobacteria	Deltaproteobacteria	Desulfuromonadales	Desulfuromonadaceae	Pelobacter	Pelobacter carbinolicus	Pelobacter carbinolicus DSM 2380	cutD
HMP	Proteobacteria	Betaproteobacteria	Hydrogenophilales	Hydrogenophilaceae	Thiobacillus	Thiobacillus denitrificans	Thiobacillus denitrificans ATCC 25259	cutD
HMP	Proteobacteria	Deltaproteobacteria	Desulfobacterales	Desulfobacteraceae	Desulfobacterium	Desulfobacterium autotrophicum	Desulfobacterium autotrophicum HRM2	cutD
HMP	Proteobacteria	Deltaproteobacteria	Desulfobacterales	Desulfobacteraceae	Desulfobacterium	Desulfobacterium autotrophicum	Desulfobacterium autotrophicum HRM2	cutD
HMP	Thermotogae	Thermotogae	Thermotogales	Thermotogaceae	Thermotoga	Thermotoga sp. RQ2	Thermotoga sp. RQ2	cutD
HMP	Firmicutes	Clostridia	Clostridiales	Peptococcaceae	Desulfitobacterium	Desulfitobacterium hafniense	Desulfitobacterium hafniense Y51	cutD
HMP	Proteobacteria	Betaproteobacteria	Rhodocyclales	Rhodocyclaceae	Dechloromonas	Dechloromonas aromatica	Dechloromonas aromatica RCB	cutD
HMP	Bacteroidetes	Bacteroidia	Bacteroidales	Bacteroidaceae	Bacteroides	Bacteroides thetaiotaomicron	Bacteroides thetaiotaomicron VPI-5482	cutD
HMP	Firmicutes	Clostridia	Clostridiales	Clostridiaceae	Clostridium	Clostridium botulinum	Clostridium botulinum A2 str. Kyoto	cutD
HMP	Thermotogae	Thermotogae	Petrotogales	NA	Marinitoga	Marinitoga piezophila	Marinitoga piezophila KA3	cutD
HMP	Bacteroidetes	Bacteroidia	Bacteroidales	Bacteroidaceae	Bacteroides	Bacteroides sp. 1_1_6	Bacteroides sp. 1_1_6	cutD
HMP	Proteobacteria	Deltaproteobacteria	Desulfuromonadales	Geobacteraceae	Geobacter	Geobacter bemidjiensis	Geobacter bemidjiensis Bem	cutD
HMP	Firmicutes	Negativicutes	Selenomonadales	Sporomusaceae	Thermosinus	Thermosinus carboxydivorans	Thermosinus carboxydivorans Nor1	cutD
HMP	Bacteroidetes	Bacteroidia	Bacteroidales	Bacteroidaceae	Bacteroides	Bacteroides ovatus	Bacteroides ovatus ATCC 8483	cutD
HMP	Firmicutes	Clostridia	Clostridiales	NA	Pseudoflavonifractor	Pseudoflavonifractor capillosus	Pseudoflavonifractor capillosus ATCC 29799	cutD
HMP	Bacteroidetes	Bacteroidia	Bacteroidales	Bacteroidaceae	Bacteroides	Bacteroides stercoris	Bacteroides stercoris ATCC 43183	cutD
HMP	Proteobacteria	Betaproteobacteria	Methylophilales	Methylophilaceae	Methylovorus	Methylovorus glucosotrophus	Methylovorus glucosetrophus SIP3-4	cutD
HMP	Proteobacteria	Gammaproteobacteria	Alteromonadales	Shewanellaceae	Shewanella	Shewanella benthica	Shewanella benthica KT99	cutD
HMP	Firmicutes	Clostridia	Clostridiales	Clostridiaceae	Clostridium	Clostridium sporogenes	Clostridium sporogenes ATCC 15579	cutD
HMP	Proteobacteria	Alphaproteobacteria	Rhizobiales	Rhizobiaceae	Rhizobium	Rhizobium etli	Rhizobium etli Kim 5	cutD
HMP	Proteobacteria	Alphaproteobacteria	Rhizobiales	Rhizobiaceae	Rhizobium	Rhizobium etli	Rhizobium etli CIAT 894	cutD
HMP	Firmicutes	Clostridia	Clostridiales	Peptostreptococcaceae	Peptoclostridium	[Clostridium] hiranonis	[Clostridium] hiranonis DSM 13275	cutD
HMP	Proteobacteria	Deltaproteobacteria	Desulfuromonadales	Geobacteraceae	Geobacter	Geobacter sp. M21	Geobacter sp. M21	cutD
HMP	Proteobacteria	Gammaproteobacteria	Enterobacteriales	Enterobacteriaceae	Dickeya	Dickeya chrysanthemi	Dickeya chrysanthemi Ech1591	cutD
HMP	Bacteroidetes	Bacteroidia	Bacteroidales	Bacteroidaceae	Bacteroides	Bacteroides coprophilus	Bacteroides coprophilus DSM 18228 = JCM 13818	cutD
HMP	Bacteroidetes	Bacteroidia	Bacteroidales	Bacteroidaceae	Bacteroides	Bacteroides sp. D2	Bacteroides sp. D2	cutD
HMP	Proteobacteria	Deltaproteobacteria	Desulfovibrionales	Desulfovibrionaceae	Desulfovibrio	Desulfovibrio magneticus	Desulfovibrio magneticus RS-1	cutD
HMP	Firmicutes	Clostridia	Clostridiales	Clostridiaceae	Clostridium	Clostridium carboxidivorans	Clostridium carboxidivorans P7	cutD
HMP	Firmicutes	Clostridia	Clostridiales	Clostridiaceae	Clostridium	Clostridium carboxidivorans	Clostridium carboxidivorans P7	cutD
HMP	Firmicutes	Clostridia	Clostridiales	Ruminococcaceae	Ruminiclostridium	Ruminiclostridium thermocellum	Ruminiclostridium thermocellum DSM 2360	cutD
HMP	Firmicutes	Clostridia	Clostridiales	Ruminococcaceae	Ruminiclostridium	[Clostridium] papyrosolvens	[Clostridium] papyrosolvens DSM 2782	cutD
HMP	Firmicutes	Clostridia	Clostridiales	Ruminococcaceae	Ruminiclostridium	Ruminiclostridium thermocellum	Ruminiclostridium thermocellum JW20	cutD
HMP	Proteobacteria	Gammaproteobacteria	Vibrionales	Vibrionaceae	Vibrio	Vibrio alginolyticus	Vibrio alginolyticus 40B	cutD
HMP	Proteobacteria	Deltaproteobacteria	Desulfovibrionales	Desulfovibrionaceae	Desulfovibrio	Desulfovibrio aespoeensis	Desulfovibrio aespoeensis Aspo-2	cutD
HMP	Proteobacteria	Deltaproteobacteria	Desulfovibrionales	Desulfovibrionaceae	Desulfovibrio	Desulfovibrio sp. FW1012B	Desulfovibrio sp. FW1012B	cutD
HMP	Bacteroidetes	Bacteroidia	Bacteroidales	Bacteroidaceae	Bacteroides	Bacteroides ovatus	Bacteroides ovatus SD CMC 3f	cutD
HMP	Proteobacteria	Deltaproteobacteria	Desulfuromonadales	Geobacteraceae	Geobacter	Geobacter daltonii	Geobacter daltonii FRC-32	cutD
HMP	Proteobacteria	Deltaproteobacteria	Desulfobacterales	Desulfobacteraceae	Desulfatibacillum	Desulfatibacillum alkenivorans	Desulfatibacillum alkenivorans AK-01	cutD
HMP	Firmicutes	Clostridia	Clostridiales	Clostridiaceae	Clostridium	Clostridium carboxidivorans	Clostridium carboxidivorans P7	cutD
HMP	Firmicutes	Clostridia	Clostridiales	Clostridiaceae	Clostridium	Clostridium carboxidivorans	Clostridium carboxidivorans P7	cutD
HMP	Bacteroidetes	Bacteroidia	Bacteroidales	Bacteroidaceae	Bacteroides	Bacteroides sp. 1_1_14	Bacteroides sp. 1_1_14	cutD
HMP	Bacteroidetes	Bacteroidia	Bacteroidales	Bacteroidaceae	Bacteroides	Bacteroides sp. 3_1_23	Bacteroides sp. 3_1_23	cutD
HMP	Proteobacteria	Deltaproteobacteria	Syntrophobacterales	Syntrophaceae	Syntrophus	Syntrophus aciditrophicus	Syntrophus aciditrophicus SB	cutD
HMP	Thermotogae	Thermotogae	Thermotogales	Thermotogaceae	Thermotoga	Thermotoga maritima	Thermotoga maritima MSB8	cutD
HMP	Proteobacteria	Deltaproteobacteria	Desulfuromonadales	Geobacteraceae	Geobacter	Geobacter sulfurreducens	Geobacter sulfurreducens PCA	cutD
HMP	Proteobacteria	Deltaproteobacteria	Desulfuromonadales	Geobacteraceae	Geobacter	Geobacter sulfurreducens	Geobacter sulfurreducens PCA	cutD
HMP	Spirochaetes	Spirochaetia	Brachyspirales	Brachyspiraceae	Brachyspira	Brachyspira pilosicoli	Brachyspira pilosicoli 95/1000	cutD
HMP	Firmicutes	Clostridia	Clostridiales	Clostridiaceae	Clostridium	Clostridium ljungdahlii	Clostridium ljungdahlii DSM 13528	cutD
HMP	Proteobacteria	Epsilonproteobacteria	Campylobacterales	Campylobacteraceae	Arcobacter	Arcobacter nitrofigilis	Arcobacter nitrofigilis DSM 7299	cutD
HMP	Firmicutes	Clostridia	Clostridiales	Peptococcaceae	Thermincola	Thermincola potens	Thermincola potens JR	cutD
HMP	Bacteroidetes	Bacteroidia	Bacteroidales	Prevotellaceae	Prevotella	Prevotella ruminicola	Prevotella ruminicola 23	cutD
HMP	Thermotogae	Thermotogae	Thermotogales	Thermotogaceae	Thermotoga	Thermotoga naphthophila	Thermotoga naphthophila RKU-10	cutD
HMP	Thermotogae	Thermotogae	Thermotogales	Thermotogaceae	Thermotoga	Thermotoga petrophila	Thermotoga petrophila RKU-1	cutD
HMP	Firmicutes	Clostridia	Clostridiales	Symbiobacteriaceae	Symbiobacterium	Symbiobacterium thermophilum	Symbiobacterium thermophilum IAM 14863	cutD
HMP	Proteobacteria	Deltaproteobacteria	Myxococcales	Polyangiaceae	Sorangium	Sorangium cellulosum	Sorangium cellulosum So ce56	cutD
UHGG	Actinobacteria	Coriobacteriia	Coriobacteriales	Atopobiaceae	Atopobium	Atopobium minutum	MGYG-HGUT-04259	cutD
UHGG	Actinobacteria	Coriobacteriia	Coriobacteriales	Coriobacteriaceae	Collinsella	Collinsella bouchesdurhonensis	MGYG-HGUT-01236	cutC
UHGG	Actinobacteria	Coriobacteriia	Coriobacteriales	Coriobacteriaceae	Collinsella	Collinsella tanakaei	MGYG-HGUT-01382	cutC
UHGG	Actinobacteria	Coriobacteriia	Coriobacteriales	Coriobacteriaceae	Collinsella	Collinsella bouchesdurhonensis	MGYG-HGUT-01236	cutD
UHGG	Actinobacteria	Coriobacteriia	Coriobacteriales	Coriobacteriaceae	Collinsella	Collinsella tanakaei	MGYG-HGUT-01382	cutD
UHGG	Desulfobacterota_	Desulfovibrionia	Desulfovibrionales	Desulfovibrionaceae	Desulfovibrio	Desulfovibrio desulfuricans	MGYG-HGUT-02158	cutC
UHGG	Desulfobacterota_	Desulfovibrionia	Desulfovibrionales	Desulfovibrionaceae	Desulfovibrio	Desulfovibrio desulfuricans	MGYG-HGUT-02620	cutC
UHGG	Desulfobacterota_	Desulfovibrionia	Desulfovibrionales	Desulfovibrionaceae	Desulfovibrio	Desulfovibrio desulfuricans	MGYG-HGUT-02158	cutD
UHGG	Desulfobacterota_	Desulfovibrionia	Desulfovibrionales	Desulfovibrionaceae	Desulfovibrio	Desulfovibrio desulfuricans	MGYG-HGUT-02620	cutD
UHGG	Desulfobacterota_	Desulfovibrionia	Desulfovibrionales	Desulfovibrionaceae	Desulfovibrio	Desulfovibrio desulfuricans	MGYG-HGUT-03933	cutD
UHGG	Firmicutes	Bacilli	Lactobacillales	Enterococcaceae	Enterococcus	Enterococcus asini	MGYG-HGUT-00163	cutC
UHGG	Firmicutes	Bacilli	Lactobacillales	Enterococcaceae	Enterococcus	Enterococcus dispar	MGYG-HGUT-01208	cutC
UHGG	Firmicutes	Bacilli	Lactobacillales	Enterococcaceae	Enterococcus	Enterococcus raffinosus	MGYG-HGUT-01696	cutC
UHGG	Firmicutes	Bacilli	Lactobacillales	Enterococcaceae	Enterococcus	Enterococcus avium	MGYG-HGUT-02270	cutC
UHGG	Firmicutes	Bacilli	Lactobacillales	Enterococcaceae	Enterococcus	Enterococcus caccae	MGYG-HGUT-02468	cutC
UHGG	Firmicutes	Bacilli	Lactobacillales	Enterococcaceae	Enterococcus	Enterococcus asini	MGYG-HGUT-00163	cutD
UHGG	Firmicutes	Bacilli	Lactobacillales	Enterococcaceae	Enterococcus	Enterococcus dispar	MGYG-HGUT-01208	cutD
UHGG	Firmicutes	Bacilli	Lactobacillales	Enterococcaceae	Enterococcus	Enterococcus raffinosus	MGYG-HGUT-01696	cutD
UHGG	Firmicutes	Bacilli	Lactobacillales	Enterococcaceae	Enterococcus	Enterococcus avium	MGYG-HGUT-02270	cutD
UHGG	Firmicutes	Bacilli	Lactobacillales	Enterococcaceae	Enterococcus	Enterococcus caccae	MGYG-HGUT-02468	cutD
UHGG	Firmicutes	Clostridia	Christensenellales	Christensenellaceae	Christensenella_A	Christensenella_A timonensis	MGYG-HGUT-01550	cutD
UHGG	Firmicutes	Clostridia	Christensenellales	Christensenellaceae	Christensenella_A	Christensenella_A timonensis	MGYG-HGUT-01550	cutD
UHGG	Firmicutes	Clostridia	Clostridiales	Clostridiaceae	Clostridium	Clostridium baratii	MGYG-HGUT-00064	cutC
UHGG	Firmicutes	Clostridia	Clostridiales	Clostridiaceae	Clostridium	Clostridium paraputrificum	MGYG-HGUT-00151	cutC
UHGG	Firmicutes	Clostridia	Clostridiales	Clostridiaceae	Clostridium	Clostridium botulinum	MGYG-HGUT-01309	cutC
UHGG	Firmicutes	Clostridia	Clostridiales	Clostridiaceae	Clostridium	Clostridium senegalense	MGYG-HGUT-01407	cutC
UHGG	Firmicutes	Clostridia	Clostridiales	Clostridiaceae	Clostridium	Clostridium ihumii	MGYG-HGUT-01699	cutC
UHGG	Firmicutes	Clostridia	Clostridiales	Clostridiaceae	Clostridium	Clostridium botulinum	MGYG-HGUT-02355	cutC
UHGG	Firmicutes	Clostridia	Clostridiales	Clostridiaceae	Clostridium	Clostridium septicum	MGYG-HGUT-02373	cutC
UHGG	Firmicutes	Clostridia	Clostridiales	Clostridiaceae	Clostridium	Clostridium tetani	MGYG-HGUT-02375	cutC
UHGG	Firmicutes	Clostridia	Clostridiales	Clostridiaceae	Clostridium	Clostridium tepidum	MGYG-HGUT-03116	cutC
UHGG	Firmicutes	Clostridia	Clostridiales	Clostridiaceae	Clostridium	Clostridium baratii	MGYG-HGUT-00064	cutD
UHGG	Firmicutes	Clostridia	Clostridiales	Clostridiaceae	Clostridium	Clostridium paraputrificum	MGYG-HGUT-00151	cutD
UHGG	Firmicutes	Clostridia	Clostridiales	Clostridiaceae	Clostridium	Clostridium botulinum	MGYG-HGUT-01309	cutD
UHGG	Firmicutes	Clostridia	Clostridiales	Clostridiaceae	Clostridium	Clostridium senegalense	MGYG-HGUT-01407	cutD
UHGG	Firmicutes	Clostridia	Clostridiales	Clostridiaceae	Clostridium	Clostridium ihumii	MGYG-HGUT-01699	cutD
UHGG	Firmicutes	Clostridia	Clostridiales	Clostridiaceae	Clostridium	Clostridium botulinum	MGYG-HGUT-02355	cutD
UHGG	Firmicutes	Clostridia	Clostridiales	Clostridiaceae	Clostridium	Clostridium septicum	MGYG-HGUT-02373	cutD
UHGG	Firmicutes	Clostridia	Clostridiales	Clostridiaceae	Clostridium	Clostridium tetani	MGYG-HGUT-02375	cutD
UHGG	Firmicutes	Clostridia	Clostridiales	Clostridiaceae	Clostridium	Clostridium tepidum	MGYG-HGUT-03116	cutD
UHGG	Firmicutes	Clostridia	Lachnospirales	Lachnospiraceae	Lachnoclostridium	Clostridium lavalense	MGYG-HGUT-00172	cutC
UHGG	Firmicutes	Clostridia	Lachnospirales	Lachnospiraceae	Clostridium	Clostridium citroniae	MGYG-HGUT-00198	cutC

UHGG	*Firmicutes*	*Clostridia*	*Lachnospirales*	*Lachnospiraceae*	*Clostridium*	*Clostridium clostridioforme*	*MGYG-HGUT-01564*	*cutC*
UHGG	*Firmicutes*	*Clostridia*	*Lachnospirales*	*Lachnospiraceae*	*Lachnoclostridium*	*Clostridium asparagiforme*	*MGYG-HGUT-02328*	*cutC*
UHGG	*Firmicutes*	*Clostridia*	*Lachnospirales*	*Lachnospiraceae*	*Lachnoclostridium*	*Clostridium lavalense*	*MGYG-HGUT-00172*	*cutD*
UHGG	*Firmicutes*	*Clostridia*	*Lachnospirales*	*Lachnospiraceae*	*Clostridium*	*Clostridium citroniae*	*MGYG-HGUT-00198*	*cutD*
UHGG	*Firmicutes*	*Clostridia*	*Lachnospirales*	*Lachnospiraceae*	*Clostridium*	*Clostridium clostridioforme*	*MGYG-HGUT-01564*	*cutD*
UHGG	*Firmicutes*	*Clostridia*	*Lachnospirales*	*Lachnospiraceae*	*Lachnoclostridium*	*Clostridium asparagiforme*	*MGYG-HGUT-02328*	*cutD*
UHGG	*Firmicutes*	*Clostridia*	*Lachnospirales*	*Lachnospiraceae*	*Hungatella*	*Hungatella effluvii*	*MGYG-HGUT-00032*	*cutC*
UHGG	*Firmicutes*	*Clostridia*	*Lachnospirales*	*Lachnospiraceae*	*Hungatella*	*Hungatella hathewayi*	*MGYG-HGUT-00150*	*cutC*
UHGG	*Firmicutes*	*Clostridia*	*Lachnospirales*	*Lachnospiraceae*	*Hungatella*	*Hungatella effluvii*	*MGYG-HGUT-00032*	*cutD*
UHGG	*Firmicutes*	*Clostridia*	*Lachnospirales*	*Lachnospiraceae*	*Hungatella*	*Hungatella hathewayi*	*MGYG-HGUT-00150*	*cutD*
UHGG	*Firmicutes*	*Clostridia*	*Oscillospirales*	*Acutalibacteraceae*	*Anaeromassilibacillus*	*Anaeromassilibacillus sp002159845*	*MGYG-HGUT-02135*	*cutD*
UHGG	*Firmicutes*	*Clostridia*	*Oscillospirales*	*Acutalibacteraceae*	*Anaeromassilibacillus*	*Anaeromassilibacillus sp001305115*	*MGYG-HGUT-03004*	*cutD*
UHGG	*Firmicutes*	*Clostridia*	*Oscillospirales*	*Ruminococcaceae*	*Fournierella*	*Fournierella massiliensis*	*MGYG-HGUT-01527*	*cutC*
UHGG	*Firmicutes*	*Clostridia*	*Oscillospirales*	*Ruminococcaceae*	*Fournierella*	*Fournierella massiliensis*	*MGYG-HGUT-01527*	*cutD*
UHGG	*Firmicutes*	*Clostridia*	*Peptostreptococcales*	*Peptostreptococcaceae*	*Romboutsia*	*Romboutsia dakarense*	*MGYG-HGUT-01460*	*cutC*
UHGG	*Firmicutes*	*Clostridia*	*Peptostreptococcales*	*Peptostreptococcaceae*	*Romboutsia*	*Romboutsia dakarense*	*MGYG-HGUT-01460*	*cutD*
UHGG	*Firmicutes*	*Clostridia*	*Tissierellales*	*Helcococcaceae*	*Anaerococcus*	*Anaerococcus hydrogenalis*	*MGYG-HGUT-01355*	*cutC*
UHGG	*Firmicutes*	*Clostridia*	*Tissierellales*	*Helcococcaceae*	*Anaerococcus*	*Anaerococcus vaginalis*	*MGYG-HGUT-02315*	*cutC*
UHGG	*Firmicutes*	*Clostridia*	*Tissierellales*	*Helcococcaceae*	*Anaerococcus*	*Anaerococcus obesiensis*	*MGYG-HGUT-02316*	*cutC*
UHGG	*Firmicutes*	*Clostridia*	*Tissierellales*	*Helcococcaceae*	*Anaerococcus*	*Anaerococcus hydrogenalis*	*MGYG-HGUT-01355*	*cutD*
UHGG	*Firmicutes*	*Clostridia*	*Tissierellales*	*Helcococcaceae*	*Anaerococcus*	*Anaerococcus vaginalis*	*MGYG-HGUT-02315*	*cutD*
UHGG	*Firmicutes*	*Clostridia*	*Tissierellales*	*Helcococcaceae*	*Anaerococcus*	*Anaerococcus obesiensis*	*MGYG-HGUT-02316*	*cutD*
UHGG	*Firmicutes*	*Clostridia*	*Tissierellales*	*Peptoniphilaceae*	*Peptoniphilus_E*	*Peptoniphilus_E obesi*	*MGYG-HGUT-01414*	*cutD*
UHGG	*Firmicutes*	*Clostridia*	*Tissierellales*	*Sporanaerobacteraceae*	*Anaerosalibacter_A*	*Anaerosalibacter_A massiliensis*	*MGYG-HGUT-01473*	*cutC*
UHGG	*Firmicutes*	*Clostridia*	*Tissierellales*	*Sporanaerobacteraceae*	*Anaerosalibacter_A*	*Anaerosalibacter_A massiliensis*	*MGYG-HGUT-01473*	*cutD*
UHGG	*Firmicutes*	*Desulfitobacteriia*	*Desulfitobacteriales*	*Desulfitobacteriaceae*	*Desulfitobacterium*	*Desulfitobacterium hafniense*	*MGYG-HGUT-01388*	*cutC*
UHGG	*Firmicutes*	*Desulfitobacteriia*	*Desulfitobacteriales*	*Desulfitobacteriaceae*	*Desulfitobacterium*	*Desulfitobacterium hafniense*	*MGYG-HGUT-01388*	*cutD*
UHGG	*Firmicutes*	*Peptococcia*	*Peptococcales*	*Peptococcaceae*	*Peptococcus*	*Peptococcus niger*	*MGYG-HGUT-03185*	*cutC*
UHGG	*Firmicutes*	*Peptococcia*	*Peptococcales*	*Peptococcaceae*	*Peptococcus*	*Peptococcus niger*	*MGYG-HGUT-03185*	*cutD*
UHGG	*Firmicutes*	*Negativicutes*	*Veillonellales*	*Megasphaeraceae*	*Anaeroglobus*	*Anaeroglobus massiliensis*	*MGYG-HGUT-01559*	*cutC*
UHGG	*Firmicutes*	*Negativicutes*	*Veillonellales*	*Megasphaeraceae*	*Anaeroglobus*	*Anaeroglobus massiliensis*	*MGYG-HGUT-01559*	*cutD*
UHGG	*Firmicutes*	*Negativicutes*	*Veillonellales*	*Megasphaeraceae*	*Caecibacter*	*Caecibacter massiliensis*	*MGYG-HGUT-01561*	*cutC*
UHGG	*Firmicutes*	*Negativicutes*	*Veillonellales*	*Megasphaeraceae*	*Caecibacter*	*Caecibacter massiliensis*	*MGYG-HGUT-01561*	*cutD*
UHGG	*Firmicutes*	*Negativicutes*	*Veillonellales*	*Megasphaeraceae*	*Megasphaera*	*Megasphaera elsdenii*	*MGYG-HGUT-02547*	*cutD*
UHGG	*Firmicutes*	*Bacilli_A*	*Paenibacillales*	*Paenibacillaceae*	*Paenibacillus_B*	*Paenibacillus_B thiaminolyticus*	*MGYG-HGUT-02490*	*cutC*
UHGG	*Firmicutes*	*Bacilli_A*	*Paenibacillales*	*Paenibacillaceae*	*Paenibacillus_B*	*Paenibacillus_B thiaminolyticus*	*MGYG-HGUT-02490*	*cutD*
UHGG	*Proteobacteria*	*Gammaproteobacteria*	*Burkholderiales*	*Neisseriaceae*	*Vitreoscilla*	*Vitreoscilla massiliensis*	*MGYG-HGUT-01520*	*cntA*
UHGG	*Proteobacteria*	*Gammaproteobacteria*	*Burkholderiales*	*Neisseriaceae*	*Vitreoscilla*	*Vitreoscilla massiliensis*	*MGYG-HGUT-01520*	*cntB*
UHGG	*Proteobacteria*	*Gammaproteobacteria*	*Burkholderiales*	*Neisseriaceae*	*Vitreoscilla*	*Vitreoscilla massiliensis*	*MGYG-HGUT-01520*	*yeaW*
UHGG	*Proteobacteria*	*Gammaproteobacteria*	*Burkholderiales*	*Neisseriaceae*	*Vitreoscilla*	*Vitreoscilla massiliensis*	*MGYG-HGUT-01520*	*yeaX*
UHGG	*Proteobacteria*	*Gammaproteobacteria*	*Enterobacterales*	*Enterobacteriaceae*	*Citrobacter*	*Citrobacter portucalensis*	*MGYG-HGUT-01317*	*cntA*
UHGG	*Proteobacteria*	*Gammaproteobacteria*	*Enterobacterales*	*Enterobacteriaceae*	*Citrobacter*	*Citrobacter portucalensis*	*MGYG-HGUT-01705*	*cntA*
UHGG	*Proteobacteria*	*Gammaproteobacteria*	*Enterobacterales*	*Enterobacteriaceae*	*Citrobacter*	*Citrobacter europaeus*	*MGYG-HGUT-02495*	*cntA*
UHGG	*Proteobacteria*	*Gammaproteobacteria*	*Enterobacterales*	*Enterobacteriaceae*	*Citrobacter*	*Citrobacter braakii*	*MGYG-HGUT-02816*	*cntA*
UHGG	*Proteobacteria*	*Gammaproteobacteria*	*Enterobacterales*	*Enterobacteriaceae*	*Citrobacter*	*Citrobacter portucalensis*	*MGYG-HGUT-01317*	*cntB*
UHGG	*Proteobacteria*	*Gammaproteobacteria*	*Enterobacterales*	*Enterobacteriaceae*	*Citrobacter*	*Citrobacter portucalensis*	*MGYG-HGUT-01705*	*cntB*
UHGG	*Proteobacteria*	*Gammaproteobacteria*	*Enterobacterales*	*Enterobacteriaceae*	*Citrobacter*	*Citrobacter europaeus*	*MGYG-HGUT-02495*	*cntB*
UHGG	*Proteobacteria*	*Gammaproteobacteria*	*Enterobacterales*	*Enterobacteriaceae*	*Citrobacter*	*Citrobacter braakii*	*MGYG-HGUT-02816*	*cntB*
UHGG	*Proteobacteria*	*Gammaproteobacteria*	*Enterobacterales*	*Enterobacteriaceae*	*Citrobacter*	*Citrobacter freundii*	*MGYG-HGUT-00108*	*cutC*
UHGG	*Proteobacteria*	*Gammaproteobacteria*	*Enterobacterales*	*Enterobacteriaceae*	*Citrobacter*	*Citrobacter pasteurii*	*MGYG-HGUT-00124*	*cutC*
UHGG	*Proteobacteria*	*Gammaproteobacteria*	*Enterobacterales*	*Enterobacteriaceae*	*Citrobacter*	*Citrobacter freundii*	*MGYG-HGUT-00108*	*cutD*
UHGG	*Proteobacteria*	*Gammaproteobacteria*	*Enterobacterales*	*Enterobacteriaceae*	*Citrobacter*	*Citrobacter pasteurii*	*MGYG-HGUT-00124*	*cutD*
UHGG	*Proteobacteria*	*Gammaproteobacteria*	*Enterobacterales*	*Enterobacteriaceae*	*Citrobacter*	*Citrobacter portucalensis*	*MGYG-HGUT-01317*	*yeaW*
UHGG	*Proteobacteria*	*Gammaproteobacteria*	*Enterobacterales*	*Enterobacteriaceae*	*Citrobacter*	*Citrobacter portucalensis*	*MGYG-HGUT-01705*	*yeaW*
UHGG	*Proteobacteria*	*Gammaproteobacteria*	*Enterobacterales*	*Enterobacteriaceae*	*Citrobacter*	*Citrobacter europaeus*	*MGYG-HGUT-02495*	*yeaW*
UHGG	*Proteobacteria*	*Gammaproteobacteria*	*Enterobacterales*	*Enterobacteriaceae*	*Citrobacter*	*Citrobacter braakii*	*MGYG-HGUT-02816*	*yeaW*
UHGG	*Proteobacteria*	*Gammaproteobacteria*	*Enterobacterales*	*Enterobacteriaceae*	*Citrobacter*	*Citrobacter portucalensis*	*MGYG-HGUT-01317*	*yeaX*
UHGG	*Proteobacteria*	*Gammaproteobacteria*	*Enterobacterales*	*Enterobacteriaceae*	*Citrobacter*	*Citrobacter portucalensis*	*MGYG-HGUT-01705*	*yeaX*
UHGG	*Proteobacteria*	*Gammaproteobacteria*	*Enterobacterales*	*Enterobacteriaceae*	*Citrobacter*	*Citrobacter europaeus*	*MGYG-HGUT-02495*	*yeaX*
UHGG	*Proteobacteria*	*Gammaproteobacteria*	*Enterobacterales*	*Enterobacteriaceae*	*Citrobacter*	*Citrobacter braakii*	*MGYG-HGUT-02816*	*yeaX*
UHGG	*Proteobacteria*	*Gammaproteobacteria*	*Enterobacterales*	*Enterobacteriaceae*	*Enterobacter*	*Enterobacter cancerogenus*	*MGYG-HGUT-01318*	*cutC*
UHGG	*Proteobacteria*	*Gammaproteobacteria*	*Enterobacterales*	*Enterobacteriaceae*	*Escherichia*	*Escherichia coli*	*MGYG-HGUT-02506*	*cntA*
UHGG	*Proteobacteria*	*Gammaproteobacteria*	*Enterobacterales*	*Enterobacteriaceae*	*Escherichia*	*Escherichia albertii*	*MGYG-HGUT-02507*	*cntA*
UHGG	*Proteobacteria*	*Gammaproteobacteria*	*Enterobacterales*	*Enterobacteriaceae*	*Escherichia*	*Escherichia coli*	*MGYG-HGUT-02506*	*cntB*
UHGG	*Proteobacteria*	*Gammaproteobacteria*	*Enterobacterales*	*Enterobacteriaceae*	*Escherichia*	*Escherichia albertii*	*MGYG-HGUT-02507*	*cntB*
UHGG	*Proteobacteria*	*Gammaproteobacteria*	*Enterobacterales*	*Enterobacteriaceae*	*Escherichia*	*Escherichia fergusonii*	*MGYG-HGUT-02515*	*cutC*
UHGG	*Proteobacteria*	*Gammaproteobacteria*	*Enterobacterales*	*Enterobacteriaceae*	*Escherichia*	*Escherichia fergusonii*	*MGYG-HGUT-02515*	*cutD*
UHGG	*Proteobacteria*	*Gammaproteobacteria*	*Enterobacterales*	*Enterobacteriaceae*	*Escherichia*	*Escherichia coli*	*MGYG-HGUT-02506*	*yeaW*
UHGG	*Proteobacteria*	*Gammaproteobacteria*	*Enterobacterales*	*Enterobacteriaceae*	*Escherichia*	*Escherichia albertii*	*MGYG-HGUT-02507*	*yeaW*
UHGG	*Proteobacteria*	*Gammaproteobacteria*	*Enterobacterales*	*Enterobacteriaceae*	*Escherichia*	*Escherichia coli*	*MGYG-HGUT-02506*	*yeaX*
UHGG	*Proteobacteria*	*Gammaproteobacteria*	*Enterobacterales*	*Enterobacteriaceae*	*Escherichia*	*Escherichia albertii*	*MGYG-HGUT-02507*	*yeaX*
UHGG	*Proteobacteria*	*Gammaproteobacteria*	*Enterobacterales*	*Enterobacteriaceae*	*Franconibacter*	*Franconibacter helveticus*	*MGYG-HGUT-03115*	*cutC*
UHGG	*Proteobacteria*	*Gammaproteobacteria*	*Enterobacterales*	*Enterobacteriaceae*	*Klebsiella*	*Klebsiella aerogenes*	*MGYG-HGUT-02497*	*cntA*
UHGG	*Proteobacteria*	*Gammaproteobacteria*	*Enterobacterales*	*Enterobacteriaceae*	*Klebsiella*	*Klebsiella variicola*	*MGYG-HGUT-02511*	*cntA*
UHGG	*Proteobacteria*	*Gammaproteobacteria*	*Enterobacterales*	*Enterobacteriaceae*	*Klebsiella*	*Klebsiella pneumoniae*	*MGYG-HGUT-02538*	*cntA*
UHGG	*Proteobacteria*	*Gammaproteobacteria*	*Enterobacterales*	*Enterobacteriaceae*	*Klebsiella*	*Klebsiella aerogenes*	*MGYG-HGUT-02497*	*cntB*
UHGG	*Proteobacteria*	*Gammaproteobacteria*	*Enterobacterales*	*Enterobacteriaceae*	*Klebsiella*	*Klebsiella variicola*	*MGYG-HGUT-02511*	*cntB*
UHGG	*Proteobacteria*	*Gammaproteobacteria*	*Enterobacterales*	*Enterobacteriaceae*	*Klebsiella*	*Klebsiella pneumoniae*	*MGYG-HGUT-02538*	*cntB*
UHGG	*Proteobacteria*	*Gammaproteobacteria*	*Enterobacterales*	*Enterobacteriaceae*	*Klebsiella*	*Klebsiella grimontii*	*MGYG-HGUT-00093*	*cutC*
UHGG	*Proteobacteria*	*Gammaproteobacteria*	*Enterobacterales*	*Enterobacteriaceae*	*Klebsiella*	*Klebsiella oxytoca*	*MGYG-HGUT-00107*	*cutC*
UHGG	*Proteobacteria*	*Gammaproteobacteria*	*Enterobacterales*	*Enterobacteriaceae*	*Klebsiella*	*Klebsiella michiganensis*	*MGYG-HGUT-02351*	*cutC*
UHGG	*Proteobacteria*	*Gammaproteobacteria*	*Enterobacterales*	*Enterobacteriaceae*	*Klebsiella*	*Klebsiella aerogenes*	*MGYG-HGUT-02497*	*cutC*
UHGG	*Proteobacteria*	*Gammaproteobacteria*	*Enterobacterales*	*Enterobacteriaceae*	*Klebsiella*	*Klebsiella quasipneumoniae*	*MGYG-HGUT-02510*	*cutC*
UHGG	*Proteobacteria*	*Gammaproteobacteria*	*Enterobacterales*	*Enterobacteriaceae*	*Klebsiella*	*Klebsiella variicola*	*MGYG-HGUT-02511*	*cutC*
UHGG	*Proteobacteria*	*Gammaproteobacteria*	*Enterobacterales*	*Enterobacteriaceae*	*Klebsiella*	*Klebsiella grimontii*	*MGYG-HGUT-00093*	*cutD*
UHGG	*Proteobacteria*	*Gammaproteobacteria*	*Enterobacterales*	*Enterobacteriaceae*	*Klebsiella*	*Klebsiella oxytoca*	*MGYG-HGUT-00107*	*cutD*
UHGG	*Proteobacteria*	*Gammaproteobacteria*	*Enterobacterales*	*Enterobacteriaceae*	*Klebsiella*	*Klebsiella michiganensis*	*MGYG-HGUT-02351*	*cutD*
UHGG	*Proteobacteria*	*Gammaproteobacteria*	*Enterobacterales*	*Enterobacteriaceae*	*Klebsiella*	*Klebsiella aerogenes*	*MGYG-HGUT-02497*	*cutD*
UHGG	*Proteobacteria*	*Gammaproteobacteria*	*Enterobacterales*	*Enterobacteriaceae*	*Klebsiella*	*Klebsiella quasipneumoniae*	*MGYG-HGUT-02510*	*cutD*
UHGG	*Proteobacteria*	*Gammaproteobacteria*	*Enterobacterales*	*Enterobacteriaceae*	*Klebsiella*	*Klebsiella variicola*	*MGYG-HGUT-02511*	*cutD*
UHGG	*Proteobacteria*	*Gammaproteobacteria*	*Enterobacterales*	*Enterobacteriaceae*	*Klebsiella*	*Klebsiella aerogenes*	*MGYG-HGUT-02497*	*yeaW*
UHGG	*Proteobacteria*	*Gammaproteobacteria*	*Enterobacterales*	*Enterobacteriaceae*	*Klebsiella*	*Klebsiella variicola*	*MGYG-HGUT-02511*	*yeaW*
UHGG	*Proteobacteria*	*Gammaproteobacteria*	*Enterobacterales*	*Enterobacteriaceae*	*Klebsiella*	*Klebsiella pneumoniae*	*MGYG-HGUT-02538*	*yeaW*
UHGG	*Proteobacteria*	*Gammaproteobacteria*	*Enterobacterales*	*Enterobacteriaceae*	*Klebsiella*	*Klebsiella aerogenes*	*MGYG-HGUT-02497*	*yeaX*
UHGG	*Proteobacteria*	*Gammaproteobacteria*	*Enterobacterales*	*Enterobacteriaceae*	*Klebsiella*	*Klebsiella variicola*	*MGYG-HGUT-02511*	*yeaX*
UHGG	*Proteobacteria*	*Gammaproteobacteria*	*Enterobacterales*	*Enterobacteriaceae*	*Klebsiella*	*Klebsiella pneumoniae*	*MGYG-HGUT-02538*	*yeaX*
UHGG	*Proteobacteria*	*Gammaproteobacteria*	*Enterobacterales*	*Enterobacteriaceae*	*Kluyvera*	*Kluyvera ascorbata*	*MGYG-HGUT-02491*	*cutC*
UHGG	*Proteobacteria*	*Gammaproteobacteria*	*Enterobacterales*	*Enterobacteriaceae*	*Kluyvera*	*Kluyvera ascorbata*	*MGYG-HGUT-03358*	*cutC*
UHGG	*Proteobacteria*	*Gammaproteobacteria*	*Enterobacterales*	*Enterobacteriaceae*	*Kluyvera*	*Kluyvera ascorbata*	*MGYG-HGUT-02491*	*cutD*
UHGG	*Proteobacteria*	*Gammaproteobacteria*	*Enterobacterales*	*Enterobacteriaceae*	*Kluyvera*	*Kluyvera ascorbata*	*MGYG-HGUT-03358*	*cutD*
UHGG	*Proteobacteria*	*Gammaproteobacteria*	*Enterobacterales*	*Enterobacteriaceae*	*Leclercia*	*Leclercia adecarboxylata*	*MGYG-HGUT-03187*	*cutC*
UHGG	*Proteobacteria*	*Gammaproteobacteria*	*Enterobacterales*	*Enterobacteriaceae*	*Leclercia*	*Leclercia adecarboxylata*	*MGYG-HGUT-03187*	*cutD*
UHGG	*Proteobacteria*	*Gammaproteobacteria*	*Enterobacterales*	*Enterobacteriaceae*	*Moellerella*	*Moellerella wisconsensis*	*MGYG-HGUT-02354*	*cutC*
UHGG	*Proteobacteria*	*Gammaproteobacteria*	*Enterobacterales*	*Enterobacteriaceae*	*Moellerella*	*Moellerella wisconsensis*	*MGYG-HGUT-02354*	*cutD*
UHGG	*Proteobacteria*	*Gammaproteobacteria*	*Enterobacterales*	*Enterobacteriaceae*	*Proteus*	*Proteus terrae*	*MGYG-HGUT-01624*	*cutC*
UHGG	*Proteobacteria*	*Gammaproteobacteria*	*Enterobacterales*	*Enterobacteriaceae*	*Proteus*	*Proteus penneri*	*MGYG-HGUT-02488*	*cutC*
UHGG	*Proteobacteria*	*Gammaproteobacteria*	*Enterobacterales*	*Enterobacteriaceae*	*Proteus*	*Proteus mirabilis*	*MGYG-HGUT-02514*	*cutC*
UHGG	*Proteobacteria*	*Gammaproteobacteria*	*Enterobacterales*	*Enterobacteriaceae*	*Proteus*	*Proteus vulgaris*	*MGYG-HGUT-02516*	*cutC*
UHGG	*Proteobacteria*	*Gammaproteobacteria*	*Enterobacterales*	*Enterobacteriaceae*	*Proteus*	*Proteus terrae*	*MGYG-HGUT-01624*	*cutD*
UHGG	*Proteobacteria*	*Gammaproteobacteria*	*Enterobacterales*	*Enterobacteriaceae*	*Proteus*	*Proteus penneri*	*MGYG-HGUT-02488*	*cutD*
UHGG	*Proteobacteria*	*Gammaproteobacteria*	*Enterobacterales*	*Enterobacteriaceae*	*Proteus*	*Proteus mirabilis*	*MGYG-HGUT-02514*	*cutD*
UHGG	*Proteobacteria*	*Gammaproteobacteria*	*Enterobacterales*	*Enterobacteriaceae*	*Proteus*	*Proteus vulgaris*	*MGYG-HGUT-02516*	*cutD*
UHGG	*Proteobacteria*	*Gammaproteobacteria*	*Enterobacterales*	*Enterobacteriaceae*	*Providencia*	*Providencia stuartii*	*MGYG-HGUT-01307*	*cntA*
UHGG	*Proteobacteria*	*Gammaproteobacteria*	*Enterobacterales*	*Enterobacteriaceae*	*Providencia*	*Providencia rettgeri*	*MGYG-HGUT-01323*	*cntA*
UHGG	*Proteobacteria*	*Gammaproteobacteria*	*Enterobacterales*	*Enterobacteriaceae*	*Providencia*	*Providencia alcalifaciens*	*MGYG-HGUT-01465*	*cntA*
UHGG	*Proteobacteria*	*Gammaproteobacteria*	*Enterobacterales*	*Enterobacteriaceae*	*Providencia*	*Providencia rettgeri*	*MGYG-HGUT-04295*	*cntA*
UHGG	*Proteobacteria*	*Gammaproteobacteria*	*Enterobacterales*	*Enterobacteriaceae*	*Providencia*	*Providencia stuartii*	*MGYG-HGUT-01307*	*cntB*
UHGG	*Proteobacteria*	*Gammaproteobacteria*	*Enterobacterales*	*Enterobacteriaceae*	*Providencia*	*Providencia rettgeri*	*MGYG-HGUT-01323*	*cntB*
UHGG	*Proteobacteria*	*Gammaproteobacteria*	*Enterobacterales*	*Enterobacteriaceae*	*Providencia*	*Providencia alcalifaciens*	*MGYG-HGUT-01465*	*cntB*
UHGG	*Proteobacteria*	*Gammaproteobacteria*	*Enterobacterales*	*Enterobacteriaceae*	*Providencia*	*Providencia rettgeri*	*MGYG-HGUT-04295*	*cntB*
UHGG	*Proteobacteria*	*Gammaproteobacteria*	*Enterobacterales*	*Enterobacteriaceae*	*Providencia*	*Providencia rettgeri*	*MGYG-HGUT-01323*	*cutC*
UHGG	*Proteobacteria*	*Gammaproteobacteria*	*Enterobacterales*	*Enterobacteriaceae*	*Providencia*	*Providencia alcalifaciens*	*MGYG-HGUT-01465*	*cutC*
UHGG	*Proteobacteria*	*Gammaproteobacteria*	*Enterobacterales*	*Enterobacteriaceae*	*Providencia*	*Providencia alcalifaciens*	*MGYG-HGUT-01466*	*cutC*
UHGG	*Proteobacteria*	*Gammaproteobacteria*	*Enterobacterales*	*Enterobacteriaceae*	*Providencia*	*Providencia rustigianii*	*MGYG-HGUT-01708*	*cutC*
UHGG	*Proteobacteria*	*Gammaproteobacteria*	*Enterobacterales*	*Enterobacteriaceae*	*Providencia*	*Providencia rettgeri*	*MGYG-HGUT-01323*	*cutD*
UHGG	*Proteobacteria*	*Gammaproteobacteria*	*Enterobacterales*	*Enterobacteriaceae*	*Providencia*	*Providencia alcalifaciens*	*MGYG-HGUT-01465*	*cutD*
UHGG	*Proteobacteria*	*Gammaproteobacteria*	*Enterobacterales*	*Enterobacteriaceae*	*Providencia*	*Providencia alcalifaciens*	*MGYG-HGUT-01466*	*cutD*
UHGG	*Proteobacteria*	*Gammaproteobacteria*	*Enterobacterales*	*Enterobacteriaceae*	*Providencia*	*Providencia rustigianii*	*MGYG-HGUT-01708*	*cutD*
UHGG	*Proteobacteria*	*Gammaproteobacteria*	*Enterobacterales*	*Enterobacteriaceae*	*Providencia*	*Providencia rettgeri*	*MGYG-HGUT-04295*	*cutD*
UHGG	*Proteobacteria*	*Gammaproteobacteria*	*Enterobacterales*	*Enterobacteriaceae*	*Providencia*	*Providencia stuartii*	*MGYG-HGUT-01307*	*yeaW*
UHGG	*Proteobacteria*	*Gammaproteobacteria*	*Enterobacterales*	*Enterobacteriaceae*	*Providencia*	*Providencia rettgeri*	*MGYG-HGUT-01323*	*yeaW*
UHGG	*Proteobacteria*	*Gammaproteobacteria*	*Enterobacterales*	*Enterobacteriaceae*	*Providencia*	*Providencia alcalifaciens*	*MGYG-HGUT-01465*	*yeaW*
UHGG	*Proteobacteria*	*Gammaproteobacteria*	*Enterobacterales*	*Enterobacteriaceae*	*Providencia*	*Providencia rettgeri*	*MGYG-HGUT-04295*	*yeaW*
UHGG	*Proteobacteria*	*Gammaproteobacteria*	*Enterobacterales*	*Enterobacteriaceae*	*Providencia*	*Providencia stuartii*	*MGYG-HGUT-01307*	*yeaX*
UHGG	*Proteobacteria*	*Gammaproteobacteria*	*Enterobacterales*	*Enterobacteriaceae*	*Providencia*	*Providencia rettgeri*	*MGYG-HGUT-01323*	*yeaX*
UHGG	*Proteobacteria*	*Gammaproteobacteria*	*Enterobacterales*	*Enterobacteriaceae*	*Providencia*	*Providencia alcalifaciens*	*MGYG-HGUT-01465*	*yeaX*
UHGG	*Proteobacteria*	*Gammaproteobacteria*	*Enterobacterales*	*Enterobacteriaceae*	*Providencia*	*Providencia rettgeri*	*MGYG-HGUT-04295*	*yeaX*
UHGG	*Proteobacteria*	*Gammaproteobacteria*	*Enterobacterales*	*Enterobacteriaceae*	*Raoultella*	*Raoultella ornithinolytica*	*MGYG-HGUT-02363*	*cutC*
UHGG	*Proteobacteria*	*Gammaproteobacteria*	*Enterobacterales*	*Enterobacteriaceae*	*Raoultella*	*Raoultella ornithinolytica*	*MGYG-HGUT-02363*	*cutD*
UHGG	*Proteobacteria*	*Gammaproteobacteria*	*Enterobacterales*	*Enterobacteriaceae*	*Serratia*	*Serratia marcescens*	*MGYG-HGUT-02350*	*cntA*
UHGG	*Proteobacteria*	*Gammaproteobacteria*	*Enterobacterales*	*Enterobacteriaceae*	*Serratia*	*Serratia ureilytica*	*MGYG-HGUT-03399*	*cntA*
UHGG	*Proteobacteria*	*Gammaproteobacteria*	*Enterobacterales*	*Enterobacteriaceae*	*Serratia*	*Serratia liquefaciens*	*MGYG-HGUT-03712*	*cntA*
UHGG	*Proteobacteria*	*Gammaproteobacteria*	*Enterobacterales*	*Enterobacteriaceae*	*Serratia*	*Serratia marcescens*	*MGYG-HGUT-02350*	*cntB*
UHGG	*Proteobacteria*	*Gammaproteobacteria*	*Enterobacterales*	*Enterobacteriaceae*	*Serratia*	*Serratia ureilytica*	*MGYG-HGUT-03399*	*cntB*
UHGG	*Proteobacteria*	*Gammaproteobacteria*	*Enterobacterales*	*Enterobacteriaceae*	*Serratia*	*Serratia liquefaciens*	*MGYG-HGUT-03712*	*cntB*
UHGG	*Proteobacteria*	*Gammaproteobacteria*	*Enterobacterales*	*Enterobacteriaceae*	*Serratia*	*Serratia marcescens*	*MGYG-HGUT-02350*	*yeaW*
UHGG	*Proteobacteria*	*Gammaproteobacteria*	*Enterobacterales*	*Enterobacteriaceae*	*Serratia*	*Serratia ureilytica*	*MGYG-HGUT-03399*	*yeaW*
UHGG	*Proteobacteria*	*Gammaproteobacteria*	*Enterobacterales*	*Enterobacteriaceae*	*Serratia*	*Serratia liquefaciens*	*MGYG-HGUT-03712*	*yeaW*
UHGG	*Proteobacteria*	*Gammaproteobacteria*	*Enterobacterales*	*Enterobacteriaceae*	*Serratia*	*Serratia marcescens*	*MGYG-HGUT-02350*	*yeaX*
UHGG	*Proteobacteria*	*Gammaproteobacteria*	*Enterobacterales*	*Enterobacteriaceae*	*Serratia*	*Serratia ureilytica*	*MGYG-HGUT-03399*	*yeaX*
UHGG	*Proteobacteria*	*Gammaproteobacteria*	*Enterobacterales*	*Enterobacteriaceae*	*Serratia*	*Serratia liquefaciens*	*MGYG-HGUT-03712*	*yeaX*
UHGG	*Proteobacteria*	*Gammaproteobacteria*	*Enterobacterales*	*Enterobacteriaceae*	*Yersinia*	*Yersinia pestis*	*MGYG-HGUT-02476*	*cntA*
UHGG	*Proteobacteria*	*Gammaproteobacteria*	*Enterobacterales*	*Enterobacteriaceae*	*Yersinia*	*Yersinia pestis*	*MGYG-HGUT-02476*	*cntB*
UHGG	*Proteobacteria*	*Gammaproteobacteria*	*Enterobacterales*	*Enterobacteriaceae*	*Yersinia*	*Yersinia pestis*	*MGYG-HGUT-02476*	*yeaW*
UHGG	*Proteobacteria*	*Gammaproteobacteria*	*Enterobacterales*	*Enterobacteriaceae*	*Yersinia*	*Yersinia pestis*	*MGYG-HGUT-02476*	*yeaX*
UHGG	*Proteobacteria*	*Gammaproteobacteria*	*Enterobacterales*	*Enterobacteriaceae*	*Yokenella*	*Yokenella regensburgei*	*MGYG-HGUT-01394*	*cutC*
UHGG	*Proteobacteria*	*Gammaproteobacteria*	*Enterobacterales*	*Enterobacteriaceae*	*Yokenella*	*Yokenella regensburgei*	*MGYG-HGUT-01394*	*cutD*
UHGG	*Proteobacteria*	*Gammaproteobacteria*	*Pseudomonadales*	*Moraxellaceae*	*Acinetobacter*	*Acinetobacter baumannii*	*MGYG-HGUT-02344*	*cntA*
UHGG	*Proteobacteria*	*Gammaproteobacteria*	*Pseudomonadales*	*Moraxellaceae*	*Acinetobacter*	*Acinetobacter nosocomialis*	*MGYG-HGUT-02345*	*cntA*
UHGG	*Proteobacteria*	*Gammaproteobacteria*	*Pseudomonadales*	*Moraxellaceae*	*Acinetobacter*	*Acinetobacter pittii*	*MGYG-HGUT-02346*	*cntA*
UHGG	*Proteobacteria*	*Gammaproteobacteria*	*Pseudomonadales*	*Moraxellaceae*	*Acinetobacter*	*Acinetobacter baumannii*	*MGYG-HGUT-02344*	*cntB*
UHGG	*Proteobacteria*	*Gammaproteobacteria*	*Pseudomonadales*	*Moraxellaceae*	*Acinetobacter*	*Acinetobacter nosocomialis*	*MGYG-HGUT-02345*	*cntB*
UHGG	*Proteobacteria*	*Gammaproteobacteria*	*Pseudomonadales*	*Moraxellaceae*	*Acinetobacter*	*Acinetobacter pittii*	*MGYG-HGUT-02346*	*cntB*
UHGG	*Proteobacteria*	*Gammaproteobacteria*	*Pseudomonadales*	*Moraxellaceae*	*Acinetobacter*	*Acinetobacter baumannii*	*MGYG-HGUT-02344*	*yeaW*
UHGG	*Proteobacteria*	*Gammaproteobacteria*	*Pseudomonadales*	*Moraxellaceae*	*Acinetobacter*	*Acinetobacter nosocomialis*	*MGYG-HGUT-02345*	*yeaW*

UHGG	Proteobacteria	Gammaproteobacteria	Pseudomonadales	Moraxellaceae	Acinetobacter	Acinetobacter pittii	MGYG-HGUT-02346	yeaW
UHGG	Proteobacteria	Gammaproteobacteria	Pseudomonadales	Moraxellaceae	Acinetobacter	Acinetobacter baumannii	MGYG-HGUT-02344	yeaX
UHGG	Proteobacteria	Gammaproteobacteria	Pseudomonadales	Moraxellaceae	Acinetobacter	Acinetobacter nosocomialis	MGYG-HGUT-02345	yeaX
UHGG	Proteobacteria	Gammaproteobacteria	Pseudomonadales	Moraxellaceae	Acinetobacter	Acinetobacter pittii	MGYG-HGUT-02346	yeaX
UHGG	Proteobacteria	Gammaproteobacteria	Pseudomonadales	Pseudomonadaceae	Pseudomonas	Pseudomonas citronellolis	MGYG-HGUT-04151	cntA
UHGG	Proteobacteria	Gammaproteobacteria	Pseudomonadales	Pseudomonadaceae	Pseudomonas	Pseudomonas citronellolis	MGYG-HGUT-04151	cntB
UHGG	Proteobacteria	Gammaproteobacteria	Pseudomonadales	Pseudomonadaceae	Pseudomonas	Pseudomonas citronellolis	MGYG-HGUT-04151	cntB
UHGG	Proteobacteria	Gammaproteobacteria	Pseudomonadales	Pseudomonadaceae	Pseudomonas	Pseudomonas citronellolis	MGYG-HGUT-04151	yeaW
UHGG	Proteobacteria	Gammaproteobacteria	Pseudomonadales	Pseudomonadaceae	Pseudomonas	Pseudomonas citronellolis	MGYG-HGUT-04151	yeaX
SGB	Proteobacteria	Gammaproteobacteria	Enterobacterales	Morganellaceae	Providencia	Providencia_rettgeri	ZeeviD_2015__PNP_DietIntervention_31__bin.3	cntA
SGB	Proteobacteria	Gammaproteobacteria	Enterobacterales	Morganellaceae	Providencia	Providencia_rettgeri	LiJ_2014__V1.UC39-4__bin.41	cntA
SGB	Proteobacteria	Gammaproteobacteria	Enterobacterales	Enterobacteriaceae	Escherichia	Escherichia_coli	CM_madagascar__A82_02_1FE__bin.3	cntA
SGB	Proteobacteria	Gammaproteobacteria	Enterobacterales	Enterobacteriaceae	Citrobacter	Citrobacter_werkmanii	VatanenT_2016__G80513__bin.7	cntA
SGB	Proteobacteria	Gammaproteobacteria	Enterobacterales	Enterobacteriaceae	Citrobacter	Citrobacter_freundii	KarlssonFH_2013__S389__bin.5	cntA
SGB	Proteobacteria	Gammaproteobacteria	Enterobacterales	Enterobacteriaceae	Citrobacter	Citrobacter_braakii	GeversD_2014__SKBSTL011__bin.2	cntA
SGB	Proteobacteria	Gammaproteobacteria	Enterobacterales	Enterobacteriaceae	Klebsiella	Klebsiella_quasipneumoniae	QinJ_2012__DLM028__bin.4	cntA
SGB	Proteobacteria	Gammaproteobacteria	Enterobacterales	Enterobacteriaceae	Klebsiella	Klebsiella_pneumoniae	NielsenHB_2014__O2_UC38_2__bin.18	cntA
SGB	Proteobacteria	Gammaproteobacteria	Enterobacterales	Enterobacteriaceae	Klebsiella	Klebsiella_pneumoniae	VatanenT_2016__G80588__bin.5	cntA
SGB	Proteobacteria	Gammaproteobacteria	Enterobacterales	Enterobacteriaceae	Klebsiella	Klebsiella_aerogenes	VatanenT_2016__G80555__bin.3	cntA
SGB	Proteobacteria	Gammaproteobacteria	Enterobacterales	Enterobacteriaceae	Klebsiella	Klebsiella_aerogenes	VogtmannE_2016__MMRS86168210ST-27-0-0__bin.16	cntA
SGB	Proteobacteria	Gammaproteobacteria	Enterobacterales	Yersiniaceae	Serratia	Serratia_liquefaciens	VatanenT_2016__G80472__bin.3	cntA
SGB	Proteobacteria	Gammaproteobacteria	Pseudomonadales	Moraxellaceae	Acinetobacter	Acinetobacter_ursingii	BackhedF_2015__SID78_M__bin.21	cntA
SGB	Proteobacteria	Gammaproteobacteria	Xanthomonadales	Xanthomonadaceae	Stenotrophomonas	Stenotrophomonas_rhizophila	OhJ_2014__MET0293__bin.25	cntA
SGB	Proteobacteria	Gammaproteobacteria	Pseudomonadales	Pseudomonadaceae	Pseudomonas	Pseudomonas_sp_AU11447	OhJ_2014__MET0293__bin.22	cntA
SGB	Proteobacteria	Gammaproteobacteria	Pseudomonadales	Pseudomonadaceae	Pseudomonas	Pseudomonas_sp_M1	XieH_2016__YSZC12003_37197R1__bin.69	cntA
SGB	Proteobacteria	Gammaproteobacteria	Enterobacterales	Morganellaceae	Providencia	Providencia_rettgeri	ZeeviD_2015__PNP_DietIntervention_31__bin.3	cntB
SGB	Proteobacteria	Gammaproteobacteria	Enterobacterales	Morganellaceae	Providencia	Providencia_rettgeri	LiJ_2014__V1.UC39-4__bin.41	cntB
SGB	Proteobacteria	Gammaproteobacteria	Enterobacterales	Enterobacteriaceae	Escherichia	Escherichia_albertii	BackhedF_2015__SID105_12M__bin.10	cntB
SGB	Proteobacteria	Gammaproteobacteria	Enterobacterales	Enterobacteriaceae	Escherichia	Escherichia_coli	CM_madagascar__A82_02_1FE__bin.3	cntB
SGB	Proteobacteria	Gammaproteobacteria	Enterobacterales	Enterobacteriaceae	Citrobacter	Citrobacter_werkmanii	VatanenT_2016__G80513__bin.7	cntB
SGB	Proteobacteria	Gammaproteobacteria	Enterobacterales	Enterobacteriaceae	Citrobacter	Citrobacter_freundii	KarlssonFH_2013__S389__bin.5	cntB
SGB	Proteobacteria	Gammaproteobacteria	Enterobacterales	Enterobacteriaceae	Citrobacter	Citrobacter_braakii	GeversD_2014__SKBSTL011__bin.2	cntB
SGB	Proteobacteria	Gammaproteobacteria	Enterobacterales	Enterobacteriaceae	Klebsiella	Klebsiella_quasipneumoniae	QinJ_2012__DLM028__bin.4	cntB
SGB	Proteobacteria	Gammaproteobacteria	Enterobacterales	Enterobacteriaceae	Klebsiella	Klebsiella_pneumoniae	NielsenHB_2014__O2_UC38_2__bin.18	cntB
SGB	Proteobacteria	Gammaproteobacteria	Enterobacterales	Enterobacteriaceae	Klebsiella	Klebsiella_pneumoniae	VatanenT_2016__G80588__bin.5	cntB
SGB	Proteobacteria	Gammaproteobacteria	Enterobacterales	Enterobacteriaceae	Klebsiella	Klebsiella_aerogenes	VatanenT_2016__G80555__bin.3	cntB
SGB	Proteobacteria	Gammaproteobacteria	Enterobacterales	Enterobacteriaceae	Klebsiella	Klebsiella_aerogenes	VogtmannE_2016__MMRS86168210ST-27-0-0__bin.16	cntB
SGB	Proteobacteria	Gammaproteobacteria	Enterobacterales	Yersiniaceae	Serratia	Serratia_liquefaciens	VatanenT_2016__G80472__bin.3	cntB
SGB	Proteobacteria	Gammaproteobacteria	Pseudomonadales	Moraxellaceae	Acinetobacter	Acinetobacter_baumannii	WenC_2017__RA130__bin.22	cntB
SGB	Proteobacteria	Gammaproteobacteria	Pseudomonadales	Moraxellaceae	Acinetobacter	Acinetobacter_ursingii	BackhedF_2015__SID78_M__bin.21	cntB
SGB	Proteobacteria	Gammaproteobacteria	Xanthomonadales	Xanthomonadaceae	Stenotrophomonas	Stenotrophomonas_rhizophila	OhJ_2014__MET0293__bin.25	cntB
SGB	Proteobacteria	Gammaproteobacteria	Pseudomonadales	Pseudomonadaceae	Pseudomonas	Pseudomonas_sp_AU11447	OhJ_2014__MET0293__bin.22	cntB
SGB	Proteobacteria	Gammaproteobacteria	Pseudomonadales	Pseudomonadaceae	Pseudomonas	Pseudomonas_sp_AU11447	OhJ_2014__MET0293__bin.22	cntB
SGB	Proteobacteria	Gammaproteobacteria	Pseudomonadales	Pseudomonadaceae	Pseudomonas	Pseudomonas_sp_T	OhJ_2014__MET0241__bin.25	cntB
SGB	Proteobacteria	Gammaproteobacteria	Pseudomonadales	Pseudomonadaceae	Pseudomonas	Pseudomonas_sp_M1	XieH_2016__YSZC12003_37197R1__bin.69	cntB
SGB	Proteobacteria	Gammaproteobacteria	Pseudomonadales	Pseudomonadaceae	Pseudomonas	Pseudomonas_sp_M1	XieH_2016__YSZC12003_37197R1__bin.69	cntB
SGB	Actinobacteria	Coriobacteriia	Coriobacteriales	Atopobiaceae	Atopobium	Atopobium_minutum	YuJ_2015__SZAXPI003412-1__bin.19	cutC
SGB	Firmicutes	Clostridia	Clostridiales	Clostridiales_Family_XIII_In	Mogibacterium	Mogibacterium_sp_CM50	CM_periimplantitis__SP_305SPI_T016__bin.15	cutC
SGB	Bacteroidetes	Bacteroidia	Bacteroidales	Bacteroidaceae	Bacteroides	Bacteroides_vulgatus	ZeeviD_2015__PNP_Main_62__bin.36	cutC
SGB	Firmicutes	Clostridia	Clostridiales	Peptococcaceae	Peptococcus	Peptococcus_niger	VincentC_2016__MM058.1__bin.7	cutC
SGB	Firmicutes	Bacilli	Lactobacillales	Enterococcaceae	Enterococcus	Enterococcus_faecium	FengQ_2015__SID31477__bin.63	cutC
SGB	Firmicutes	Bacilli	Lactobacillales	Lactobacillaceae	Lactobacillus	Lactobacillus_casei	BackhedF_2015__SID184_4M__bin.17	cutC
SGB	Firmicutes	Clostridia	Clostridiales	Clostridiales_unclassified	Flavonifractor	Flavonifractor_sp_An10	XieH_2016__YSZC12003_36116__bin.21	cutC
SGB	Firmicutes	Clostridia	Clostridiales	Peptococcaceae	Peptococcus	Peptococcus_niger	BackhedF_2015__SID335_M__bin.63	cutC
SGB	Firmicutes	Clostridia	Clostridiales	Lachnospiraceae	Blautia	Blautia_obeum	Bengtsson-PalmeJ_2015__TRAVELRES46__bin.63	cutC
SGB	Firmicutes	Clostridia	Clostridiales	Lachnospiraceae	Johnsonella	Johnsonella_ignava	HMP_2012__SRS019125__bin.20	cutC
SGB	Firmicutes	Clostridia	Clostridiales	Lachnospiraceae	Lachnoclostridium	Clostridium_asparagiforme	HeQ_2017__RSZAXPI003069-101__bin.6	cutC
SGB	Firmicutes	Clostridia	Clostridiales	Lachnospiraceae	Lachnoclostridium	Clostridium_lavalense	HeQ_2017__RSZAXPI003099-133__bin.12	cutC
SGB	Firmicutes	Clostridia	Clostridiales	Lachnospiraceae	Lachnoclostridium	Clostridium_saccharolyticum	GeversD_2014__SKBSTL023__bin.2	cutC
SGB	Firmicutes	Clostridia	Clostridiales	Clostridiaceae	Hungatella	Hungatella_hathewayi	NielsenHB_2014__V1_UC14_1__bin.35	cutC
SGB	Firmicutes	Clostridia	Clostridiales	Clostridiaceae	Hungatella	Hungatella_hathewayi	HeQ_2017__RSZAXPI003077-109__bin.16	cutC
SGB	Firmicutes	Negativicutes	Veillonellales	Veillonellaceae	Megasphaera	Megasphaera_sp_DJF_B143	ZellerG_2014__CCIS11362406ST-4-0__bin.75	cutC
SGB	Firmicutes	Negativicutes	Veillonellales	Veillonellaceae	Megasphaera	Megasphaera_sp_DJF_B143	HeQ_2017__SZAXPI029575-90__bin.40	cutC
SGB	Firmicutes	Negativicutes	Veillonellales	Veillonellaceae	Megasphaera	Megasphaera_sp_DJF_B143	HeQ_2017__SZAXPI029575-90__bin.40	cutC
SGB	Firmicutes	Negativicutes	Veillonellales	Veillonellaceae	Megasphaera	Megasphaera_micronuciformis	BackhedF_2015__SID565_4M__bin.4	cutC
SGB	Fusobacteria	Fusobacteriia	Fusobacteriales	Fusobacteriaceae	Cetobacterium	Cetobacterium_somerae	YuJ_2015__SZAXPI015255-46__bin.49	cutC
SGB	Fusobacteria	Fusobacteriia	Fusobacteriales	Fusobacteriaceae	Fusobacterium	Fusobacterium_varium	QinN_2014__HD-78__bin.12	cutC
SGB	Firmicutes	Clostridia	Clostridiales	Clostridiaceae	Clostridium	Clostridium_disporicum	FerrettiP_2018__CA_C10020IS2336FE_t1M15__bin.8	cutC
SGB	Firmicutes	Clostridia	Clostridiales	Clostridiaceae	Clostridium	Clostridium_paraputrificum	FerrettiP_2018__CA_C10003IS2040FE_t3M15__bin.2	cutC
SGB	Firmicutes	Clostridia	Clostridiales	Clostridiaceae	Clostridium	Clostridium_sporogenes	BackhedF_2015__SID615_4M__bin.18	cutC
SGB	Firmicutes	Clostridia	Clostridiales	Clostridiaceae	Clostridium	Clostridium_sulfidigenes	HeQ_2017__RSZAXPI003063-104__bin.17	cutC
SGB	Firmicutes	Clostridia	Clostridiales	Peptostreptococcaceae	Peptoanaerobacter	Eubacterium_yurii	HMP_2012__SRS022725__bin.23	cutC
SGB	Firmicutes	Clostridia	Clostridiales	Peptostreptococcaceae	Peptoanaerobacter	Eubacterium_yurii	HMP_2012__SRS015215__bin.44	cutC
SGB	Firmicutes	Tissierellia	Tissierellales	Peptoniphilaceae	Anaerococcus	Anaerococcus_hydrogenalis	VincentC_2016__MM081__bin.7	cutC
SGB	Firmicutes	Tissierellia	Tissierellales	Peptoniphilaceae	Anaerococcus	Anaerococcus_lactolyticus	VincentC_2016__MM004.2__bin.5	cutC
SGB	Firmicutes	Tissierellia	Tissierellales	Peptoniphilaceae	Anaerococcus	Anaerococcus_tetradius	HMP_2012__SRS011111__bin.13	cutC
SGB	Firmicutes	Negativicutes	Veillonellales	Veillonellaceae	Veillonella	Veillonella_seminalis	YuJ_2015__SZAXPI003434-7__bin.67	cutC
SGB	Firmicutes	Bacilli	Lactobacillales	Enterococcaceae	Vagococcus	Vagococcus_teuberi	HeQ_2017__SZAXPI029501-104__bin.19	cutC
SGB	Firmicutes	Bacilli	Lactobacillales	Enterococcaceae	Enterococcus	Enterococcus_raffinosus	BackhedF_2015__SID1_4M__bin.4	cutC
SGB	Firmicutes	Bacilli	Lactobacillales	Enterococcaceae	Enterococcus	Enterococcus_sp_HMSC05C03	FerrettiP_2018__CA_C10008IS2131FE_t5M16__bin.7	cutC
SGB	Firmicutes	Bacilli	Lactobacillales	Enterococcaceae	Enterococcus	Enterococcus_dispar	FengQ_2015__SID31870__bin.16	cutC
SGB	Proteobacteria	Gammaproteobacteria	Enterobacterales	Morganellaceae	Proteus	Proteus_mirabilis	HeQ_2017__SZAXPI029574-89__bin.4	cutC
SGB	Proteobacteria	Gammaproteobacteria	Enterobacterales	Morganellaceae	Proteus	Proteus_vulgaris	HeQ_2017__SZAXPI029545-129__bin.16	cutC
SGB	Proteobacteria	Gammaproteobacteria	Enterobacterales	Morganellaceae	Providencia	Providencia_rettgeri	ZeeviD_2015__PNP_DietIntervention_31__bin.3	cutC
SGB	Proteobacteria	Gammaproteobacteria	Enterobacterales	Morganellaceae	Providencia	Providencia_rettgeri	LiJ_2014__V1.UC39-4__bin.41	cutC
SGB	Proteobacteria	Gammaproteobacteria	Enterobacterales	Enterobacteriaceae	Klebsiella	Klebsiella_quasipneumoniae	QinJ_2012__DLM028__bin.4	cutC
SGB	Proteobacteria	Gammaproteobacteria	Enterobacterales	Enterobacteriaceae	Klebsiella	Klebsiella_pneumoniae	NielsenHB_2014__O2_UC38_2__bin.18	cutC
SGB	Proteobacteria	Gammaproteobacteria	Enterobacterales	Enterobacteriaceae	Klebsiella	Klebsiella_aerogenes	VatanenT_2016__G80555__bin.3	cutC
SGB	Proteobacteria	Gammaproteobacteria	Enterobacterales	Enterobacteriaceae	Klebsiella	Klebsiella_aerogenes	VogtmannE_2016__MMRS86168210ST-27-0-0__bin.16	cutC
SGB	Proteobacteria	Gammaproteobacteria	Enterobacterales	Enterobacteriaceae	Klebsiella	Klebsiella_oxytoca	FengQ_2015__SID531155__bin.23	cutC
SGB	Proteobacteria	Gammaproteobacteria	Enterobacterales	Enterobacteriaceae	Klebsiella	Klebsiella_michiganensis	BackhedF_2015__SID608_B__bin.1	cutC
SGB	Proteobacteria	Gammaproteobacteria	Enterobacterales	Enterobacteriaceae	Klebsiella	Klebsiella_sp_Kd70_TUC_EEAOC	FerrettiP_2018__CA_C10035IS2913FE_t4M16__bin.3	cutC
SGB	Proteobacteria	Gammaproteobacteria	Enterobacterales	Enterobacteriaceae	Raoultella	Raoultella_ornithinolytica	FerrettiP_2018__CA_C10029IS2503FE_t3M15__bin.8	cutC
SGB	Actinobacteria	Coriobacteriia	Coriobacteriales	Atopobiaceae	Olsenella	Olsenella_uli	CM_periimplantitis__SP_143SPI_T016__bin.9	cutC
SGB	Actinobacteria	Coriobacteriia	Coriobacteriales	Atopobiaceae	Olsenella	Olsenella_umbonata	BritoIL_2016__W2.9.ST__bin.18	cutC
SGB	Actinobacteria	Coriobacteriia	Coriobacteriales	Atopobiaceae	Olsenella	Olsenella_umbonata	QinJ_2012__T2D-103__bin.15	cutC
SGB	Actinobacteria	Coriobacteriia	Coriobacteriales	Coriobacteriaceae	Collinsella	Collinsella_aerofaciens	ZellerG_2014__CCIS78100604ST-4-0__bin.15	cutC
SGB	Firmicutes	Clostridia	Clostridiales	Ruminococcaceae	Ruminiclostridium	Clostridium_leptum	FengQ_2015__SID31455__bin.4	cutC
SGB	Firmicutes	Clostridia	Clostridiales	Ruminococcaceae	Gemmiger	Gemmiger_sp_An194	LiJ_2014__O2.UC48-1__bin.38	cutC
SGB	Proteobacteria	Deltaproteobacteria	Desulfovibrionales	Desulfovibrionaceae	Desulfovibrio	Desulfovibrio_piger	ZellerG_2014__CCMD50071957ST-21-0__bin.42	cutC
SGB	Proteobacteria	Deltaproteobacteria	Desulfovibrionales	Desulfovibrionaceae	Desulfovibrio	Desulfovibrio_piger	LiJ_2014__MH0398__bin.77	cutC
SGB	Proteobacteria	Deltaproteobacteria	Desulfovibrionales	Desulfovibrionaceae	Bilophila	Bilophila_sp_4_1_30	LeChatelierE_2013__MH0239__bin.24	cutC
SGB	Proteobacteria	Deltaproteobacteria	Desulfovibrionales	Desulfovibrionaceae	Bilophila	Bilophila_wadsworthia	FerrettiP_2018__CA_C10024IS2415FE_t3M15__bin.18	cutC
SGB	Proteobacteria	Deltaproteobacteria	Desulfovibrionales	Desulfovibrionaceae	Bilophila	Bilophila_wadsworthia	LiJ_2017__H1M412837__bin.49	cutC
SGB	Proteobacteria	Deltaproteobacteria	Desulfovibrionales	Desulfovibrionaceae	Bilophila	Bilophila_sp_4_1_30	HeQ_2017__SZAXPI029557-42__bin.30	cutC
SGB	Proteobacteria	Deltaproteobacteria	Desulfovibrionales	Desulfovibrionaceae	Desulfovibrio	Desulfovibrio_piger	LeChatelierE_2013__MH0108__bin.24	cutC
SGB	Proteobacteria	Deltaproteobacteria	Desulfovibrionales	Desulfovibrionaceae	Desulfovibrio	Desulfovibrio_desulfuricans	LouisS_2016__AS66_18__bin.13	cutC
SGB	Proteobacteria	Deltaproteobacteria	Desulfovibrionales	Desulfovibrionaceae	Desulfovibrio	Desulfovibrio_desulfuricans	XieH_2016__YSZC12003_35387__bin.50	cutC
SGB	Proteobacteria	Deltaproteobacteria	Desulfovibrionales	Desulfovibrionaceae	Desulfovibrio	Desulfovibrio_desulfuricans	QinN_2014__LD-22__bin.30	cutC
SGB	Proteobacteria	Deltaproteobacteria	Desulfovibrionales	Desulfovibrionaceae	Desulfovibrio	Desulfovibrio_piger	RaymondF_2016__P1E0__bin.62	cutC
SGB	Proteobacteria	Deltaproteobacteria	Desulfovibrionales	Desulfovibrionaceae	Desulfovibrio	Desulfovibrio_legallii	BackhedF_2015__SID624_M__bin.2	cutC
SGB	Firmicutes	Bacilli	Lactobacillales	Enterococcaceae	Enterococcus	Enterococcus_gallinarum	VatanenT_2016__G78762__bin.2	cutD
SGB	Actinobacteria	Coriobacteriia	Coriobacteriales	Atopobiaceae	Atopobium	Atopobium_minutum	YuJ_2015__SZAXPI003412-1__bin.19	cutD
SGB	Bacteroidetes	Bacteroidia	Bacteroidales	Bacteroidaceae	Bacteroides	Bacteroides_xylanisolvens	HMP_2012__SRS042628__bin.29	cutD
SGB	Firmicutes	Clostridia	Clostridiales	Clostridiales_Family_XIII_In	Mogibacterium	Mogibacterium_sp_CM50	CM_periimplantitis__SP_305SPI_T016__bin.15	cutD
SGB	Bacteroidetes	Bacteroidia	Bacteroidales	Bacteroidaceae	Bacteroides	Bacteroides_vulgatus	ZeeviD_2015__PNP_Main_62__bin.36	cutD
SGB	Firmicutes	Clostridia	Clostridiales	Peptococcaceae	Peptococcus	Peptococcus_niger	VincentC_2016__MM058.1__bin.7	cutD
SGB	Firmicutes	Bacilli	Lactobacillales	Enterococcaceae	Enterococcus	Enterococcus_faecium	FengQ_2015__SID31477__bin.63	cutD
SGB	Firmicutes	Bacilli	Lactobacillales	Lactobacillaceae	Lactobacillus	Lactobacillus_casei	BackhedF_2015__SID184_4M__bin.17	cutD
SGB	Firmicutes	Clostridia	Clostridiales	Clostridiales_unclassified	Flavonifractor	Flavonifractor_sp_An10	XieH_2016__YSZC12003_36116__bin.21	cutD
SGB	Firmicutes	Clostridia	Clostridiales	Peptococcaceae	Peptococcus	Peptococcus_niger	BackhedF_2015__SID335_M__bin.63	cutD
SGB	Firmicutes	Clostridia	Clostridiales	Lachnospiraceae	Blautia	Blautia_obeum	Bengtsson-PalmeJ_2015__TRAVELRES46__bin.63	cutD
SGB	Firmicutes	Clostridia	Clostridiales	Lachnospiraceae	Roseburia	Roseburia_faecis	CM_madagascar__A09_02_1FE__bin.63	cutD
SGB	Firmicutes	Clostridia	Clostridiales	Lachnospiraceae	Johnsonella	Johnsonella_ignava	HMP_2012__SRS019125__bin.20	cutD
SGB	Firmicutes	Clostridia	Clostridiales	Lachnospiraceae	Lachnoclostridium	Clostridium_asparagiforme	HeQ_2017__RSZAXPI003069-101__bin.6	cutD
SGB	Firmicutes	Clostridia	Clostridiales	Lachnospiraceae	Lachnoclostridium	Clostridium_lavalense	HeQ_2017__RSZAXPI003099-133__bin.12	cutD
SGB	Firmicutes	Clostridia	Clostridiales	Lachnospiraceae	Lachnoclostridium	Clostridium_saccharolyticum	GeversD_2014__SKBSTL023__bin.2	cutD
SGB	Firmicutes	Clostridia	Clostridiales	Clostridiaceae	Hungatella	Hungatella_hathewayi	NielsenHB_2014__V1_UC14_1__bin.35	cutD
SGB	Firmicutes	Clostridia	Clostridiales	Clostridiaceae	Hungatella	Hungatella_hathewayi	HeQ_2017__RSZAXPI003077-109__bin.16	cutD
SGB	Firmicutes	Negativicutes	Veillonellales	Veillonellaceae	Megasphaera	Megasphaera_sp_DJF_B143	ZellerG_2014__CCIS11362406ST-4-0__bin.75	cutD
SGB	Firmicutes	Negativicutes	Veillonellales	Veillonellaceae	Megasphaera	Megasphaera_sp_DJF_B143	HeQ_2017__SZAXPI029575-90__bin.40	cutD
SGB	Firmicutes	Negativicutes	Veillonellales	Veillonellaceae	Megasphaera	Megasphaera_sp_NM10	BritoIL_2016__M1.36.ST__bin.16	cutD
SGB	Firmicutes	Negativicutes	Veillonellales	Veillonellaceae	Megasphaera	Megasphaera_elsdenii	VincentC_2016__MM027.2__bin.14	cutD
SGB	Firmicutes	Negativicutes	Veillonellales	Veillonellaceae	Megasphaera	Megasphaera_elsdenii	CosteaPI_2017__SID713A030-11-0-0__bin.42	cutD
SGB	Firmicutes	Negativicutes	Veillonellales	Veillonellaceae	Megasphaera	Megasphaera_micronuciformis	BackhedF_2015__SID565_4M__bin.4	cutD
SGB	Fusobacteria	Fusobacteriia	Fusobacteriales	Fusobacteriaceae	Cetobacterium	Cetobacterium_somerae	YuJ_2015__SZAXPI015255-46__bin.49	cutD
SGB	Firmicutes	Clostridia	Clostridiales	Clostridiaceae	Clostridium	Clostridium_disporicum	FerrettiP_2018__CA_C10020IS2336FE_t1M15__bin.8	cutD
SGB	Firmicutes	Clostridia	Clostridiales	Clostridiaceae	Clostridium	Clostridium_paraputrificum	FerrettiP_2018__CA_C10003IS2040FE_t3M15__bin.2	cutD
SGB	Firmicutes	Clostridia	Clostridiales	Clostridiaceae	Clostridium	Clostridium_sulfidigenes	HeQ_2017__RSZAXPI003063-104__bin.17	cutD
SGB	Firmicutes	Clostridia	Clostridiales	Peptostreptococcaceae	Peptoanaerobacter	Eubacterium_yurii	HMP_2012__SRS017209__bin.22	cutD
SGB	Firmicutes	Clostridia	Clostridiales	Peptostreptococcaceae	Peptoanaerobacter	Eubacterium_yurii	HMP_2012__SRS022725__bin.23	cutD
SGB	Firmicutes	Clostridia	Clostridiales	Peptostreptococcaceae	Peptoanaerobacter	Eubacterium_yurii	HMP_2012__SRS015215__bin.44	cutD
SGB	Firmicutes	Clostridia	Clostridiales	Peptostreptococcaceae	Peptoanaerobacter	Peptoanaerobacter_stomatis	HMP_2012__SRS015797__bin.58	cutD
SGB	Firmicutes	Tissierellia	Tissierellales	Peptoniphilaceae	Anaerococcus	Anaerococcus_sp_HMSC065G05	VincentC_2016__MM003.2__bin.16	cutD
SGB	Firmicutes	Tissierellia	Tissierellales	Peptoniphilaceae	Anaerococcus	Anaerococcus_hydrogenalis	VincentC_2016__MM081__bin.7	cutD
SGB	Firmicutes	Tissierellia	Tissierellales	Peptoniphilaceae	Anaerococcus	Anaerococcus_lactolyticus	VincentC_2016__MM004.2__bin.5	cutD
SGB	Firmicutes	Tissierellia	Tissierellales	Peptoniphilaceae	Anaerococcus	Anaerococcus_tetradius	HMP_2012__SRS011111__bin.13	cutD
SGB	Firmicutes	Negativicutes	Veillonellales	Veillonellaceae	Veillonella	Veillonella_seminalis	YuJ_2015__SZAXPI003434-7__bin.67	cutD
SGB	Firmicutes	Bacilli	Lactobacillales	Enterococcaceae	Vagococcus	Vagococcus_teuberi	HeQ_2017__SZAXPI029501-104__bin.19	cutD
SGB	Firmicutes	Bacilli	Lactobacillales	Enterococcaceae	Enterococcus	Enterococcus_raffinosus	BackhedF_2015__SID1_4M__bin.4	cutD
SGB	Firmicutes	Bacilli	Lactobacillales	Enterococcaceae	Enterococcus	Enterococcus_sp_HMSC05C03	FerrettiP_2018__CA_C10008IS2131FE_t5M16__bin.7	cutD
SGB	Firmicutes	Bacilli	Lactobacillales	Enterococcaceae	Enterococcus	Enterococcus_dispar	FengQ_2015__SID31870__bin.16	cutD
SGB	Verrucomicrobia	Verrucomicrobiae	Verrucomicrobiales	Verrucomicrobiaceae	Verrucomicrobium	Verrucomicrobium_sp_GAS474	BritoIL_2016__M1.1.ST__bin.106	cutD
SGB	Verrucomicrobia	Verrucomicrobiae	Verrucomicrobiales	Akkermansiaceae	Akkermansia	Akkermansia_glycaniphila	CM_madagascar__A44_01_1FE__bin.13	cutD
SGB	Proteobacteria	Gammaproteobacteria	Enterobacterales	Morganellaceae	Proteus	Proteus_mirabilis	HeQ_2017__SZAXPI029574-89__bin.4	cutD
SGB	Proteobacteria	Gammaproteobacteria	Enterobacterales	Morganellaceae	Proteus	Proteus_vulgaris	HeQ_2017__SZAXPI029545-129__bin.16	cutD
SGB	Proteobacteria	Gammaproteobacteria	Enterobacterales	Morganellaceae	Providencia	Providencia_rettgeri	ZeeviD_2015__PNP_DietIntervention_31__bin.3	cutD
SGB	Proteobacteria	Gammaproteobacteria	Enterobacterales	Morganellaceae	Providencia	Providencia_rettgeri	LiJ_2014__V1.UC39-4__bin.41	cutD
SGB	Proteobacteria	Gammaproteobacteria	Enterobacterales	Enterobacteriaceae	Kluyvera	Kluyvera_ascorbata	LiJ_2017__H2M513938__bin.79	cutD
SGB	Proteobacteria	Gammaproteobacteria	Enterobacterales	Enterobacteriaceae	Klebsiella	Klebsiella_quasipneumoniae	QinJ_2012__DLM028__bin.4	cutD
SGB	Proteobacteria	Gammaproteobacteria	Enterobacterales	Enterobacteriaceae	Klebsiella	Klebsiella_pneumoniae	NielsenHB_2014__O2_UC38_2__bin.18	cutD
SGB	Proteobacteria	Gammaproteobacteria	Enterobacterales	Enterobacteriaceae	Klebsiella	Klebsiella_aerogenes	VatanenT_2016__G80555__bin.3	cutD
SGB	Proteobacteria	Gammaproteobacteria	Enterobacterales	Enterobacteriaceae	Klebsiella	Klebsiella_aerogenes	VogtmannE_2016__MMRS86168210ST-27-0-0__bin.16	cutD
SGB	Proteobacteria	Gammaproteobacteria	Enterobacterales	Enterobacteriaceae	Klebsiella	Klebsiella_oxytoca	FengQ_2015__SID531155__bin.23	cutD
SGB	Proteobacteria	Gammaproteobacteria	Enterobacterales	Enterobacteriaceae	Klebsiella	Klebsiella_michiganensis	BackhedF_2015__SID608_B__bin.1	cutD

SGB	Proteobacteria	Gammaproteobacteria	Enterobacterales	Enterobacteriaceae	Klebsiella	Klebsiella_sp_Kd70_TUC_EEAOC	FerrettiP_2018__CA_C10035IS2913FE_t4M16__bin.3	cutD
SGB	Proteobacteria	Gammaproteobacteria	Enterobacterales	Enterobacteriaceae	Raoultella	Raoultella_ornithinolytica	FerrettiP_2018__CA_C10029IS2503FE_t3M15__bin.8	cutD
SGB	Firmicutes	Clostridia	Clostridiales	Clostridiaceae	Clostridium	Clostridium_sp_ATCC_29733	XieH_2016__YSZC12003_36055__bin.93	cutD
SGB	Firmicutes	Clostridia	Clostridiales	Lachnospiraceae	Blautia	Ruminococcus_sp	XieH_2016__YSZC12003_37394__bin.53	cutD
SGB	Firmicutes	Clostridia	Clostridiales	Ruminococcaceae	Ruminiclostridium	Eubacterium_siraeum	Obregon-TitoAJ_2015__SM20__bin.35	cutD
SGB	Actinobacteria	Coriobacteriia	Coriobacteriales	Atopobiaceae	Olsenella	Olsenella_uli	CM_periimplantitis__SP_143SPI_T016__bin.9	cutD
SGB	Actinobacteria	Coriobacteriia	Coriobacteriales	Atopobiaceae	Olsenella	Olsenella_umbonata	BritoIL_2016__W2.9.ST__bin.18	cutD
SGB	Actinobacteria	Coriobacteriia	Coriobacteriales	Atopobiaceae	Olsenella	Olsenella_umbonata	QinJ_2012__T2D-103__bin.15	cutD
SGB	Actinobacteria	Coriobacteriia	Coriobacteriales	Coriobacteriaceae	Collinsella	Collinsella_aerofaciens	ZellerG_2014__CCIS78100604ST-4-0__bin.15	cutD
SGB	Firmicutes	Clostridia	Clostridiales	Ruminococcaceae	Ruminiclostridium	Clostridium_leptum	FengQ_2015__SID31455__bin.4	cutD
SGB	Firmicutes	Clostridia	Clostridiales	Ruminococcaceae	Anaeromassilibacillus	Anaeromassilibacillus_sp_An250	LoombaR_2017__SID1050_bax__bin.11	cutD
SGB	Firmicutes	Clostridia	Clostridiales	Ruminococcaceae	Anaeromassilibacillus	Anaeromassilibacillus_sp_An250	LiJ_2014__MH0370__bin.9	cutD
SGB	Firmicutes	Clostridia	Clostridiales	Ruminococcaceae	Faecalibacterium	Faecalibacterium_prausnitzii	LiJ_2014__V1.UC58-4__bin.44	cutD
SGB	Firmicutes	Clostridia	Clostridiales	Ruminococcaceae	Gemmiger	Gemmiger_sp_An194	LiJ_2014__O2.UC48-1__bin.38	cutD
SGB	Proteobacteria	Deltaproteobacteria	Desulfovibrionales	Desulfovibrionaceae	Desulfovibrio	Desulfovibrio_piger	ZellerG_2014__CCMD50071957ST-21-0__bin.42	cutD
SGB	Proteobacteria	Deltaproteobacteria	Desulfovibrionales	Desulfovibrionaceae	Desulfovibrio	Desulfovibrio_piger	LiJ_2014__MH0398__bin.77	cutD
SGB	Proteobacteria	Deltaproteobacteria	Desulfovibrionales	Desulfovibrionaceae	Bilophila	Bilophila_sp_4_1_30	LeChatelierE_2013__MH0239__bin.24	cutD
SGB	Proteobacteria	Deltaproteobacteria	Desulfovibrionales	Desulfovibrionaceae	Bilophila	Bilophila_wadsworthia	FerrettiP_2018__CA_C10024IS2415FE_t3M15__bin.18	cutD
SGB	Proteobacteria	Deltaproteobacteria	Desulfovibrionales	Desulfovibrionaceae	Bilophila	Bilophila_wadsworthia	LiJ_2017__H1M412837__bin.49	cutD
SGB	Proteobacteria	Deltaproteobacteria	Desulfovibrionales	Desulfovibrionaceae	Bilophila	Bilophila_sp_4_1_30	HeQ_2017__SZAXPI029557-42__bin.30	cutD
SGB	Proteobacteria	Deltaproteobacteria	Desulfovibrionales	Desulfovibrionaceae	Desulfovibrio	Desulfovibrio_piger	ChengpingW_2017__AS9raw__bin.35	cutD
SGB	Proteobacteria	Deltaproteobacteria	Desulfovibrionales	Desulfovibrionaceae	Desulfovibrio	Desulfovibrio_piger	LeChatelierE_2013__MH0108__bin.24	cutD
SGB	Proteobacteria	Deltaproteobacteria	Desulfovibrionales	Desulfovibrionaceae	Desulfovibrio	Desulfovibrio_desulfuricans	LouisS_2016__AS66_18__bin.13	cutD
SGB	Proteobacteria	Deltaproteobacteria	Desulfovibrionales	Desulfovibrionaceae	Desulfovibrio	Desulfovibrio_desulfuricans	XieH_2016__YSZC12003_35387__bin.50	cutD
SGB	Proteobacteria	Deltaproteobacteria	Desulfovibrionales	Desulfovibrionaceae	Desulfovibrio	Desulfovibrio_desulfuricans	QinN_2014__LD-22__bin.30	cutD
SGB	Proteobacteria	Deltaproteobacteria	Desulfovibrionales	Desulfovibrionaceae	Desulfovibrio	Desulfovibrio_piger	RaymondF_2016__P1E0__bin.62	cutD
SGB	Proteobacteria	Deltaproteobacteria	Desulfovibrionales	Desulfovibrionaceae	Desulfovibrio	Desulfovibrio_legallii	BackhedF_2015__SID624_M__bin.2	cutD
SGB	Proteobacteria	Gammaproteobacteria	Enterobacterales	Morganellaceae	Providencia	Providencia_rettgeri	ZeeviD_2015__PNP_DietIntervention_31__bin.3	yeaW
SGB	Proteobacteria	Gammaproteobacteria	Enterobacterales	Morganellaceae	Providencia	Providencia_rettgeri	LiJ_2014__V1.UC39-4__bin.41	yeaW
SGB	Proteobacteria	Gammaproteobacteria	Enterobacterales	Enterobacteriaceae	Escherichia	Escherichia_coli	CM_madagascar__A82_02_1FE__bin.3	yeaW
SGB	Proteobacteria	Gammaproteobacteria	Enterobacterales	Enterobacteriaceae	Citrobacter	Citrobacter_werkmanii	VatanenT_2016__G80513__bin.7	yeaW
SGB	Proteobacteria	Gammaproteobacteria	Enterobacterales	Enterobacteriaceae	Citrobacter	Citrobacter_freundii	KarlssonFH_2013__S389__bin.5	yeaW
SGB	Proteobacteria	Gammaproteobacteria	Enterobacterales	Enterobacteriaceae	Citrobacter	Citrobacter_braakii	GeversD_2014__SKBSTL011__bin.2	yeaW
SGB	Proteobacteria	Gammaproteobacteria	Enterobacterales	Enterobacteriaceae	Klebsiella	Klebsiella_quasipneumoniae	QinJ_2012__DLM028__bin.4	yeaW
SGB	Proteobacteria	Gammaproteobacteria	Enterobacterales	Enterobacteriaceae	Klebsiella	Klebsiella_pneumoniae	NielsenHB_2014__O2_UC38_2__bin.18	yeaW
SGB	Proteobacteria	Gammaproteobacteria	Enterobacterales	Enterobacteriaceae	Klebsiella	Klebsiella_pneumoniae	VatanenT_2016__G80588__bin.5	yeaW
SGB	Proteobacteria	Gammaproteobacteria	Enterobacterales	Enterobacteriaceae	Klebsiella	Klebsiella_aerogenes	VatanenT_2016__G80555__bin.3	yeaW
SGB	Proteobacteria	Gammaproteobacteria	Enterobacterales	Enterobacteriaceae	Klebsiella	Klebsiella_aerogenes	VogtmannE_2016__MMRS86168210ST-27-0-0__bin.16	yeaW
SGB	Proteobacteria	Gammaproteobacteria	Enterobacterales	Yersiniaceae	Serratia	Serratia_liquefaciens	VatanenT_2016__G80472__bin.3	yeaW
SGB	Proteobacteria	Gammaproteobacteria	Pseudomonadales	Moraxellaceae	Acinetobacter	Acinetobacter_ursingii	BackhedF_2015__SID78_M__bin.21	yeaW
SGB	Proteobacteria	Gammaproteobacteria	Xanthomonadales	Xanthomonadaceae	Stenotrophomonas	Stenotrophomonas_rhizophila	OhJ_2014__MET0293__bin.25	yeaW
SGB	Proteobacteria	Gammaproteobacteria	Pseudomonadales	Pseudomonadaceae	Pseudomonas	Pseudomonas_sp_AU11447	OhJ_2014__MET0293__bin.22	yeaW
SGB	Proteobacteria	Gammaproteobacteria	Pseudomonadales	Pseudomonadaceae	Pseudomonas	Pseudomonas_sp_M1	XieH_2016__YSZC12003_37197R1__bin.69	yeaW
SGB	Proteobacteria	Gammaproteobacteria	Enterobacterales	Morganellaceae	Providencia	Providencia_rettgeri	ZeeviD_2015__PNP_DietIntervention_31__bin.3	yeaX
SGB	Proteobacteria	Gammaproteobacteria	Enterobacterales	Morganellaceae	Providencia	Providencia_rettgeri	LiJ_2014__V1.UC39-4__bin.41	yeaX
SGB	Proteobacteria	Gammaproteobacteria	Enterobacterales	Enterobacteriaceae	Escherichia	Escherichia_albertii	BackhedF_2015__SID105_12M__bin.10	yeaX
SGB	Proteobacteria	Gammaproteobacteria	Enterobacterales	Enterobacteriaceae	Escherichia	Escherichia_coli	CM_madagascar__A82_02_1FE__bin.3	yeaX
SGB	Proteobacteria	Gammaproteobacteria	Enterobacterales	Enterobacteriaceae	Citrobacter	Citrobacter_werkmanii	VatanenT_2016__G80513__bin.7	yeaX
SGB	Proteobacteria	Gammaproteobacteria	Enterobacterales	Enterobacteriaceae	Citrobacter	Citrobacter_freundii	KarlssonFH_2013__S389__bin.5	yeaX
SGB	Proteobacteria	Gammaproteobacteria	Enterobacterales	Enterobacteriaceae	Citrobacter	Citrobacter_braakii	GeversD_2014__SKBSTL011__bin.2	yeaX
SGB	Proteobacteria	Gammaproteobacteria	Enterobacterales	Enterobacteriaceae	Klebsiella	Klebsiella_quasipneumoniae	QinJ_2012__DLM028__bin.4	yeaX
SGB	Proteobacteria	Gammaproteobacteria	Enterobacterales	Enterobacteriaceae	Klebsiella	Klebsiella_pneumoniae	NielsenHB_2014__O2_UC38_2__bin.18	yeaX
SGB	Proteobacteria	Gammaproteobacteria	Enterobacterales	Enterobacteriaceae	Klebsiella	Klebsiella_pneumoniae	VatanenT_2016__G80588__bin.5	yeaX
SGB	Proteobacteria	Gammaproteobacteria	Enterobacterales	Enterobacteriaceae	Klebsiella	Klebsiella_aerogenes	VatanenT_2016__G80555__bin.3	yeaX
SGB	Proteobacteria	Gammaproteobacteria	Enterobacterales	Enterobacteriaceae	Klebsiella	Klebsiella_aerogenes	VogtmannE_2016__MMRS86168210ST-27-0-0__bin.16	yeaX
SGB	Proteobacteria	Gammaproteobacteria	Enterobacterales	Yersiniaceae	Serratia	Serratia_liquefaciens	VatanenT_2016__G80472__bin.3	yeaX
SGB	Proteobacteria	Gammaproteobacteria	Pseudomonadales	Moraxellaceae	Acinetobacter	Acinetobacter_baumannii	WenC_2017__RA130__bin.22	yeaX
SGB	Proteobacteria	Gammaproteobacteria	Pseudomonadales	Moraxellaceae	Acinetobacter	Acinetobacter_ursingii	BackhedF_2015__SID78_M__bin.21	yeaX
SGB	Proteobacteria	Gammaproteobacteria	Xanthomonadales	Xanthomonadaceae	Stenotrophomonas	Stenotrophomonas_rhizophila	OhJ_2014__MET0293__bin.25	yeaX
SGB	Proteobacteria	Gammaproteobacteria	Pseudomonadales	Pseudomonadaceae	Pseudomonas	Pseudomonas_sp_AU11447	OhJ_2014__MET0293__bin.22	yeaX
SGB	Proteobacteria	Gammaproteobacteria	Pseudomonadales	Pseudomonadaceae	Pseudomonas	Pseudomonas_sp_M1	XieH_2016__YSZC12003_37197R1__bin.69	yeaX
SGB	Proteobacteria	Gammaproteobacteria	Pseudomonadales	Pseudomonadaceae	Pseudomonas	Pseudomonas_oleovorans	OhJ_2014__MET0054__bin.5	yeaX

Supplementary Table 2. Taxaonmic information of TMA-lyase from Human Microbiome Project database,4,644 gut prokaryotes (MGYG, Almeida et al. 2020 - PMID 32690973) and recapitulated 4,930 species-level genome bins (SGBs, Pasolli et al. 2019 - PMID 30661755).

Phylum	Genus	Species	Database	*cntA*	*cntB*	*cutC*	*cutD*	*yeaW*	*yeaX*
	Atopobium	*Atopobium minutum*	UHGG/SGB			+	+		
		Collinsella bouchesdurhonensis	UHGG			+	+		
	Collinsella	*Collinsella tanakaei*	UHGG			+	+		
Actinobacteria		*Collinsella_aerofaciens*	SGB			+	+		
	Eggerthella	*Eggerthella lenta*	HMP				+		
	Olsenella	*Olsenella uli*	HMP/SGB			+	+		
		Olsenella_umbonata	SGB			+	+		
		Bacteroides coprophilus	HMP				+		
		Bacteroides ovatus	HMP				+		
		Bacteroides sp. 1_1_14	HMP				+		
		Bacteroides sp. 1_1_6	HMP				+		
	Bacteroides	*Bacteroides sp. 3_1_23*	HMP				+		
Bacteroidetes		*Bacteroides sp. D2*	HMP				+		
		Bacteroides stercoris	HMP				+		
		Bacteroides thetaiotaomicron	HMP				+		
		Bacteroides_vulgatus	SGB			+	+		
		Bacteroides_xylanisolvens	SGB				+		
	Prevotella	*Prevotella ruminicola*	HMP				+		
Desulfobacterota_A	*Desulfovibrio*	*Desulfovibrio desulfuricans*	UHGG			+	+		
Dictyoglomi	*Dictyoglomus*	*Dictyoglomus thermophilum*	HMP				+		
Firmicutes	*Alkaliphilus*	*Alkaliphilus metalliredigens*	HMP			+	+		
		Alkaliphilus oremlandii	HMP			+	+		
		Anaerococcus hydrogenalis	HMP/UHGG/SGB			+	+		
		Anaerococcus obesiensis	UHGG			+	+		
	Anaerococcus	*Anaerococcus tetradius*	HMP/SGB			+	+		
		Anaerococcus vaginalis	HMP/UHGG			+	+		
		Anaerococcus_lactolyticus	SGB			+	+		
		Anaerococcus_sp_HMSC065G05	SGB				+		
	Anaeroglobus	*Anaeroglobus massiliensis*	UHGG			+	+		
		Anaeromassilibacillus sp001305115	UHGG				+		
	Anaeromassilibacillus	*Anaeromassilibacillus sp002159845*	UHGG				+		
		Anaeromassilibacillus_sp_An250	SGB				+		
	Anaerosalibacter_A	*Anaerosalibacter_A massiliensis*	UHGG			+	+		
	Blautia	*Blautia_obeum*	SGB			+	+		
		Ruminococcus_sp	SGB				+		
	Caecibacter	*Caecibacter massiliensis*	UHGG			+	+		
	Candidatus Desulforudis	*Candidatus Desulforudis audaxviator*	HMP				+		
	Christensenella_A	*Christensenella_A timonensis*	UHGG				+		
		Clostridium baratii	UHGG			+	+		
		Clostridium botulinum	HMP/UHGG			+	+		
		Clostridium carboxidivorans	HMP			+	+		
		Clostridium citroniae	UHGG			+	+		
		Clostridium clostridioforme	UHGG			+	+		
		Clostridium ihumii	UHGG			+	+		
		Clostridium ljungdahlii	HMP			+	+		
	Clostridium	*Clostridium paraputrificum*	UHGG/SGB			+	+		
		Clostridium senegalense	UHGG			+	+		
		Clostridium septicum	UHGG			+	+		
		Clostridium sporogenes	HMP/SGB			+	+		
		Clostridium tepidum	UHGG			+	+		
		Clostridium tetani	HMP/UHGG			+	+		
		Clostridium_disporicum	SGB			+	+		
		Clostridium_sp_ATCC_29733	SGB				+		
		Clostridium_sulfidigenes	SGB			+	+		
	Desulfitobacterium	*Desulfitobacterium hafniense*	HMP/UHGG			+	+		
	Desulfotomaculum	*Desulfotomaculum reducens*	HMP			+	+		
		Enterococcus asini	UHGG			+	+		
		Enterococcus avium	UHGG			+	+		
		Enterococcus caccae	UHGG			+	+		
	Enterococcus	*Enterococcus dispar*	UHGG/SGB			+	+		
Firmicutes		*Enterococcus raffinosus*	UHGG/SGB			+	+		
		Enterococcus_faecium	SGB			+	+		
		Enterococcus_gallinarum	SGB				+		
		Enterococcus_sp_HMSC05C03	SGB			+	+		
	Faecalibacterium	*Faecalibacterium_prausnitzii*	SGB				+		
	Flavonifractor	*Flavonifractor_sp_An10*	SGB			+	+		
	Fournierella	*Fournierella massiliensis*	UHGG			+	+		
	Gemmiger	*Gemmiger_sp_An194*	SGB			+	+		
	Halothermothrix	*Halothermothrix orenii*	HMP				+		
	Hungatella	*Hungatella effluvii*	UHGG			+	+		
		Hungatella hathewayi	HMP/UHGG/SGB			+	+		
	Johnsonella	*Johnsonella_ignava*	SGB			+	+		
		[Lachnoclostridium asparagiforme]	HMP/UHGG/SGB			+	+		
	Lachnoclostridium	*Lachnoclostridium saccharolyticum*	HMP/SGB			+	+		
		Clostridium lavalense	UHGG/SGB			+	+		
		Lachnoclostridium phytofermentans	HMP			+	+		
	Lactobacillus	*Lactobacillus_casei*	SGB			+	+		
		Megasphaera elsdenii/SGB	UHGG				+		
	Megasphaera	*Megasphaera_micronuciformis*	SGB			+	+		
		Megasphaera_sp_DJF_B143	SGB			+	+		
		Megasphaera_sp_NM10	SGB				+		
	Mogibacterium	*Mogibacterium_sp_CM50*	SGB			+	+		
	Paenibacillus_B	*Paenibacillus_B thiaminolyticus*	UHGG			+	+		
	Peptoanaerobacter	*Eubacterium_yurii*	SGB			+	+		
		Peptoanaerobacter_stomatis	SGB				+		

	Peptoclostridium	*[Clostridium] hiranonis*	HMP				+		
	Peptococcus	*Peptococcus niger*	UHGG/SGB			+	+		
	Peptoniphilus_E	*Peptoniphilus_E obesi*	UHGG				+		
	Pseudoflavonifractor	*Pseudoflavonifractor capillosus*	HMP				+		
	Romboutsia	*Romboutsia dakarense*	UHGG			+	+		
	Roseburia	*Roseburia_faecis*	SGB				+		
	Ruminiclostridium	*[Clostridium] papyrosolvens*	HMP				+		
		Clostridium_leptum	SGB			+	+		
		Eubacterium_siraeum	SGB				+		
		Ruminiclostridium thermocellum	HMP				+		
	Symbiobacterium	*Symbiobacterium thermophilum*	HMP				+		
	Thermincola	*Thermincola potens*	HMP				+		
	Thermosinus	*Thermosinus carboxydivorans*	HMP				+		
	unclassified Eubacteriales	*Clostridiales bacterium 1_7_47FAA*	HMP			+	+		
	Vagococcus	*Vagococcus_teuberi*	SGB			+	+		
	Veillonella	*Veillonella_seminalis*	SGB			+	+		
Fusobacteria	*Cetobacterium*	*Cetobacterium_somerae*	SGB			+	+		
	Fusobacterium	*Fusobacterium_varium*	SGB			+			
Proteobacteria	*Achromobacter*	*Achromobacter piechaudii*	HMP	+	+			+	+
	Acinetobacter	*Acinetobacter baumannii*	HMP/UHGG/SGB	+	+			+	+
		Acinetobacter nosocomialis	HMP/UHGG	+	+			+	+
		Acinetobacter oleivorans	HMP	+	+			+	+
		Acinetobacter pittii	HMP/UHGG	+	+			+	+
		Acinetobacter_ursingii	SGB	+	+			+	+
	Aeromonas	*Aeromonas hydrophila*	HMP			+	+		
	Arcobacter	*Arcobacter nitrofigilis*	HMP				+		
	Bilophila	*Bilophila_sp_4_1_30*	SGB			+	+		
		Bilophila_wadsworthia	SGB			+	+		
	Burkholderia	*Burkholderia multivorans*	HMP	+	+			+	+
	Citrobacter	*Citrobacter braakii*	UHGG/SGB	+	+			+	+
		Citrobacter europaeus	UHGG	+	+			+	+
		Citrobacter freundii	UHGG/SGB			+	+		
		Citrobacter pasteurii	UHGG			+	+		
		Citrobacter portucalensis	UHGG	+	+			+	+
		Citrobacter sp. 30_2	HMP	+	+			+	+
		Citrobacter youngae	HMP	+	+			+	+
		Citrobacter_werkmanii	SGB	+	+			+	+
	Cupriavidus	*Cupriavidus necator*	HMP	+	+			+	+
		Cupriavidus pinatubonensis	HMP	+	+			+	+
	Dechloromonas	*Dechloromonas aromatica*	HMP				+		
	Desulfatibacillum	*Desulfatibacillum alkenivorans*	HMP				+		
	Desulfobacterium	*Desulfobacterium autotrophicum*	HMP				+		
	Desulfotalea	*Desulfotalea psychrophila*	HMP			+	+		
	Desulfovibrio	*Desulfovibrio aespoeensis*	HMP				+		
		Desulfovibrio alaskensis	HMP			+	+		
		Desulfovibrio desulfuricans	HMP/SGB			+	+		
		Desulfovibrio magneticus	HMP				+		
		Desulfovibrio salexigens	HMP			+	+		
		Desulfovibrio sp. FW1012B	HMP				+		
		Desulfovibrio_legallii	SGB			+	+		
		Desulfovibrio_piger	SGB			+	+		
	Dickeya	*Dickeya chrysanthemi*	HMP				+		
	Enterobacter	*Enterobacter cancerogenus*	HMP/UHGG			+			
	Escherichia	*Escherichia albertii*	HMP/UHGG/SGB	+	+			+	+
		Escherichia coli	HMP/UHGG/SGB	+	+	+	+	+	+
		Escherichia fergusonii	HMP/UHGG			+	+		
		Escherichia sp. 4_1_40B	HMP	+	+			+	+
	Franconibacter	*Franconibacter helveticus*	UHGG			+			
	Geobacter	*Geobacter bemidjiensis*	HMP				+		
		Geobacter daltonii	HMP				+		
		Geobacter metallireducens	HMP				+		
		Geobacter sp. M21	HMP				+		
		Geobacter sulfurreducens	HMP				+		
		Geobacter uraniireducens	HMP				+		
	Hyphomicrobium	*Hyphomicrobium denitrificans*	HMP		+				
	Klebsiella	*Klebsiella aerogenes*	UHGG/SGB	+	+	+	+	+	+
		Klebsiella grimontii	UHGG			+	+		
		Klebsiella michiganensis	UHGG/SGB			+	+		
		Klebsiella oxytoca	UHGG/SGB			+	+		
		Klebsiella pneumoniae	HMP/UHGG/SGB	+	+	+	+	+	+
		Klebsiella quasipneumoniae	UHGG/SGB	+	+	+	+	+	+
		Klebsiella sp. 1_1_55	HMP	+	+			+	+
		Klebsiella variicola	HMP/UHGG	+	+	+	+	+	+
		Klebsiella_sp_Kd70_TUC_EEAOC	SGB			+	+		
	Kluyvera	*Kluyvera ascorbata*	UHGG/SGB			+	+		
	Leclercia	*Leclercia adecarboxylata*	UHGG			+	+		
	Methylophaga	*Methylophaga thiooxydans*	HMP		+				+
	Methylotenera	*Methylotenera versatilis*	HMP		+				+
	Methylovorus	*Methylovorus glucosotrophus*	HMP				+		
	Moellerella	*Moellerella wisconsensis*	UHGG			+	+		
	Pectobacterium	*Pectobacterium atrosepticum*	HMP			+	+		
		Pectobacterium carotovorum	HMP			+	+		
		Pectobacterium wasabiae	HMP			+	+		
	Pelobacter	*Pelobacter carbinolicus*	HMP			+	+		
		Pelobacter propionicus	HMP				+		
	Proteus	*Proteus mirabilis*	HMP/UHGG/SGB			+	+		
		Proteus penneri	HMP/UHGG			+	+		
		Proteus terrae	UHGG			+	+		

		Proteus vulgaris	UHGG/SGB			+	+		
	Providencia	*Providencia alcalifaciens*	HMP/UHGG	+	+	+	+	+	+
		Providencia rettgeri	HMP/UHGG/SGB	+	+	+	+	+	+
		Providencia rustigianii	HMP/UHGG			+	+		
		Providencia stuartii	HMP/UHGG	+	+			+	+
	Pseudomonas	*Pseudomonas citronellolis*	UHGG	+	+			+	+
		Pseudomonas entomophila	HMP	+	+			+	+
		Pseudomonas putida	HMP	+	+			+	+
		Pseudomonas_oleovorans	SGB						+
		Pseudomonas_sp_AU11447	SGB	+	+			+	+
		Pseudomonas_sp_M1	SGB	+	+			+	+
		Pseudomonas_sp_T	SGB		+				
	Raoultella	*Raoultella ornithinolytica*	UHGG/SGB			+	+		
	Rhizobium	*Rhizobium etli*	HMP				+		
	Ruegeria	*Ruegeria pomeroyi*	HMP		+				
	Serratia	*Serratia liquefaciens*	UHGG/SGB	+	+			+	+
		Serratia marcescens	UHGG	+	+			+	+
		Serratia ureilytica	UHGG	+	+			+	+
	Shewanella	*Shewanella benthica*	HMP				+		
		Shewanella oneidensis	HMP				+		
	Shigella	*Shigella boydii*	HMP	+	+			+	+
		Shigella flexneri	HMP	+	+			+	+
		Shigella sonnei	HMP	+	+			+	+
	Sorangium	*Sorangium cellulosum*	HMP				+		
	Stenotrophomonas	*Stenotrophomonas_rhizophila*	SGB	+	+			+	+
	Syntrophobacter	*Syntrophobacter fumaroxidans*	HMP				+		
	Syntrophus	*Syntrophus aciditrophicus*	HMP				+		
	Thiobacillus	*Thiobacillus denitrificans*	HMP				+		
	unclassified Rhodobacteraceae	*Rhodobacteraceae bacterium KLH11*	HMP		+				
	Vibrio	*Vibrio alginolyticus*	HMP				+		
	Vitreoscilla	*Vitreoscilla massiliensis*	UHGG	+	+			+	+
	Xenorhabdus	*Xenorhabdus nematophila*	HMP	+	+			+	+
	Yersinia	*Yersinia pestis*	HMP/UHGG	+	+			+	+
		Yersinia pseudotuberculosis	HMP	+	+			+	+
	Yokenella	*Yokenella regensburgei*	UHGG			+	+		
Spirochaetes	*Brachyspira*	*Brachyspira pilosicoli*	HMP				+		
Thermotogae	*Marinitoga*	*Marinitoga piezophila*	HMP				+		
	Thermotoga	*Thermotoga maritima*	HMP				+		
		Thermotoga naphthophila	HMP				+		
		Thermotoga petrophila	HMP				+		
		Thermotoga sp. RQ2	HMP				+		
Verrucomicrobia	*Akkermansia*	*Akkermansia_glycaniphila*	SGB				+		
	Verrucomicrobium	*Verrucomicrobium_sp_GAS474*	SGB				+		

Supplementary Table 3. Detailed information of public datasets used in this study.

Abbreviation	Country	Total number of individuals	The number of healthy individuals	Gender (F/M)	Age (mean±SD)	BMI (mean±SD)	Individuals with colorectal cancer (CRC)	Individuals with adenoma	Individuals with type 2 diabetes	Individuals with impaired glucose tolerance	Individuals with cardiovascular disease	Individuals with hypertension	Individuals with Obesity	Relative abundance of taxonomic classification					
														phylum	class	order	family	genus	species
HZ	HADZA	26	26	9/18	32.00±14.93	-	-	-	-	-	-	-	-	0.7877±0.06737	0.7059±0.08115	0.6969±0.08258	0.4731±0.09551	0.4142±0.09964	0.2638±0.0754
CN	China	226	127	26/45	33.69±11.02	22.33±3.52	-	-	99	-	-	-	-	0.9155±0.03474	0.8795±0.05102	0.8762±0.05309	0.7269±0.08102	0.6614±0.09430	0.3540±0.08342
JP	Japan	106	98	42/62	31.96±10.78	21.67±2.260	-	-	-	-	-	-	-	0.9329±0.02294	0.8920±0.03415	0.8875±0.03531	0.7042±0.05880	0.6172±0.06044	0.3804±0.06251
KR	South Korea	35	35	-	-	-	-	-	-	-	-	-	-	0.8630±0.06961	0.8078±0.09593	0.8022±0.10110	0.6292±0.1330	0.5720±0.1363	0.3717±0.06883
DK	Denmark	85	47	24/24	54.69±10.70	23.24±2.35	-	-	35	-	-	-	-	0.8579±0.05343	0.7964±0.07331	0.7875±0.07730	0.6065±0.08619	0.5614±0.09394	0.3300±0.05661
SE	Sweden	145	35	36/0	70.36±0.73	24.32±2.93	-	-	53	49	-	-	-	0.8748±0.03471	0.8072±0.05319	0.7994±0.05695	0.6000±0.07385	0.5350±0.08098	0.3982±0.0653
AT	Austria	156	41	19/22	66.37±7.21	25.63±3.21	46	44	-	-	-	-	-	0.8681±0.08061	0.8089±0.08903	0.8025±0.09075	0.6309±0.1050	0.5315±0.09511	0.4079±0.07749
FR	France	156	51	30/25	60.62±11.88	23.97±2.41	53	42	-	-	-	-	-	0.8656±0.04605	0.8088±0.06319	0.8022±0.06595	0.6360±0.07894	0.5702±0.08355	0.3792±0.06039
AU	Austrilia	25	25	-	-	-	-	-	-	-	-	-	-	0.8520±0.04775	0.8066±0.06033	0.7966±0.06714	0.6492±0.08069	0.6145±0.08653	0.3343±0.05526
US	America	300	148	66/82	-	-	-	-	-	-	-	-	-	0.9178±0.04441	0.88760±0.064	0.8843±0.06679	0.7525±0.1006	0.7237±0.1196	0.3058±0.0755
PE	Peru	36	12	12/19	20.43±15.55	20.55±4.15	-	-	-	-	-	-	-	0.7553±0.06890	0.6674±0.09728	0.6514±0.09986	0.4607±0.1286	0.4051±0.1214	0.2864±0.07902
ACVD*	China	385	171	154/226	60.74±9.87	24.52±5.48	-	-	-	-	214	-	-	0.9144±0.03432	0.8706±0.05301	0.8667±0.05539	0.7131±0.08385	0.6304±0.09955	0.3709±0.07515
HT	China	196	99	-	-	-	-	-	-	-	-	97	-	0.9101±0.04198	0.8754±0.06105	0.8704±0.06388	0.7265±0.09289	0.6770±0.1060	0.4171±0.1290
OB	China	257	105	-	-	-	-	-	-	-	-	-	152	0.9198±0.03181	0.8838±0.04757	0.8810±0.04930	0.7298±0.07745	0.6784±0.09362	0.3827±0.08503
Total		2134	1020				99	86	187	49	214	97	152						

Supplementary Table 4. Homologous modeling and molecular docking between cutC sequences of trains from cutC abundant strains and coline.

Genus	Source of strain	Protein sequence
Lachnoclostridium	*Lachnoclostridium saccharolyticum WM1*	LKEREIRLDIREFSNKFVEATKNMTPEERASLMKMFETVSDEINKKESSSQAAACCEGGT<br>EVPEGITPRLQKLKDNYLTHKPSITTYRARAITKIAKENPGMPKIMLRAKCFRYCCETAP<br>LVIQDNELIVGAPCGAPRAGAFSPDIAWRWMVDEIDTIGTRPQDPFYISEEDKKIMKEEL<br>FPYWAGKSVDEYCEDQYREAGVWELSGESFVSDCSYHAINGGGDSNPGYDVILMKKGMLD<br>IQQEAKDHLKELDYENPDDIEKIYFYKSIIDTTEGVMIYAKRLSEYAAELAAKETNPKRK<br>EELLKISEVNAYVPAHKPRTFWEAIQAVWTIESLLVVEENQTGMSIGRVDQYMYPFYKDD<br>IESGRMNDYQAFELAGCMLIKMSEMMWITSEGGSKFFAGYQPFVNMCVGGVTRSGHDATN<br>DLTYLLMDAVRHVKIYQPSLACRIHNKSPKKYMKKIVDVVRSGMGFPACHFDDAHIKMML<br>AKGVSIEDARDYCLMGCVEPQKSGRLYQWTSTAYTQWPICIELVLNHGAPLWYGKQVCPD<br>MGDLSNFKTYEEFEAAVKEEIKYITKWTDVATVISQRVHRDLAPKPLMSIMYEGCMEKAK<br>DVSAGGAMYNFGPGVVWSGLATYADSMAAIKKLVFEEKKYTLEQLNQALKADFVGYDQIR<br>TDCLNAPKYGNDDDYADLIAADLVDFTEHEHRKYKTLYSVLSHGTLSISNNTPFGQMTGA<br>SANGRNAWLPLSDGISPTQGADFKGPTAIIKSVSKMSNDSMNIGMVHNFKIMAGLLDTPE<br>GEESLITLLRTACMFGNGEMQFNYLDNDTLVEAQKHPELYRDLIVRVAGYSAFFVELCKD<br>VQDEIISRTMLTHF
	[Lachnoclostridium asparagiforme] DSM 15981	DEIDTIGSRPQDPFYISEEDKKIMREELFPFWKGKSVDEYCEDQYREAGVWELSGESFVS<br>DCSYHALNGGGDSNPGYDVILMKKGMLDIQQEAREHLEQLDYENPDDIEKIYFYKSIIDT<br>TEGVMIYARRMSEYAAQLAAKEQNPRRREELLKISEVNARVPAHKPETFWEAIQAVWTIE<br>SLLVVEENQTGMSIGRVDQYMYPFYKGDIASGRLNDRQAFELAGCMLIKMSEMMWITSEG<br>GSKFFAGYQPFVNMCVGGVTRDGRDATNELTYLLMDAVRHVRIYQPSLACRIHNKSPKKY<br>LKKIVDVVRSGMGFPACHFDDAHIKMMLAKGVSIEDARDYCLMGCVEPQKSGRLYQWTST<br>SYTQWPICIELALNHGVPLWYGKQVCPDMGDPDRFKTYEEFEAAVKDEIKFITKWTAVAT<br>VISQRVHKELAPKPLMSIMYEGCMEKGRDVSAGGAMYNFGPGVIWSGLATYADSMAAIRK<br>LVFDDRKYTLSQINEALKADFAGFDQLRIDCLNAPKYGNDDDYADLIAADLVNFTEHEHR<br>KYKTLYSTLSHGTLSISNNTPFGQMTGASANGRHAWLPLSDGISPTQGADFKGPTAIIKS<br>VSKMSNDSMNIGMVHNFKIMAGLLDTPEGEESLITLLRTACMFGNGEMQFNYLDNNTLIE<br>AQKHPEQYRDLIVRVAGYSAFFVELCKDVQDEIISRTMLTHF
	Lachnoclostridium phytofermentans ISDg	LDIREFSNKFVEATKNMSPEERSSLMKMFETVSDEINKKETAPSTSNVCNEEGSNIPDGI<br>TPRLHKLKENYLTHKPTITTYRARAITKIAKENPGMPKIMLRAKCFRYCCETAPLVIQDN<br>ELIVGAPCGAPRAGAFSPDIAWRWMVDEIDTIGTRPQDPFYISEEDKKIMKEELFPFWAG<br>KSVDEYCEDQYREAGVWELSGESFVSDCSYHAINGGGDSNPGYDVILMKKGMLDIQQEAK<br>DHLKELDYENPDDIEKIYFYKSIIDTTEGVMIYAKRLSEYAAELAAKETNPKRKEELLKI<br>SEVNAYVPAHKPRTFWEAIQAVWTIESLLVVEENQTGMSIGRVDQYMYPFYKDDIESGRM<br>NDYQAFELAGCMLIKMSEMMWITSEGGSKFFAGYQPFVNMCVGGVTRSGRDATNDLTYLL<br>MDAVRHVKIYQPSLACRIHNKSPKEYLKKIVDVVRSGMGFPACHFDDAHIKMMLAKGVSI<br>EDARDYCLMGCVEPQKSGRLYQWTSTAYTQWPICIELVLNNGVPLWYGKQVCPDMGALSN<br>FKTYEEFEAAVKEQIKYITKWSDVATVISQRIHRDLAPKPLMSIMYEGCMESAKDVSAGG<br>AMYNFGPGVVWSGLATYSDSMAAIKKLVFDEKKYTLEQLNEALKADFAGYDQIRTDCLNA<br>PKYGNDDDYADLIAADLVDFTEHEHRKYKTLYSILCHGTLSISNNTPFGQLTGASANGRH<br>AWLPLSDGISPTQGADFNGPTAIIKSISKMANDSMNLGMVHNFKIMSGLLDTPEGEESLI<br>TLLRTACMFGNGEMQFNYLDNNTLIDAQKHPEKYRDLIVRVAGYSAFFIELCKDVQDEII<br>SRTMLTHF
	Clostridium sporogenes ATCC 15579	MEATKNMSDEERAGLMKMFQSVSNEITKEEPATSKVACDNNGEIPDGMTERLVKLKENYM<br>KHVPSITTHRARAITKIAKENPGIPKSVLRGKCFKHCCETAPLLIQDHELIVGAPNGKPR<br>AGAFSPDIAWRWMVDEIDTIGTRPQDPFYISEEDKKIMREELFPYWAGKSVDEYCEDQYR<br>EAGVWELSGESFVSDCSYHAVNGGGDSNPGYDVVLMKKGMLDIKREAEEKLAELKYENPE<br>DIDKIYFYKSLIDTAEGVMIYAKRMSDYAAELAQKETNPKRKAELQKISEINARVPAHKP<br>STYWEAIQAVWTIESLLVVEENQTGMSIGRVDQYMYPFYKADIEAGRMTDYEAFELSGCM<br>LIKMSEMMWITSEGGSKFFAGYQPFVNMCLGGVTREGRDATNELTYLLMDAVRHVKIYQP<br>SLACRIHKGSPQKYLKKIVDVIRAGMGFPACHFDDVHIKIMLAKGVSIEDARDYCLMGCV<br>EPQKSGRLYQWTSTGYTQWPICIELVLNHGVPLWYGKQVCPDMGDLSQFKTYEQFEAAVK<br>EQIKFITKWTSVATVISQRVHKELAPKPLMSMMYEGCMENGKGVEAGGAMYNFGPGVVWS<br>GLATYTDSMAAIKKLVFEDKKYTLQEMNEALKADFVGYEQLKKDCLEAPKYGNDDDYADL<br>IAADLINFTEQEHRKYKTLYSVLSHGTLSISNNTPFGQMTGATANGRRAWMPLSDGISPS<br>QGADFKGPTSIIKSVSKMSCEDMNIGMVHNFKLISGLLDTPEGEQGIITLLRSACALQLG<br>EVQFNYLDNKTLIEAQKHPDQYRDLIVRVAGYSAFFVELCKDVQDEIISRTMLTHF

Clostridium	*Clostridium botulinum E1 str. BoNT E Beluga*	MRGKKLDIREFSNKFVEATQNMSAEERECLMKIFEGVSKEITKEEPVLEHVVCTEGDKIP<br>DGITQRLLKLKENYLKQVPSITTYRARAITKIAKENPGMPKILLRAKCFKYCCETAPLVI<br>QDNELIVGAPCGAPRAGAFSPDIAWRWMVDEIDTIGTRAQDPFYISEEDKKIMREELFPY<br>WAGKSVDEYCENQYREAGVWELSGESFVSDCSYHAINGGGDSNPGYDVILMKKGMLDIQL<br>EAKEHLEKLDYENPEHIEKIYFYKAIIDTTEGVMIYAKRLSKYAAELAEKETNPKRKSEL<br>QKISEINARVPAHKPSTFWEAIQAVWTIESLLVVEENQTGMSIGRVDQYMYEYYKNDIES<br>GRMNDFEAFELSGCMLIKMSEMMWITSEGGSKFFAGYQPFVNMCVGGVDRQGRDATNELT<br>YLLMDAVRHVKIYQPSLACRIHNQSPQKYLKKIVDVIRAGMGFPACHFDDVHIKMMLAKG<br>VSIEDARDYCLMGCVEPQKSGRLYQWTSTAYTQWPICIELVLNNGIPLWYEKQVCPNMGD<br>INSFKNYEEFESAVKEQIKYITKWTSVATVISQRVHKELAPKPLMSIMYEGCMDNGRGVE<br>AGGAMYNFGPGVIWSGLATYTDSMAAIKKLVFEEKKYTLQELNEALKADFVGYEKLRKDC<br>LEAPKYGNDDDYADYIAADLINFTELEHRKFKTLYSVLSHGTLSISNNTPFGQLTGATAN<br>GRRAWMPLSDGISPSQGSDYKGPTAIIKSVSKMSCDSMNIGMVHNFKLISGLLDTKEGED<br>GIITLLRSACVLGLGEVQFNYLDNDTLIEAQKHPEQYRDLIVRVAGYSAFFVELCKDVQD<br>EIISRTMLTHF
	Clostridium botulinum A2 str. Kyoto	MEATKNMSDEERAGLMKMFQSVSNEITREEPATSKVACDNNGEIPDGMTERLVKLKETYL<br>KHVPTITTHRARAITKIAKENPGTPKSVLRGKCFKHCCETAPLVIQDNELIVGAPNGQPR<br>AGAFSPDIAWRWMVDEIDTIGTRPQDPFYISEEDKKIMREELFPYWAGKSVDEYCEDQYR<br>EAGVWELSGESFVSDCSYHAVNGGGDSNPGYDVVLMKKGMLDIKREAEEKLAELKYENPE<br>DIDKIYFYKSLIDTAEGVMIYAKRMSDYAAELAQKETNPKRKAELLKISEINARVPAHKP<br>STYWEAIQAVWTIESLLVVEENQTGMSIGRVDQYMYPFYKADIEAGRMTDYEAFELSGCM<br>LIKMSEMMWITSEGGSKFFAGYQPFVNMCVGGVTREGRDATNELTYLLMDAVRHVKIYQP<br>SLACRIHKSSPQKYLKKIVDVIRAGMGFPACHFDDVHIKIMLAKGVSIEDARDYCLMGCV<br>EPQKSGRLYQWTSTGYTQWPICIELVLNNGVPLWYGKQVCPDMGDLSQFKTYEQFEAAVK<br>EQIKFITKWTSVATVISQRVHKELAPKPLMSMMYEGCMENGRGVEAGGAMYNFGPGVVWS<br>GLATYADSMAAIKKLVFEDKKYTLQEMNEALKADFVGYEQLRKDCLEAPKYGNDDDYADL<br>IAADLINFTEQEHRKYKTLYSVLSHGTLSISNNTPFGQMTGATANGRRAWMPLSDGISPS<br>QGADFKGPTSIIKSVSKMSCEDMNIGMVHNFKLIAGLLDTPEGEQGIITLLRSACALQLG<br>EVQFNYLDNKTLIEAQKHPDQYRDLIVRVAGYSAFFVELCKDVQDEIISRTMLTHF
	Clostridium botulinum Bf	MEATKNMSDEERAGLMKMFQSVSNEITKEETTAPKVACDNNGEIPDGMTERLVKLKENYM<br>KHVPTITTHRARAITKIAKENPGTPKSVLRGKCFKHCCETAPLVIQDNELIVGAPNGQPR<br>AGAFSPDIAWRWMVDEIDTIGTRPQDPFYISEEDKKIMREELFPYWAGKSVDEYCEDQYR<br>EAGVWELSGESFVSDCSYHAVNGGGDSNPGYDVVLMKKGMLDIKREAEEKLAELKYENPE<br>DIDKIYFYKSLIDTAEGVMIYAKRMSDYAAELAQKETNPKRKAELQKISEINARVPAHKP<br>STYWEAIQAVWTIESLLVVEENQTGMSIGRVDQYMYPFYKADIEAGRMTDYEAFELSGCM<br>LIKMSEMMWITSEGGSKFFAGYQPFVNMCVGGVTREGRDATNELTYLLMDAVRHVKIYQP<br>SLACRIHKGSPQKYLKKIVDVIRAGMGFPACHFDDVHIKMMLAKGVSIEDARDYCLMGCV<br>EPQKSGRLYQWTSTGYTQWPICIELVLNHGVPLWYGKQVCPDMGDLSQFKTYEQFEAAVK<br>EQIKFITKWTSVATVISQRVHKELAPKPLMSMMYEGCMENGRGVEAGGAMYNFGPGVVWS<br>GLATYADSMAAIKKLVFEDKKYTLQEMNEALKADFVGYEQLRKDCLEAPKYGNDDDYADY<br>IASDLINFTEQEHRKYKTLYSVLSHGTLSISNNTPFGQMTGATANGRRAWMPLSDGISPS<br>QGADFKGPTSIIKSVSKMTCEDMNIGMVHNFKLISGLLDTPEGEQGIITLLRSACALQLG<br>EVQFNYLDNKTLIEAQKHPEQYRDLIVRVAGYSAFFVELCKDVQDEIISRTMLTHF
	Clostridium carboxidivorans P7	MKYLKECEVINLDIREFSSKFMEATKNMTEEERASLMKMFSSVSKEITKEEPAHICVTGD<br>NNGQIPDGITERLVKLKETYLKHVPSITTHRARAITKIAKENPGIPKSVLRGKCFKHCCE<br>TAPLVIQDNELIVGAPNGQPRAGAFSPDIAWRWMVDEIDTIGTRPQDPFYISEEDKKIMR<br>EELFPYWQGKSVDEYCEDQYREAGVWELSGESFVSDCSYHAVNGGGDSNPGYDVILMKKG<br>MLDIKREAEDKLAELNYENPEDIDKIYFYKSLIDTAEGVMIYAKRMSDYAAELAAKETNP<br>KRKAELQKISEVNARVPAHKPSTYWEAIQSVWTIESLLVVEENQTGMSIGRVDQYMYPFY<br>KADIEAGRMTDFEAFELSGCMLIKMSEMMWITSEGGSKFFAGYQPFVNMCLGGVTREGRD<br>ATNDLTYLLMDAVRHVKIYQPSLACRIHKASPQKYLKKIVDVIRAGMGFPACHFDDVHIK<br>IMLAKGVSIEDARDYCLMGCVEPQKAGRLYQWTSTGYTQWPICIELVLNHGVPLWYGKQV<br>CPNLGDLSNFKTYEQFEAAVKEQIKYITKWTSVATVISQRVHKDLAPKPLMSMMYEGCME<br>KGRGVEAGGAMYNFGPGVVWSGLATYTDSMAAIKKLVFDDKKYTLQQMNEALKADFAGYE<br>QLRKDCLEAPKYGNDDDYADLIAADLINFTEQEHRKYKTLYSVLSHGTLSISNNTPFGQL<br>TGATANGRRAWTPLSDGISPTQGADYKGPTSIIKSVSKMSCEDMNIGMVHNFKLIAGLLD<br>TPEGEQGIITLLRSACALQLGEMQFNYLDNKTLLDAQKHPEEYRDLIVRVAGYSAFFVEL<br>CKDVQDEIISRTVLTHF

	Clostridium ljungdahlii DSM 13528	LDIREFSNKFAEATKNMSAEEQAALMKIFQGVSQEITKEDNGAPAVSAEPCTNNGEVPNG<br>MTERLKRIKENYLKQKPSITTYRAKAITKIAKENPGMPKILLRAKCFRYCCETAPLVIQD<br>DELIVGAPCGAPRAGAFSPDIAWRWMQDEIDTIGTRPQDPFYVSEEDKKVMREELFPFWQ<br>GKSVDEYCEDQYREAGVWELSGESFVSDCSYHATNGGGDSNPGYDVILMKKGMADIQQEA<br>KDHLAKLDYENPEDIEKIYFYKSVIDTTEGVMIYAKRMSEYAAELAAKETNPKRKAELQK<br>ISEVNAHVPAHKPSNFWEAIQSVWTIESLLVVEENQTGMSIGRVDQYMYPFYKADIESGR<br>MNNYEAFELAGCMLIKMSEMMWVTSEGASKFFAGYQPFVNMCVGGVTRDGLDATNDLTYL<br>LMDAVRHVKIYQPSLACRIHNKSPKKYLKKIVDVVRAGMGFPACHFDDAHIKMMLAKGVS<br>IEDARDYCIMGCVEPQKAGRLYQWTSTAYTQWPICIELVLNHGVPLWYGKQVCPDMGDLN<br>RFKTYEEFDAAVKEEIKYITKWTDVATVISQRVHRDLAPKPLMSIMYEGCMEHGKDVSAG<br>GAMYNFGPGVVWTGLATYADSMAAIKKVVFDDKKCTLEQLNQALKADFVGYEELRNDCLK<br>APKYGNDDDYADLIAADLINFTEMEHRKYKTLYSVLSHGTLSISNNTPFGQLTGASANGR<br>KAWTPLSDGISPTQGADFKGPTAIIKSVSKMSCDNMNIGMVHNFKLMAGLLETPEGEEGI<br>ITLLRTACLFGNGQMQFNYLDNKTLIEAQKHPEQYRDLIVRVAGYSAFFVELCKDVQDEI<br>ISRTMLTHF
	Clostridium tetani E88	MEATKNMSDEERNGLMNMFQSISKEIKKEEKVTSNVVFNNNGEIPDGMTERLIKLKENYM<br>KQVPSITTHRARAITKIAKENPGVPKSVLRGKCFKYCCETAPLVIQDNELIVGAPNGKPR<br>AGAFSPDIAWRWMEDEIDTIANRPQDPFYISEEDKKIMREELFPYWKGKSVDEYCEDQYR<br>EAGVWELSGESFVSDCSYHAVNGGGDSNPGYDVILMKKGMLDIKREAEEKLASLSYERPE<br>DIEKIYFYKSIIDTAEGVMIYAKRMSDYAAELAAKETDPKRKAELQKISKVNARVPAHKP<br>STFWEAIQAVWTIESLLVVEENQTGMSIGRVDQYMYPFYKSDIESGRMTDFEAFELAGCM<br>LIKMSEMMWITSEGGSKFFAGYQPFVNMCVGGVTREGRDATNELTYLLMDAVRHVKIYQP<br>SLACRIHKGSPQKYLKKIVDVIRAGMGFPACHFDDVHIKMMLAKGVSIEDARDYCLMGCV<br>EPQKSGRLYQWTSTGYTQWPICIELVLNHGVPLWYGKQVCPDMGDLSQFKTYEQFEGAVR<br>EQIKYITKWTAVATTISQRVHRELAPKPLMSMMYEGCMEKGRGVEAGGAMYNFGPGVVWS<br>GLATYTDSMAAIKKLVFEEKKYTLEELSEALKADFVGYERLRKDCLEAPKYGNDDDYADY<br>IAADLVNFTEQEHRKYKTLYSVLSHGTLSISNNTPFGQMTGATANGRRAWMPLSDGISPS<br>QGSDFKGPTSIIKSVSKISCEDMNIGMVHNFKLMSGLLDTPEGEQGIIALLRSACALQLG<br>EIQFNYLDNETLIEAQKHPEQYRDLIVRVAGYSAFFVELCKDVQDEIISRTMLTHF
Klebsiella	*Klebsiella pneumoniae subsp. rhinoscleromatis ATCC 13884*	MAHYNLTPRVKVLADRLLAQKSTLCTEHATTLNALDGDIAGVPAAVKPARRFYELMRQLP<br>LTISADELIVGNQTRKPHGAIFHDENATRRPSVFQFLNLNSELDSPDYKLVVEKGVLAIK<br>HQLEEKTRALGSAVSRSGMDEVNGCRAAIYACDALLALAQNLANSAEQLAAAETNAYRKA<br>ELLDSAAILHHVPAHPARNFKEACQAFYLFQLALQLDNGSYAVNPQGADIALLPYFQRDI<br>NSGALNTQQAYEIVECLWFKLAELSEIRAACAIDGYPMLDAMLHGAAFDHAEVNELSAMF<br>ISAQRNLSALNLPVRMFSGVQPVSHAPFAACADTPAMEGLTPRMQRLRNHYLTVRPSVSI<br>YRALAFTEVVKANPGMPTILLRAKAFRHACETAPILIQDDELIVGHPCGKPRAGAFSPDI<br>AWRWVRDELDTMSTRPQDPFEISEADKKTIREEIVPFWEGRSLDEICEAQYREAGVWAFS<br>GETFVSDLSYHQINGGGDTCPGYDVLLFTKGMNGIKADAEAHLASLSMENPEDIDRIYYY<br>KAAIETCEGVVNYARRIAAHARELAAKEQNAQRRAELLTIAYVNENVPANPPKTLQEALQ<br>SIWTVESLFEIEENQTGLSLGRVDQYSYPMFEADIREGRLTHDSALELLQAFIIKCAELM<br>WMSSELGAKYFAGYQPFINLTVGGQKRSGGDACNDLTYLIMDAVRFVKVYQPSLACRIHN<br>QSPQKYMEKIVDVVKAGMGFPACHFDDSHIKMMLRKGFDFEDARDYCLMGCVEPQKSGRI<br>YQWTSTGYTQWPIAIEFVLNRGRMVLFDSYQGLDTGDLRDLRTFEDFDAAVKQQIAHIVR<br>LSAIGTVISQRVHRDVAPKPLMSLLVEGCMESGKDVAAGGAMVNHGPGLIFSGLATYVDS<br>MAAIRKLVFEEKKYTLEQIRDALLANFEGYEALRRDCLNAPKYGNDDNYVDQYALDITEW<br>TEKECRKYKMLYSTLSHGTLSISNNTPIGELTNATPNGRLAWMPLSDGISPTQGADKQGP<br>TAIIKSVSKMNVETMSIGMVHNFKFLKGLLDTPEGRHGLITLLRTASILGNGQMQFSYVD<br>NEVLKKAQQEPEKYRDLIVRVAGYSAYFVELCKEVQDEIISRTVIEKF
Escherichia	*Escherichia fergusonii ATCC 35469*	MANYNLTPRVKVLAERLLAHPSTLCVEHAGILSGLDGDIAGIPAAVKPARRFYELMRQLP<br>LAVSPDELIVGNQTHRPHGAIFHDESTAHRPSVFQFLNLNSDLDAPDYKLVIEKGVLAIK<br>QQLEEKTRSLGSAVSRSGMDEVNACRAAIYACDALMQLAQNLATSAEKLAATETNAYRKA<br>ELSESAAILHHIPAHPARSFKEACQAFYLFQLALQLDNGSYAVNPEGADKALLAYYQHDI<br>ANGLLTEAQAYEIVECLWFKLAELSEVRAACAIDGYPMFDALLHGASLENAVINPLSEMF<br>LNAQRNLSALNLPVRLFHGAHKTVTTPFAACSETPVLEGLTPRIQRLRNHYLTVRPSVSI<br>YRALAFTEVVKANPGMPAILLRAKAFRHACETAPILIQNDELIVGHPCGKPRAGAFSPDI<br>AWRWVRDELDTMSTRPQDPFEISEEDKKTIREEIVPFWEGRSLDEICEAQYREAGVWSFS<br>GETFVSDLSYHQVNGGGDTCPGYDVLLFTKGMNGIKADAEAHLAELSMENPEDIDRIYYY<br>KAAIETCEGVINYAHRIAARARELAAVEQNAQRRAELLTIAEVNQNVPANPPKTLQEALQ<br>SIWTVESLFEIEENQTGLSLGRVDQYCYPMFEADIREGRLTHEGALELMQAFIIKCAELM<br>WMSSELGAKYFAGYQPFINLTVGGQKRSGGDACNDLTYLIMDAVRFVKVYQPSLACRIHN<br>QSPQKYMEKIVDVVKAGMGFPACHFDDSHIKMMLRKGFDFEDARDYCLMGCVEPQKSGRI<br>YQWTSTGYTQWPIAIEFVLNRGRMVLFDSYQGLDTGDLKDLRTFEDFDAAVKKQVAHIIR<br>LSAIGTVISQRVHRDVAPKPLMSLLVEGCMEKGKDVSAGGAMVNHGPGLIFSGLATYVDS<br>MAAIRKLVYEDKKYTLEQIRDALLANFEGYEGLRRDCLNAPKYGNDDNYVDQYALDITEW<br>TERECRKYKMLYSTLSHGTLSISNNTPIGELTNATPNGRLAWMPLSDGISPTQGADKHGP<br>TAIIKSVSKMNVETMNIGMVHNFKFLKGLLDTPEGRNGLITLLRTASILGNGQMQFSYVD<br>NEVLKKAQQEPEKYRDLIVRVAGYSAYFVELCKEVQDEIISRTVIEKF

Enterobacter	*Enterobacter cancerogenus ATCC 35316*	MKIDALSLRVRQLRDNYLQAKPFVSARRAISVTRVYKENPGMDNTLLRALAFRRACENAP<br>LYVADNELIVSHPAGGARGGEISPEISWRWVADELDTLPQRAQDPYQIDDETKRLLREEV<br>FPYWEGRSLDEMAQTQLQVLGLWEWCHDDGICDVTIKTQNGGGDSCPGYDNILLTKGIKG<br>IREEAAARLAAVDPASPEGAEAFNFYTAMLHTCDGVLTYARRYAARLSELAETEGDPLRQ<br>DELRHLADICRRVPEHPPRHFHDALQAIWFVHSLFTLEENQTGISLGRVDQYLWPLLERD<br>LNDGVLTHAQAEELLCCWLIKMAETLWICSESTAMYFAGYQPFINLVVGGQKREGGDATN<br>PLTLMIMDCSAQLKIYQPGLAVRIHNQSPQPFMRKVVDVVRSGMGFPACHFDDAHIRMML<br>HKGFSYEDARDYCLMGCVEPQKAGKMYQWTSVGYTTFTAAIELALRNGRSSAGKPCGPAT<br>GEVSAFSCYDDVERAVRTQLSAIVRKAAQATLIVQKLHADYARKPLMSSLIDGCMATAKD<br>VTQGGATLNAGPGLIWTGLADCVNSLMAIRTLVFETQRFTLQQVVDALEHNFVGHDDILT<br>ACLRAPKYGNDIRAVDEIARELVRFLEQEHRQYRMLYAPFAFGTLSISNNTPFGLITGAL<br>PSGRLAGKPLADGISPSQQTDYLGPTAIINSVSRINVEEMDIGMVHNIKLMYGMLETPEG<br>QNSLIHLLRTASILGNAQLQFSYVDDDTLRQAQQNPGDYRNLMIRVAGYSAFFVELSKEV<br>QDEIISRTTQRHF

Supplementary Table 5. List of primers for rt-qPCR.

Gene name	forward	reverse
16S	TGTGTAGCGGTGAAATGCG	CATCGTTTACGGCGTGGAC
L.sac	GCAGATGGGCCACTGGGTATA	TCCACAACAAGTCCCCGAAAA
beta (Actb)	GGGAAATCGTGCGTGAC	AGGCTGGAAAAGAGCCT
IL-1β	GCAACTGTTCCTGAACTCAACT	ATCTTTTGGGGTCCGTCAACT
IL-6	CCAAGAGGTGAGTGCTTCCC	CTGTTGTTCAGACTCTCTCCCT
TNF-α	CCCTCACACTCAGATCATCTTCT	GCTACGACGTGGGCTACAG
CD68	TGTCTGATCTTGCTAGGACCG	GAGAGTAACGGCCTTTTTGTGA
F4/80	TGACTCACCTTGTGGTCCTAA	CTTCCCAGAATCCAGTCTTTCC
Ccl 2(MCP-1)	TTAAAAACCTGGATCGGAACCAA	GCATTAGCTTCAGATTTACGGGT
Icam1	GTGATGCTCAGGTATCCATCCA	CACAGTTCTCAAAGCACAGCG
Vcam1	AGTTGGGGATTCGGTTGTTCT	CCCCTCATTCCTTACCACCC
