# Supplementary material for: Integrated metagenomics identifies a crucial role for trimethylamine-producing Lachnoclostridium in promoting atherosclerosis
Source: NPJ Biofilms Microbiomes. 2022 Mar 10;8:11. doi: 10.1038/s41522-022-00273-4 (PMC8913745; doi:10.1038/s41522-022-00273-4)
Supplement: Supplementary file 2 — reporting-summary [file 41522_2022_273_MOESM2_ESM.pdf]

nature portfolio

Corresponding author(s): Jing Li, Lian-wen Qi, Jing Shang

Last updated by author(s): Dec 1, 2021

Reporting Summary

Nature Portfolio wishes to improve the reproducibility of the work that we publish. This form provides structure for consistency and transparency in reporting. For further information on Nature Portfolio policies, see our Editorial Policies and the Editorial Policy Checklist.

Statistics

For all statistical analyses, confirm that the following items are present in the figure legend, table legend, main text, or Methods section.

n/a	Confirmed	
☐	☒	The exact sample size (n) for each experimental group/condition, given as a discrete number and unit of measurement
☐	☒	A statement on whether measurements were taken from distinct samples or whether the same sample was measured repeatedly
☐	☒	The statistical test(s) used AND whether they are one- or two-sided<br>*Only common tests should be described solely by name; describe more complex techniques in the Methods section.*
☒	☐	A description of all covariates tested
☒	☐	A description of any assumptions or corrections, such as tests of normality and adjustment for multiple comparisons
☐	☒	A full description of the statistical parameters including central tendency (e.g. means) or other basic estimates (e.g. regression coefficient) AND variation (e.g. standard deviation) or associated estimates of uncertainty (e.g. confidence intervals)
☐	☒	For null hypothesis testing, the test statistic (e.g. F, t, r) with confidence intervals, effect sizes, degrees of freedom and P value noted<br>*Give P values as exact values whenever suitable.*
☒	☐	For Bayesian analysis, information on the choice of priors and Markov chain Monte Carlo settings
☒	☐	For hierarchical and complex designs, identification of the appropriate level for tests and full reporting of outcomes
☐	☒	Estimates of effect sizes (e.g. Cohen's d, Pearson's r), indicating how they were calculated

Our web collection on statistics for biologists contains articles on many of the points above.

Software and code

Policy information about availability of computer code

Data collection	European Nucleotide Archive (ENA)<br>National Center for Biotechnology Information (NCBI)<br>Human Microbiome Project in September 2014<br>UHGG (Almeida et al. 2020 - PMID 32690973)<br>recapitulated 4,930 species-level genome bins (Pasolli et al. 2019 - PMID 30661755)
Data analysis	MetaGeneMark (v2.8)<br>FASTX-Toolkit (v 0.0.13)<br>SOAPdenovo2 (v 2.04)<br>BLAT (v 35x1)<br>MEGAN (v 5.2.3)<br>BLASTP (v 2.2.29)<br>mafft (v7.455)<br>RAxML (v8.2.12)<br>AutoDock (v.4.2.6)<br>Amber16<br>NanoZoomer 2.0<br>ImageJ<br>R (v 3.6.1)

For manuscripts utilizing custom algorithms or software that are central to the research but not yet described in published literature, software must be made available to editors and reviewers. We strongly encourage code deposition in a community repository (e.g. GitHub). See the Nature Portfolio guidelines for submitting code & software for further information.

Data

Policy information about availability of data

All manuscripts must include a data availability statement. This statement should provide the following information, where applicable:

- Accession codes, unique identifiers, or web links for publicly available datasets
- A description of any restrictions on data availability
- For clinical datasets or third party data, please ensure that the statement adheres to our policy

All data generated in this manuscript are available within the paper and its Supplementary Methods.

Field-specific reporting

Please select the one below that is the best fit for your research. If you are not sure, read the appropriate sections before making your selection.

☒ Life sciences ☐ Behavioural & social sciences ☐ Ecological, evolutionary & environmental sciences

For a reference copy of the document with all sections, see nature.com/documents/nr-reporting-summary-flat.pdf

Life sciences study design

All studies must disclose on these points even when the disclosure is negative.

Sample size	Sample size was chosen based on the prior knowledge from previous experiments (Wang Z, et al. 2015. PMID: 26687352) and according to the minimum experimental requirements and natural factors such as fight-related injury.
Data exclusions	No data was excluded.
Replication	For animal experiments, 10 mice were included for eachl group.
Randomization	All the mice were randomly allocated into different groups.
Blinding	Blinding was not necessary because experimental conditions were well-controlled and experimental results were quantitative and did not require subjective interpretation or analysis.

Reporting for specific materials, systems and methods

We require information from authors about some types of materials, experimental systems and methods used in many studies. Here, indicate whether each material, system or method listed is relevant to your study. If you are not sure if a list item applies to your research, read the appropriate section before selecting a response.

Materials & experimental systems

n/a	Involved in the study	
☒	☐	Antibodies
☒	☐	Eukaryotic cell lines
☒	☐	Palaeontology and archaeology
☐	☒	Animals and other organisms
☒	☐	Human research participants
☒	☐	Clinical data
☒	☐	Dual use research of concern

Methods

n/a	Involved in the study	
☒	☐	ChIP-seq
☒	☐	Flow cytometry
☒	☐	MRI-based neuroimaging

Animals and other organisms

Policy information about studies involving animals; ARRIVE guidelines recommended for reporting animal research

Laboratory animals	Apolipoprotein E knockout mice (C57BL/6J ApoE-/-) were housed in a pathogen-free environment at a temperature of 22-24°C, humidity 40-60% and a strict 12 h light cycle.
Wild animals	This study did not involve wild animals.
Field-collected samples	This study did not involve samples collected from the field.
Ethics oversight	The care and treatment of mice were performed in accordance with the Provisions and General Recommendation of Chinese

Ethics oversight	Experimental Animals Administration Legislation and the study was approved by the Animal Ethics Committee of China Pharmaceutical University (No. 2021-04-002).

Note that full information on the approval of the study protocol must also be provided in the manuscript.
